# *Megalosauripus transjuranicus* ichnosp. nov. A new Late Jurassic theropod ichnotaxon from NW Switzerland and implications for tridactyl dinosaur ichnology and ichnotaxomy

**DOI:** 10.1371/journal.pone.0180289

**Published:** 2017-07-17

**Authors:** Novella L. Razzolini, Matteo Belvedere, Daniel Marty, Géraldine Paratte, Christel Lovis, Marielle Cattin, Christian A. Meyer

**Affiliations:** 1 ICP Institut Català de Paleontologia 'Miquel Crusafont', Mesozoic Research Group, Sabadell, Catalonia, Spain; 2 Office de la Culture, Section D’archéologie et Paléontologie, Porrentruy, Switzerland; 3 Naturhistorisches Museum Basel, Basel, Switzerland; Perot Museum of Nature and Science, UNITED STATES

## Abstract

A new ichnospecies of a large theropod dinosaur, *Megalosauripus transjuranicus*, is described from the Reuchenette Formation (Early–Late Kimmeridgian, Late Jurassic) of NW Switzerland. It is based on very well-preserved and morphologically-distinct tracks (impressions) and several trackways, including different preservational types from different tracksites and horizons. All trackways were excavated along federal Highway A16 near Courtedoux (Canton Jura) and systematically documented in the field including orthophotos and laserscans. The best-preserved tracks were recovered and additional tracks were casted. *Megalosauripus transjuranicus* is characterized by tridactyl tracks with clear claw and digital pad impressions, and notably an exceptionally large and round first phalangeal pad on the fourth digit (PIV1) that is connected to digit IV and forms the round heel area. Due to this combination of features, *M*. *transjuranicus* clearly is of theropod (and not ornithopod) origin. *M*. *transjuranicus* is compared to other *Megalosauripus* tracks and similar ichnotaxa and other unassigned tracks from the Early Jurassic to Early Cretaceous. It is clearly different from other ichnogenera assigned to large theropods such as *Eubrontes*–*Grallator* from the Late Triassic and Early Jurassic or *Megalosauripus*–*Megalosauropus*–*Bueckeburgichnus* and *Therangospodus* tracks from the Late Jurassic and Early Cretaceous. A second tridactyl morphotype (called Morphotype II) is different from *Megalosauripus transjuranicus* in being subsymmetric, longer than wide (sometimes almost as wide as long), with blunt toe impressions and no evidence for discrete phalangeal pad and claw marks. Some Morphotype II tracks are found in trackways that are assigned to *M*. *transjuranicus*, to *M*.? *transjuranicus* or *M*. cf. *transjuranicus* indicating that some Morphotype II tracks are intra-trackway preservational variants of a morphological continuum of *Megalosauripus transjuranicus*. On the other hand, several up to 40 steps long trackways very consistently present Morphotype II features (notably blunt digits) and do not exhibit any of the features that are typical for *Megalosauripus* (notably phalangeal pads). Therefore, it is not very likely that these tracks are preservational variants of *Megalosauripus transjuranicus* or *Megalosauripus* isp. These trackways are interpreted to have been left by an ornithopod dinosaur. The high frequency of large theropod tracks in tidal-flat deposits of the Jura carbonate platform, associated on single ichnoassemblages with minute to medium-sized tridactyl and tiny to large sauropod tracks has important implications for the dinosaur community and for paleoenvironmental and paleogeographical reconstructions. As with most other known occurrences of *Megalosauripus* tracks, *M*. *transjuranicus* is found in coastal settings, which may reflect the preference of their theropod trackmakers for expanded carbonate flats where food was abundant.

## Introduction

*Megalosauripus* can be considered as one of the most widespread Late Jurassic ichnotaxa made by large theropods in Europe, America, and Asia. However, its correct assignment and validity has been highly debated in the last twenty years [1–6]. This is especially because this ichnotaxon represents the typical shape of a large theropod print (tridactyl, longer than wide, narrow), which is morphologically conservative and therefore difficult to characterize and distinguish. *Megalosauripus* is known from Late Jurassic to Early Cretaceous deposits, although it has also been described from the Middle Jurassic of Asia, North America and Europe [3,7–13].

The ichnotaxonomical entanglement started in 1955, when [14] coined the name *Megalosauripus* referring to an illustration of a track from the German collection of Ballerstedt [15], and reproduced in [16], assumed to have been left by a megalosaur dinosaur. Because this track was named after the purported trackmaker, and no proper ichnotaxonomical description was provided, this ichnogenus was declared a *nomen nudum* in [1] and formalized as *Megalosauripus* ichnogen. nov. in [3]. Interestingly, [17] erected the new ichnogenus *Bueckeburgichnus maximus* on the same illustration labelled as *Megalosauripus* in [14]. For this reason, [5] stated that *Megalosauripus* [10] should be considered as a senior synonym of *Bueckeburgichnus*. In addition to the debate about the validity of the ichnotaxon *Megalosauripus* (see [3] vs. [5]), it also turns out that many descriptions are based on rather poorly-preserved material that is in need of revision (see also [18]). The purpose of this paper is to describe various new, and well-preserved large tridactyl tracks from the Late Jurassic of Highway A16 (NW Switzerland) from a morphological point of view. Here we follow the use of *Megalosauripus sensu* [3] because of the morphological affinity of the studied material with the ichnogenus definition and the emended description of [6].

We focus on a large sample of mostly well-preserved, large tridactyl tracks and trackways from the Late Jurassic of NW Switzerland. All studied material has been excavated between 2002 and 2011 and systematically documented by the team ‘Paleontology A16’ on tracksites located on the future course of Swiss federal Highway A16, also named ‘Transjurane Highway’, and on one tracksite located outside Highway A16 in Porrentruy. A part of the material described herein is assigned to the ichnotaxon *Megalosauripus*. Based on the difference of the new material (notably the very large and rounded first phalange on dIV), a new ichnospecies *Megalosauripus transjuranicus* is erected for the *Megalosauripus*-type large theropod trackways from Highway A16 and some additional sites in the Swiss Jura Mountains. Detailed differential diagnoses are provided to underline the difference between previously described ichnotaxa and *Megalosauripus transjuranicus*. The herein described *Megalosauripus* tracks fall into a size range between 35 to 45 cm and are thus smaller than many other *Megalosauripus* tracks [3,19].

Apart from the tracks assigned to *Megalosauripus transjuranicus*, the new material also contains large tridactyl tracks, named ‘Morphotype II’ by [20]. Following this classification, Morphotype II includes those tracks that in the field did not present enough details, and that where grouped without referring to the possible trackmaker (theropod or ornithopod). This morphotype is observed both on track levels associated with *Megalosauripus transjuranicus* (frequently) and on levels without any *Megalosauripus* track. Further analyses of the tracks and trackways showed that, on levels where *M*. *transjuranicus* tracks occur, some trackways with Morphotype II tracks also preserve morphologies that retain some theropod features observed in tracks assigned to *M*. *transjuranicus*, whereas, in clear *M*. *transjuranicus* trackways, some poorly-preserved tracks are very similar to Morphotype II tracks. On the other hand, on track levels without *M*. *transjuranicus* trackways, Morphotype II is consistently observed along very long trackways without displaying any pronounced morphological variability. In these trackways, the consistent morphology of Morphotype II recalls the features of ornithopod tracks. Despite some tracks showing features similar to *Therangospodus pandemicus* [21] and possibly to *Iguanodontipus*? *oncalensis* [22], i.e., the lack of a discrete phalangeal pad formula and claw marks, an accurate diagnosis is prevented by the rather poor track preservation, and therefore they are not ichnotaxonomically assigned.

The revision of the tracks previously assigned to Morphotype II by [20], pinpointed 1) a theropod-like Morphotype II identified on the basis of the best-preserved tracks present in the trackways, and 2) an ornithopod-like Morphotype II described by the consistent morphology of the tracks along very long trackways.

## General setting

### Geographical and geological setting

**Fig 1:**
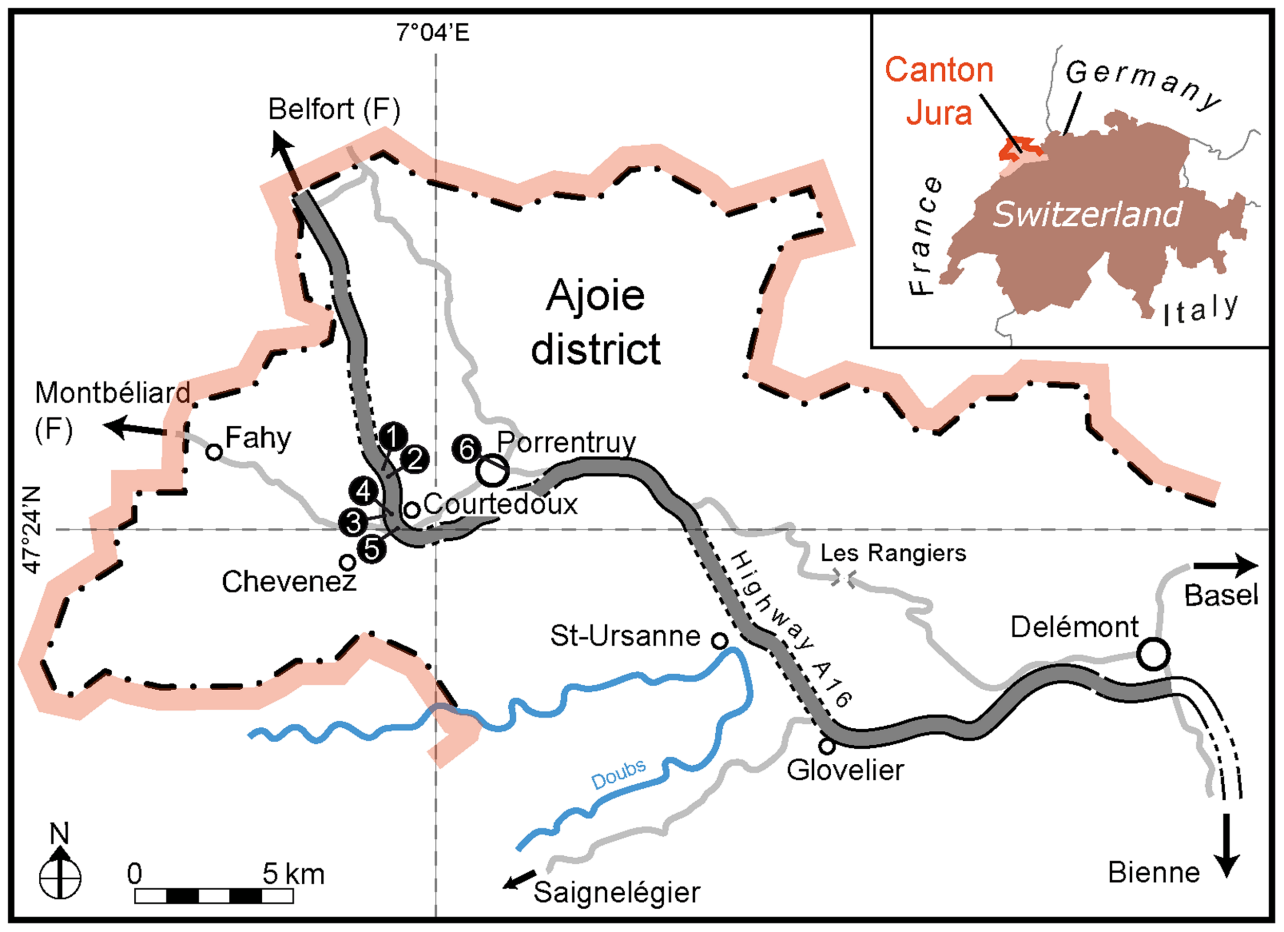
Geographical setting of the Ajoie district (NW Switzerland) and the three Late Jurassic tracksites along Highway A16 (‘Transjurane’). Inset shows location within Switzerland. Numbers indicate the different tracksites: 1. Courtedoux—Béchat Bovais (CTD–BEB), 2. Courtedoux—Bois de Sylleux (CDT–BSY), 3. Courtedoux—Tchâfouè (CTD–TCH), 4. Chevenez—Combe Ronde (CHE–CRO), 5. Courtedoux—Sur Combe Ronde (CTD–SCR), 6. Porrentruy—CPP (POR–CPP).

The studied material comes from one tracksite in Porrentruy (POR–CPP) and from five tracksites, Courtedoux—Béchat Bovais (CTD–BEB), Courtedoux—Bois de Sylleux (CDT–BSY), Courtedoux—Tchâfouè (CTD–TCH), Chevenez—Combe Ronde (CHE–CRO) and Courtedoux—Sur Combe Ronde (CTD–SCR), located about 6 km to the west of Porrentruy (Ajoie district, Canton Jura, NW Switzerland) on the course of Swiss federal highway A16 (Fig 1). These tracksites are situated on a plateau between Courtedoux and Chevenez, and were systematically excavated level-by-level by the team ‘Paleontology A16’ (PALA16) from 2002 until 2011 [20–23]. Today all Highway A16 tracksites are (partially) destroyed and covered up by Highway A16, which was opened for traffic in 2014. The POR–CPP (a.k.a. ‘Dinotec’) tracksite is located in Porrentruy in the backyard of a technical school called ‘CPP’ and was discovered in 2011 during the construction of an additional building. A part of this tracksite in the backyard is now protected and accessible to the public [24].

The study area belongs to the Tabular Jura Mountains, and is located at the eastern end of the Rhine-Bresse transfer zone between the Folded Jura Mountains to the south and east and the Upper Rhine Graben and Vosges Mountains to the north. Elevation is around 500 m and bedding is (sub)horizontal and affected by normal faults created by several tectonic phases during the Cenozoic [25–27].

### Stratigraphy and paleogeography

**Fig 2:**
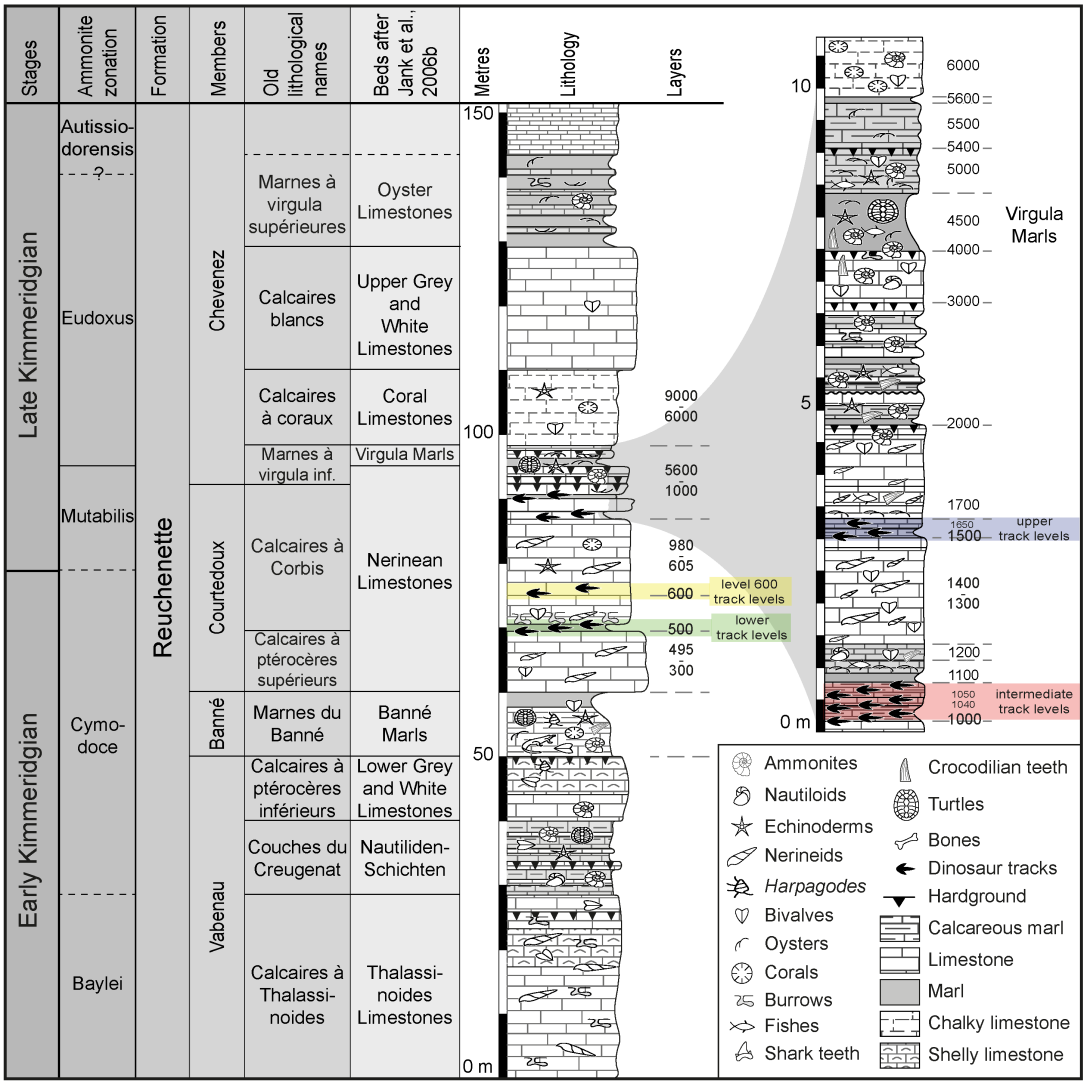
Chrono-, bio- and lithostratigraphic context of the Reuchenette Formation in the Ajoie district, Canton Jura, NW Switzerland (modified from [20,23,28,31]. Four track-bearing intervals, named lower, intermediate, and upper (dinosaur track) levels, and track levels 600 have been identified within the Courtedoux Member (Nerinean Limestones, *sensu* [32]). All studied material comes from the intermediate (levels 1000–1100) and upper (levels 1500–1650) dinosaur track levels, details shown on inset on the upper right.

The studied trackways come from three different track-bearing laminite intervals (named lower, intermediate and upper track levels), separated by shallow marine marls and limestones including massive nerinean limestones [20,28]. The lower track levels are also referred to as levels 500–550, the intermediate one as levels 1000–1100, and the upper ones as levels 1500–1650 (Fig 2).

The track-bearing sequences form part of the Reuchenette Formation [29,30]. Fossil markers such as abundantly occurring ammonites assign the levels to Cymodoce to Mutabilis (Boreal) respectively Divisum to Acanthicum (Tethyan) biozones, i.e., late Early to early Late Kimmeridgian [21,28,31–33]. Some of these ammonites were found in layers very close to the dinosaur track-bearing levels, and the age assignment is also confirmed with ostracods [34].

The sediments of the Reuchenette Formation were deposited at the northern margin of the oceanic Ligurian Tethys on a large, structurally complex carbonate platform, e.g., [35–37]. This Jura carbonate platform was at a paleolatitude of around 30° N, at the threshold between the Paris Basin to the northwest and the Tethys Ocean to the south and thus influenced by both the Tethyan and Boreal realms, e.g., [32,33,35,36,38]. During the Kimmeridgian, the climate of the Jura carbonate platform was semi-arid subtropical to Mediterranean with strong seasonal differences between prolonged, warm, dry summers and relatively short, wet winters, e.g., [39–44]. The presence of freshwater on the platform is corroborated by the occurrence of charophytes [45,46] and hybodontid shark teeth that display an unusual freshwater isotopic signal [47].

The recurrence of dinosaur tracks and emersive phases during the Late Jurassic support the hypothesis–at least during sea-level lowstands–of prolonged periods of emersion of the Jura carbonate platform, which would have connected the larger terrestrial landmasses of the London-Brabant Massif in the northeast and/or the Massif Central in the southwest [20,46,48,49].

### Sedimentology and paleoenvironment

The track-bearing intervals are thinly-bedded, laminated, tabular and platy, marly limestones, which locally have a slightly stromatolitic appearance with intercalations of thin layers of calcareous marls [20]. Generally, the microfacies of the laminites is quite homogeneous and can be described as mudstone to wackestone *sensu* [50], or dolobiopelmicrite *sensu* [51]; the most common biogenic sedimentary structures are (microbial) lamination and invertebrate burrows [20,21].

The track-bearing laminites were deposited in inter- to supratidal flat or supratidal marsh paleoenvironments, characterized by an exposure index of 60–90% [20]. This is indicated by macroscopic (stromatolitic lamination, desiccation cracks, wave ripples, invertebrate burrows) and microscopic (e.g., cryptmicrobial lamination, fenestrae, brecciation) sedimentological features [20,21,52]. Marty [20] suggested that this supratidal-flat paleoenvironment was located several hundred meters away from the coastline towards the open marine realm or an internal lagoon, and that for most of the time was characterized by restricted and hostile conditions, which may have been interrupted by periods of rain or storm surges, and that during or rather at the end of such periods of wetting, dinosaur tracks were recorded.

The lower track levels (500, green band in Fig 2) have a thickness of about 0.6 m and contain at least 8 track-bearing track levels [20]. The intermediate track levels (1000, red band in Fig 2) with a thickness of around 1 m and at least 15 track-bearing levels are the track-richest interval, whereas the upper track levels are about 30–40 thick cm and contain only 2–3 track levels (1500, blue band in Fig 2). The lower track levels are suggested to represent one elementary sequence [20], and the intermediate levels 1–2 elementary sequences of each 20 kyr. The sequence boundary Kim4 was placed in the intermediate levels by [53] in their fig. 10, but probably corresponds to the upper track-bearing levels [54], which again likely represent one elementary sequence.

## Material and methods

### Material

The three letters acronym of the tracksite in combination with the year of discovery is used for the labelling of recovered original tracks (slabs with one to several tracks). For example, TCH006-1140 is the specimen with the number 1140 of the year 2006 of the Courtedoux—Tchâfouè (CTD–TCH) tracksite. In the case of casts, the letter ‘r’ (for French ‘relevé’, or ‘replica’ in English) is added prior to the sample number. Accordingly, SCR008-r129 is the cast (copy) number 129 of the year 2008 from the Courtedoux—Sur Combe Ronde tracksite. With these codes, all the material can unambiguously be identified and located within the collection of the Paleontology A16 (MJSN–PALA16).

In the field, all trackways were excavated, labelled, and mapped at a 1:20 scale. The best tracks were collected on slabs and/or casted. Recovered slabs were stabilized and prepared, and polyester copies were produced. Laserscans with a resolution in the order of several mm and orthophotos with a resolution of around 2 mm were made of several of the studied track-bearing levels (e.g., BEB500, BSY1040, SCR1000). Additionally, selected tracks of the trackway BEB-500-T7 were scanned at a sub-mm resolution with a FARO Platinum Scanarm hand-scanner.

In 2016, measurements of original tracks and casted tracks (by NLR), and outline drawings on transparent Folex monofilm and vectorization (with Adobe Illustrator) were made. High-resolution photogrammetric models were generated from the collected specimens and the casts in the collection, in order allow a detailed documentation and morphological study.

Studied specimens numbers: MJSN-BEB011-r58 (BEB500-TR7-L2, R2), MJSN-BEB011-r54 (BEB500-TR7-R7), MJSN-BEB011-r56 (BEB500-TR7-L10, R10, L11), MJSN-SCR008-r131 (SCR1000-T18-R1), MJSN-SCR008-r129 (SCR1000-T23-R1, L2, R2), MJSN-BSY008-330 (BSY1035-T6-L2), MJSN-BSY008-339 (BSY1040-T1-R1), MJSN-BSY008-338 (BSY1040-T1-L2),MJSN-BSY008-337 (BSY1040-T1-R2), MJSN-BSY008-336 (BSY1040-T1-L3), MJSN-BSY008-334 (BSY1040-T9-R3), MJSN-TCH008-r2 (TCH1000-TR1-R2, L3, R3), MJSN-TCH008-r4 (TCH1000-TR2-R9, L10, R10), MJSN-TCH007-r72 (TCH1000-TR2-L12, R12, L13), MJSN-TCH006-1348 (TCH1015-T1-L2), MJSN-TCH006-1357 (TCH1015-T1-R3), MJSN-TCH006-1366 (TCH1020-T1-R2), MJSN-TCH006-1337 (TCH1020-T2-L1), MJSN-TCH006-1355 (TCH1020-T2-R1), MJSN-TCH006-1140 (TCH1020-T2-L2), MJSN-TCH006-1137 (TCH1020-T2-R2), MJSN-TCH006-1329 (TCH1025-T1-L4, TCH1025-T2-L1), MJSN-TCH006-1023 (TCH1030-T1-R4), MJSN-TCH006-1087 (TCH1030-T2-R2), MJSN-TCH006-1022 (TCH1030-T2-L3), MJSN-TCH006-1034 (TCH1030-T2-R3), MJSN-TCH006-1024 (TCH1030-T3-L1), MJSN-TCH006-1319 (TCH1030-T6-L1), MJSN-TCH006-1317 (TCH1030-T7-L2).

Generally, the quality of the tracks varies a lot, but all the key specimens are amongst the best-preserved ones (preservation quality > 2.5 *sensu* [55]).

All the collected and/or casted specimens, as well as the digital 3D data, are accessible at the PALA16 collections (Office de la Culture, 2900, Porrentruy, Switzerland) and will be transferred to the JURASSICA Muséum (Route de Fontenais 21, 2900, Porrentruy, Switzerland) end of 2018. No permits were required for the described study, which complied with all relevant regulations. A detailed description and interpretation of all the tracks and trackways is provided in S1 Text. S1–S30 Figs are added as visual information to the descriptions and all the measurements are presented in S1 Table.

### Nomenclatural acts

The electronic edition of this article conforms to the requirements of the amended International Code of Zoological Nomenclature, and hence the new names contained herein are available under that Code from the electronic edition of this article. This published work and the nomenclatural acts it contains have been registered in ZooBank, the online registration system for the ICZN. The ZooBank LSIDs (Life Science Identifiers) can be resolved and the associated information viewed through any standard web browser by appending the LSID to the prefix “http://zoobank.org/”. The LSID for this publication is: urn:lsid:zoobank.org:pub:C24529B6-D947-47E5-B8BE-96AC74FD402F. The electronic edition of this work was published in a journal with an ISSN, and has been archived and is available from the following digital repositories: PubMed Central, LOCKSS.

### Track and trackway parameter measured in the field

**Fig 3:**
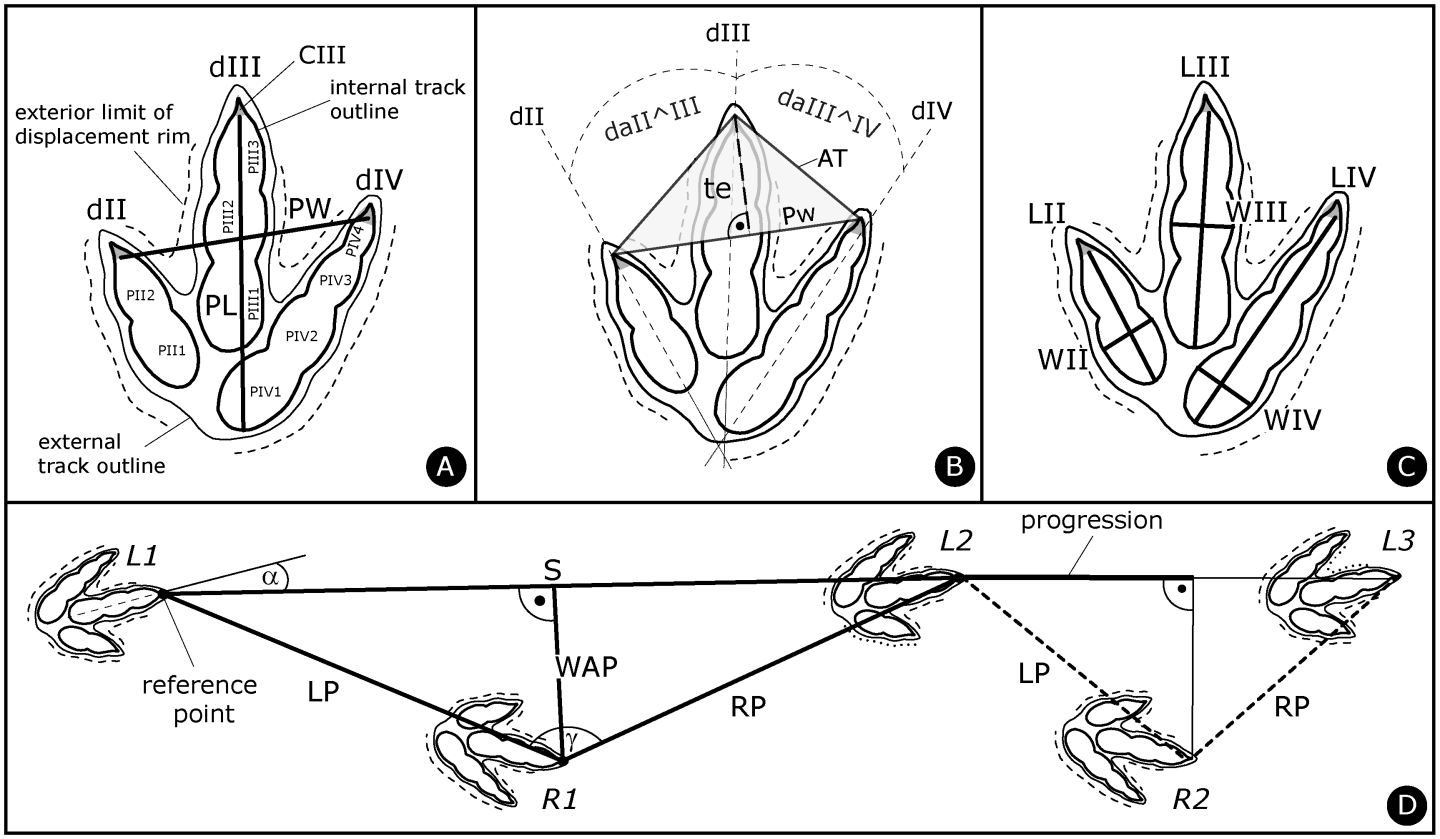
Methodology of track and trackway labeling and parameter measurements. Note that the pictured tridactyl track does not correspond to *Megalosauripus transjuranicus*, it is a schematic track with a typical theropod phalangeal pad configuration of 2-3-4 for dII-III-IV, respectively. **(A)** Track length (PL) and width (PW), labeling of digits (d), phalangeal pads (P) and claws (C). The internal track outline corresponds to the (interpretation of the) actual impression of the foot. **(B)** Interdigital angles (da) and anterior triangle (AT). PW is the width and te the length (measured perpendicular to the width) of the anterior triangle, which in the present case has an obtuse angle for the anterior apex indicating a low mesaxony. **(C)** Digit lengths (L) and widths (W). **(D)** Trackway parameters. Labeling of trackways always starts with L1; if L1 is missing R1 is the first number used. α is the rotation (in this case outward and thus a positive value) of the track (long axis) with respect to the next stride line. LP and RP are left and right pace, respectively; S is stride; WAP is width of the angulation pattern (measured perpendicular to the stride length; [20]), γ is pace angulation. The progression is a calculated value (with the Pythagoras’s theorem) and it indicates the forward movement of the trackmaker in the direction of the trackway during one footfall (pace) [20]. Progression is only half of the stride in the case of completely regular trackways. The reference point for the trackway parameter measurements is on the tip of the third digit (without the claw where preserved).

In the field, track (Fig 3A) and trackway parameters (Fig 3D) were systematically measured following standard ichnological terminology, e.g., [20,56,57]. All data, including mean and standard deviations per trackway parameters, are provided in S1 Table. The following abbreviations are used: PL: Pes Length; PW: Pes Width; PaL: Pace Length; SL: Stride Length; PA: Pace Angulation; WAP: Width of the Angulation Pattern.

For trackway parameter measurements, the distal end of the third digit (and not the tip of the claw) is used as reference point. Tracks directed outward with respect to the line connecting it with the consecutive track (the stride) have an outward (positive) rotation and those directed inward an inward (negative) rotation. For the quantification of trackway gauge (trackway internal width), the ratio between the width of the angulation pattern and the corresponding track length ([WAP/PL]-ratio) is used (see also [20,58]). All studied theropod trackways have a [WAP/PL]-ratio < 1.0 and are thus narrow gauge. Trackways with a [WAP/PL]-ratio < 0.5 are considered as ‘very narrow gauge’ whereas trackways with a [WAP/PL]-ratio > 0.5 (the minority of the trackways) are described as ‘comparatively wide’ in comparison with the other trackways; they are still narrow gauge in as far that pes tracks intersect or touch the trackway midline.

Trackways as interpreted in the field were mapped and are illustrated by outline drawings exhibiting the distinct and essential characters of the tracks. In the trackway outline drawings, the internal track outline marks the actual imprint (impression) of the foot and defines the track dimensions (length, width), whereas the external track outline and the external limit of the displacement rim define the extramorphological features of the tracks (Fig 3A).

According to [20] (table 2.2), the following division of maximum pes length (PL) in cm is used to address size classes of tridactyl dinosaur tracks: minute (PL<10 cm); small (10<PL<20 cm); medium-sized (20<PL< 30 cm); large (30<PL< 50 cm); and giant (PL> 50 cm).

### Track measurements in the collection

Detailed track measurements (phalanges, claws) were carried out on original material and casts (copies) of the holotype, paratypes and referred specimens stored in the PALA16 collection and this data is given in S1 Table. Track (pes) length (PL) is measured from the maximum distal point of digit III (anterior point of PIII3, excluding the claw mark where preserved) to the maximum proximal point of the first phalangeal pad of digit IV (PIV1) or the metatarso-phalangeal pad impression when present (Fig 3A). Track (pes) width (PW) is measured between the tips of the lateral digits II and IV (Fig 3A); and not between the tip of the claw marks even if preserved.

The anterior triangle (AT), originally defined by [59] (fig. 2), is measured between the distal ends of the three digits (Fig 3B) following [60], and not between the tips of the three claw marks as proposed by [59] because the claws are often variably preserved and/or mostly not preserved on all three digits. The maximum height of the triangle (te) is measured, perpendicular to the transverse base of the triangle (corresponding to PW) and its length/width-ratio ([te/PW]-ratio) is calculated following the definition of [59], who called this ratio ‘toe extension/foot width-ratio’, used to characterize how much digit III projects anteriorly beyond lateral digit IV and medial digit II as expressed by polarity between strong mesaxony (strong central tendency) and weak mesaxony (weak central tendency) [60].

Interdigital angles (da) are measured from the intercepting lines dividing the digits in halves (Fig 3B). These lines are also used as guides when measuring digit II, III and IV lengths and widths (Fig 3C) and phalangeal pad (numbered from proximal to distal as in Fig 3A) PII1/2, PIII1/2/3 and PIV1/2/3/4 lengths. Digit and phalangeal pad widths are measured tracing a line at the point of greatest width perpendicular to the long axis (intercepting line) of the digit or phalangeal pad impression.

### Calculation of locomotion speed

Calculation of locomotion speed (v) derives from the empiric relationship (v≃0.25g^0.5^SL^1.67^h^-1.17^; SL = stride, h = hip height, g = acceleration of free fall) obtained by [61] and for the calculation of hip height the factor 4.9 is used: h = 4.9 x PL [57,62]. Because of several shortcomings of this empiric relationship due to the estimation of hip height based on tracks and the *a priori* unknown precise trackmaker, e.g., [63,64], as well as the unknown precise relationship between relative stride length (SL / h) and the Froude number (speed^2^ / leg length x g) for dinosaurs [65], speed calculations are considered rough approximations only [65,66]. Nonetheless, Alexander’s method [61] is at least informative providing an estimation for the magnitude of the locomotion speed of a dinosaur trackway and, more importantly, for the relative speed of a given sample of trackways. All values listed in S1 Table.

### Photogrammetry

The photogrammetric 3D models were obtained using a Canon EOS 70D, 20 Megapixel, camera, equipped with a Canon 10-18mm STS or a Canon 18-135mm STS lens and a Canon ring flash (Macro Ring Lite MR-14 EXII) in order to eliminate the shadows generated by sunlight. Models were created using Agisoft Photoscan Pro (v. 1.2.4 and v. 1.2.5; www.agisfot.com) following the procedures of [67,68]. The accuracy of the models ranges between 0.1 and 0.03 mm, and resolution is always sub-millimetric. Scaling and alignment was made in Photoscan Pro. The scaled mesh, exported Stanford PLY files, were then processed in CloudCompare (www.cloudcompare.com), where the meshes where accurately oriented through the generation of a plane intersecting the surface, to avoid imprecise alignment due to the roughness and irregularity of the surface, then it was possible to create accurate false colour depth-maps. Rhinoceros (v. 5.12) was then used to create contour lines.

All photogrammetric and laserscanner 3D meshes used in this study, and the related quality reports, are available for download here: https://doi.org/10.6084/m9.figshare.4036584 (approximately 5 GB).

### Systematic ichnology

Ichnofamily: Eubrontidae Lull [69]

Ichnogenus: *Megalosauripus* Lessertisseur [14] (*sensu* [3])

Synonymy: For detailed synonymy lists refer to [3] and [5].

*Megalosauripus transjuranicus* ichnosp. nov.

urn:lsid:zoobank.org:pub:C24529B6-D947-47E5-B8BE-96AC74FD402F

**Fig 4:**
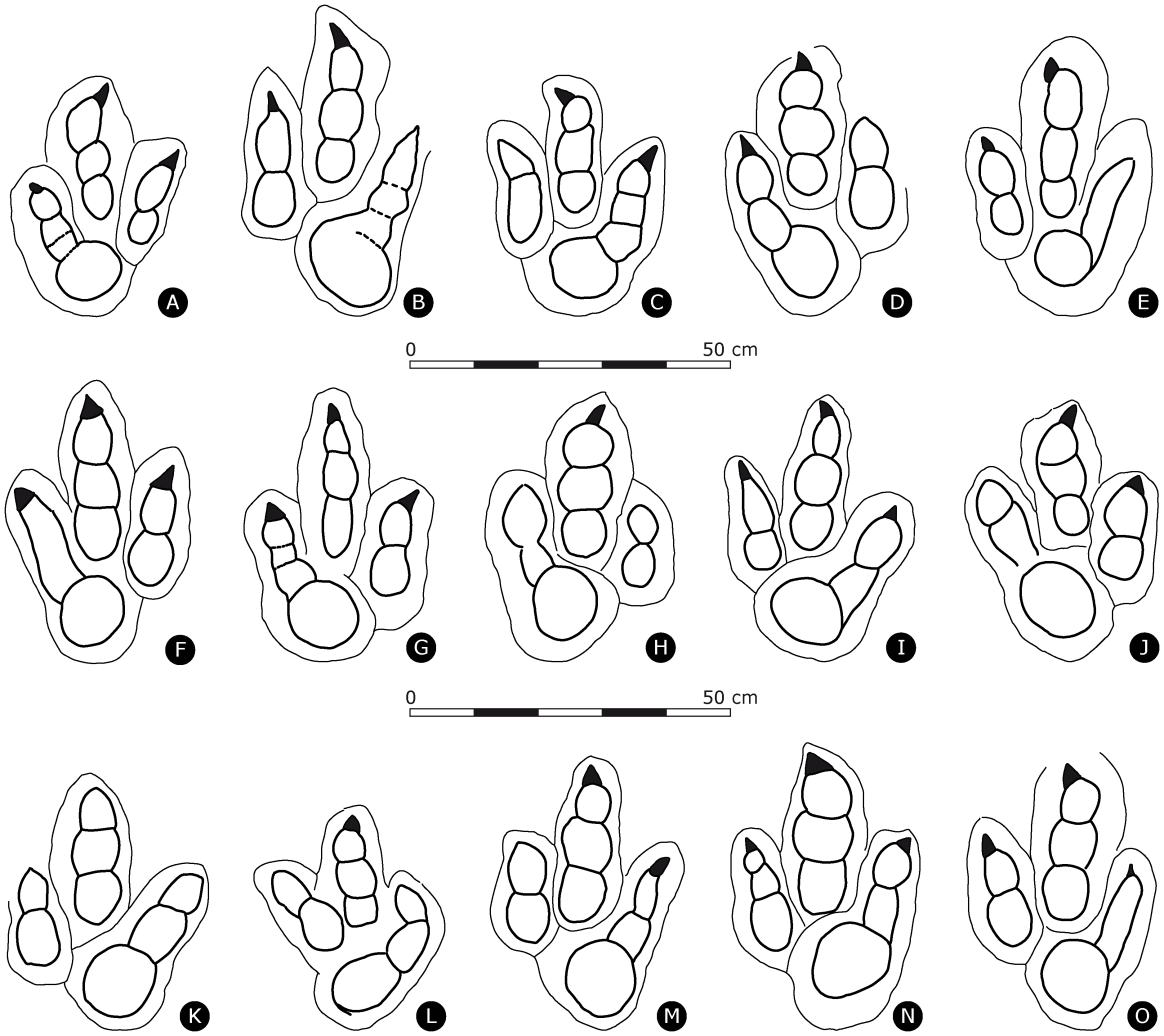
Outline drawings of *Megalosauripus transjuranicus* footprints. **(A)** TCH1030-T6-L1, holotype. **(B)** BSY1035-T6-L2, paratype. **(C)** BSY1040-T1-R1, paratype. **(D)** TCH1025-T2-L1, paratype. **(E)** TCH1030-T2-R2, paratype; **(F)**TCH1030-T2-L3, paratype. **(G)** TCH1030-T7-L2, paratype. **(H)** BSY1040-T1-L2. **(I)** BSY1040T1-R2. **(J)** BSY1040-T1-L3. **(K)** BSY1040-T9-R3. **(L)** TCH1015-T1-L2. **(M)** TCH1015-T1-R3. **(N)** TCH1030-T3-L1. **(O)** TCH1030-T2-R3.

**Fig 5:**
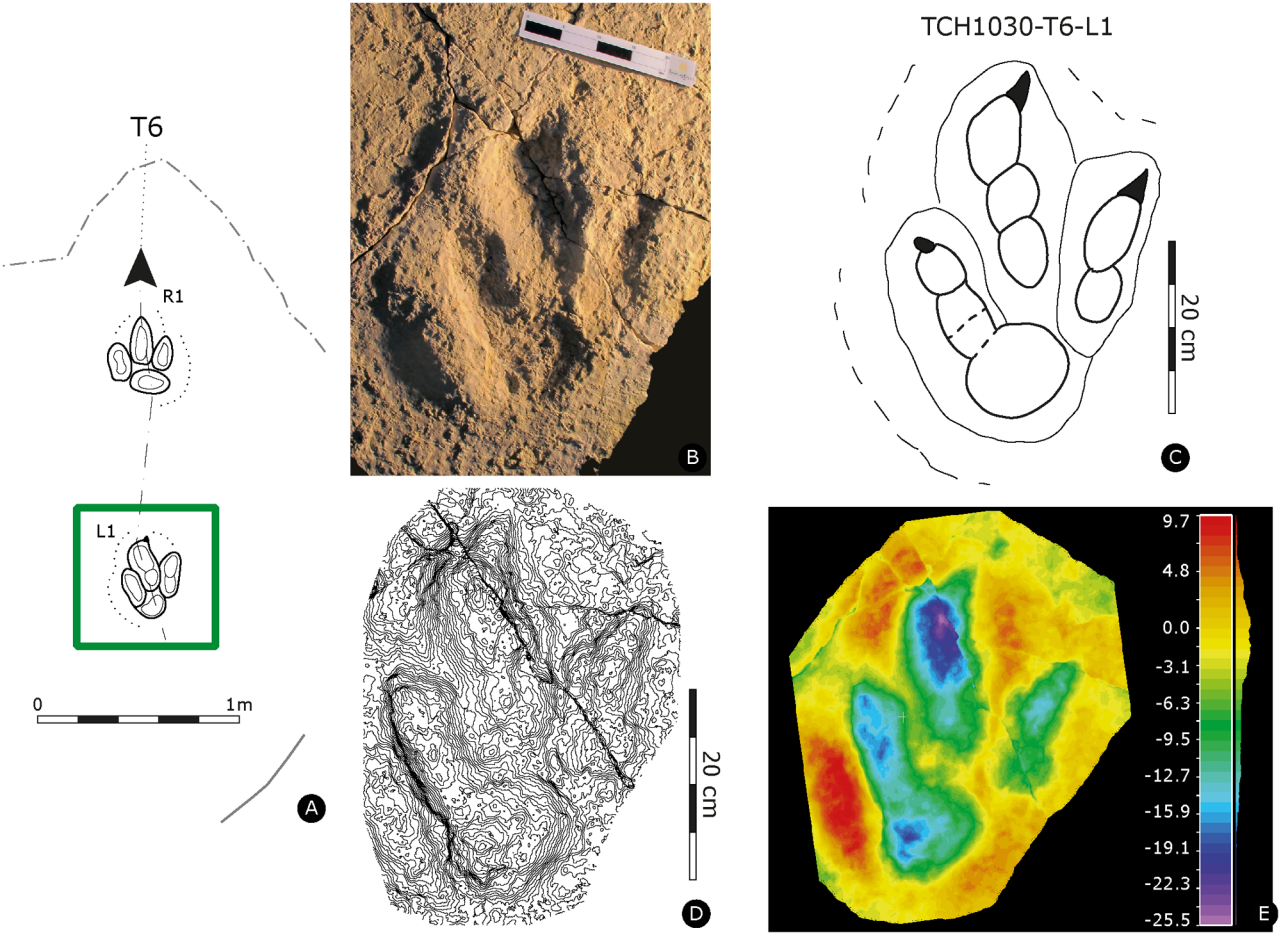
Holotype of *Megalosauripus transjuranicus*. Specimen TCH1030-T6-L1 (MJSN-TCH006-1319). **(A)** Trackway representation. **(B)** Photo of the specimen. Scale 20 cm. **(C)** Interpretative outline drawing. **(D)** Contour-lines. Spacing 1 mm. **(E)** False-color depth map. Depth measured in mm.

**Fig 6:**
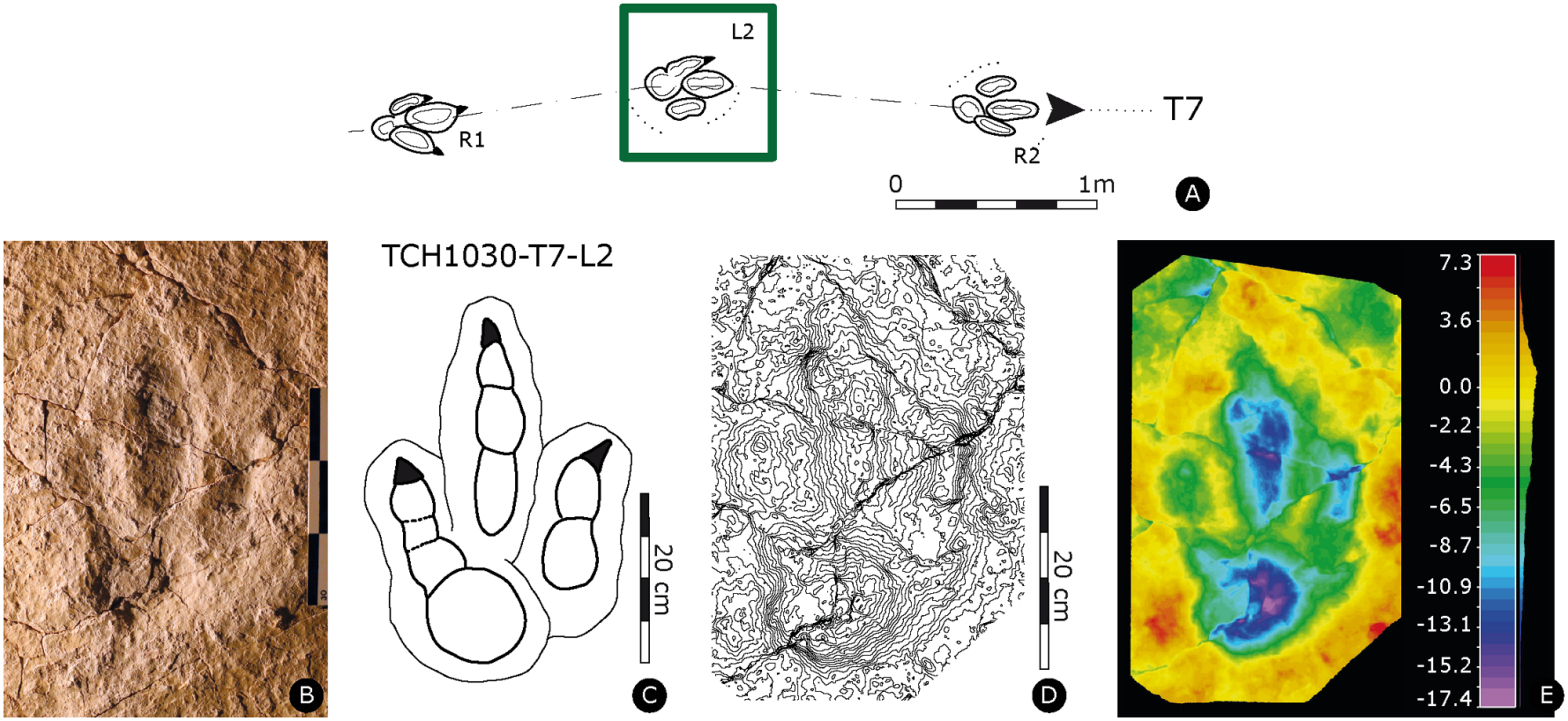
Paratype of *Megalosauripus transjuranicus*. Specimen TCH1030-T7-L2 (MJSN-TCH006-1317). **(A)** Trackway representation. **(B)** Photo of the specimen. Scale 30 cm. **(C)** Interpretative outline drawing. **(D)** Contour-lines. Spacing 1 mm. **(E)** False-color depth map. Depth measured in mm.

**Fig 7:**
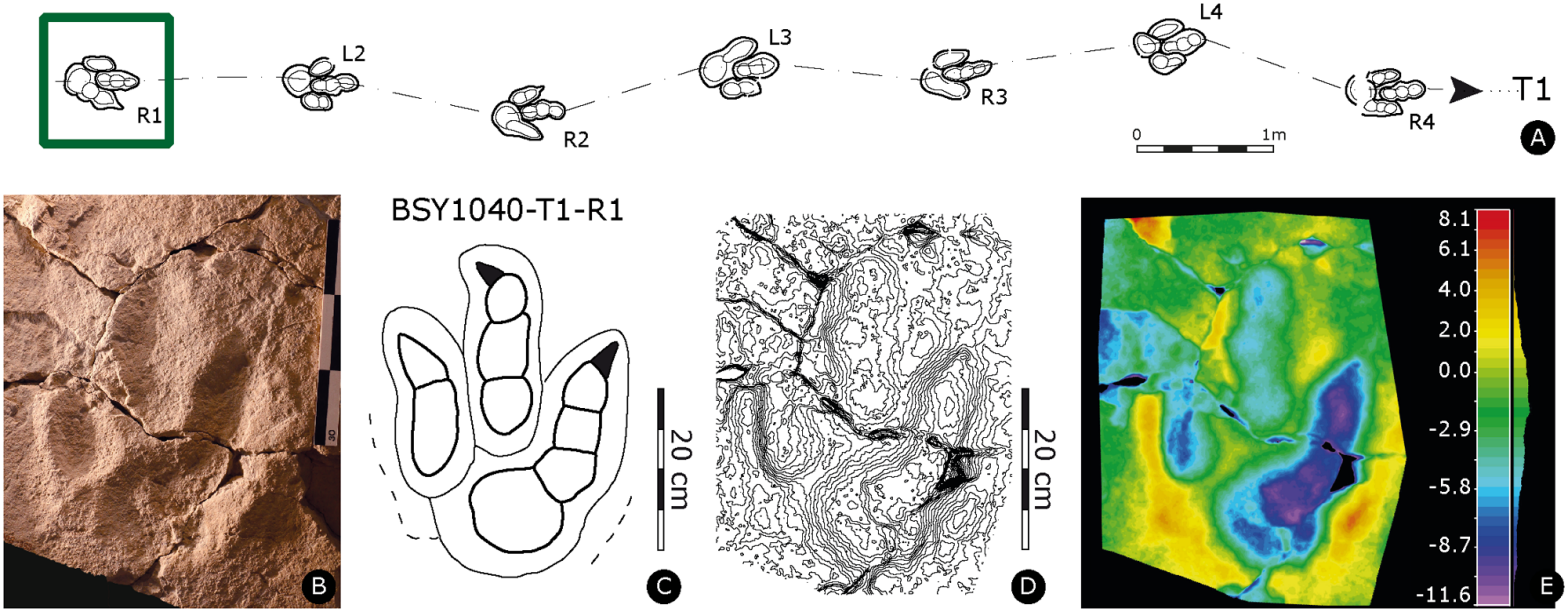
Paratype of *Megalosauripus transjuranicus*. Specimen BSY1040-T1-R1 (MJSN-BSY008-339). **(A)** Trackway representation. **(B)** Photo of the specimen. Scale 30 cm. **(C)** Interpretative outline drawing. **(D)** Contour-lines. Spacing 1 mm. **(E)** False-color depth map. Depth measured in mm.

**Fig 8:**
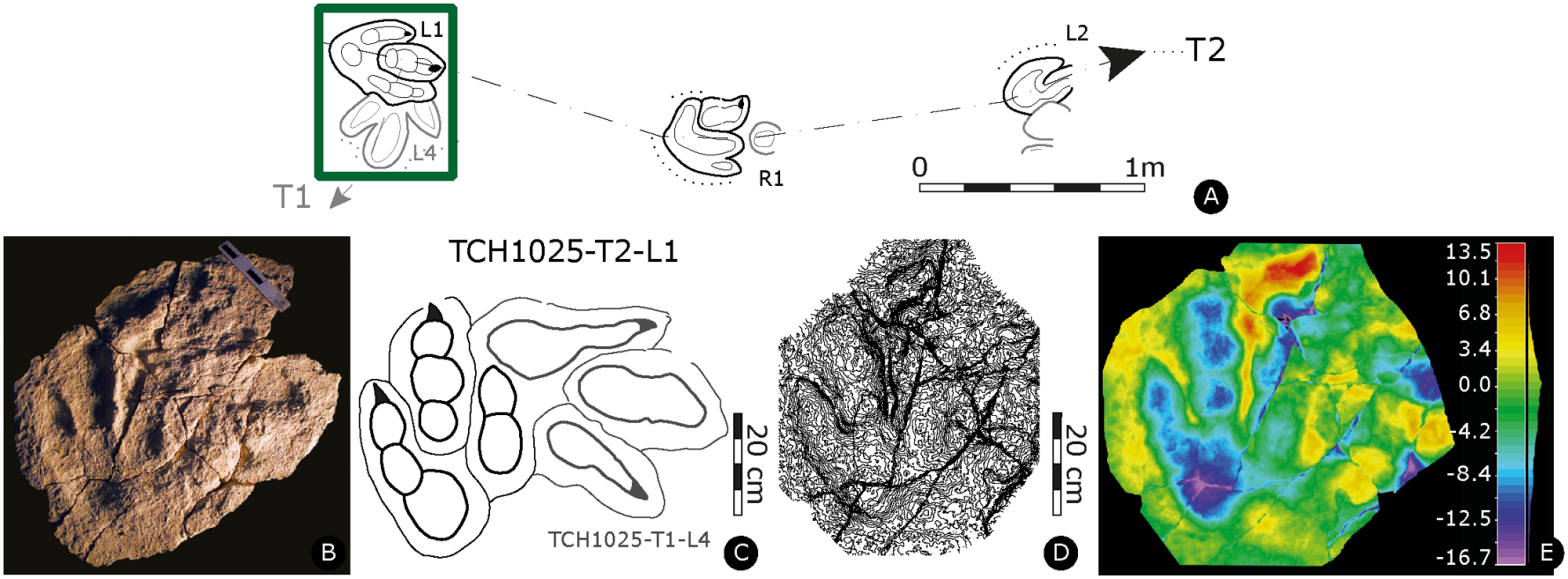
Paratype of *Megalosauripus transjuranicus*. Specimen TCH1025-T2-L1 (MJSN-TCH006-1329). **(A)** Trackway representation. **(B)** Photo of the specimen. Scale 20 cm. **(C)** Interpretative outline drawing. **(D)** Contour-lines. Spacing 1 mm. **(E)** False-color depth map. Depth measured in mm.

**Fig 9:**
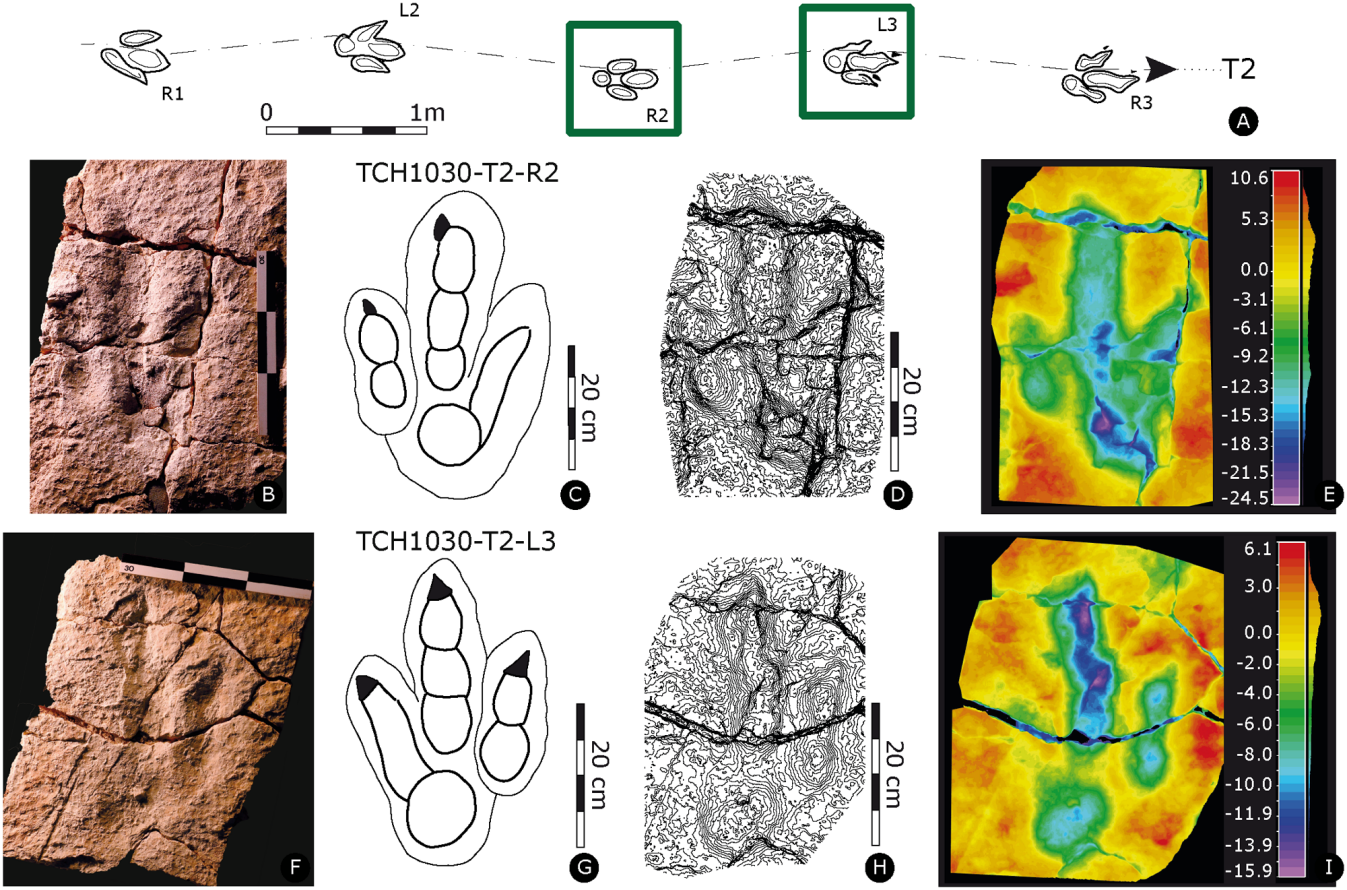
Paratypes of *Megalosauripus transjuranicus*. **(A)** Trackway representation. **(B-E)** Specimen TCH1030-T2-R2 (MJSN-TCH006-1087). **(B)** Photo of the specimen. Scale 30 cm. **(C)** Interpretative outline. **(D)** Contour lines. **(E)** False-color depth map. Depth measured in mm. **(F-I)** Specimen TCH1030-T2-L3 (MJSN-TCH006-1022). **(F)** Photo of the specimen. Scale 30 cm. **(G)** Interpretative outline. **(H)** Contour-lines. Spacing 1 mm. **(I)** False-color depth map. Depth measured in mm.

**Fig 10:**
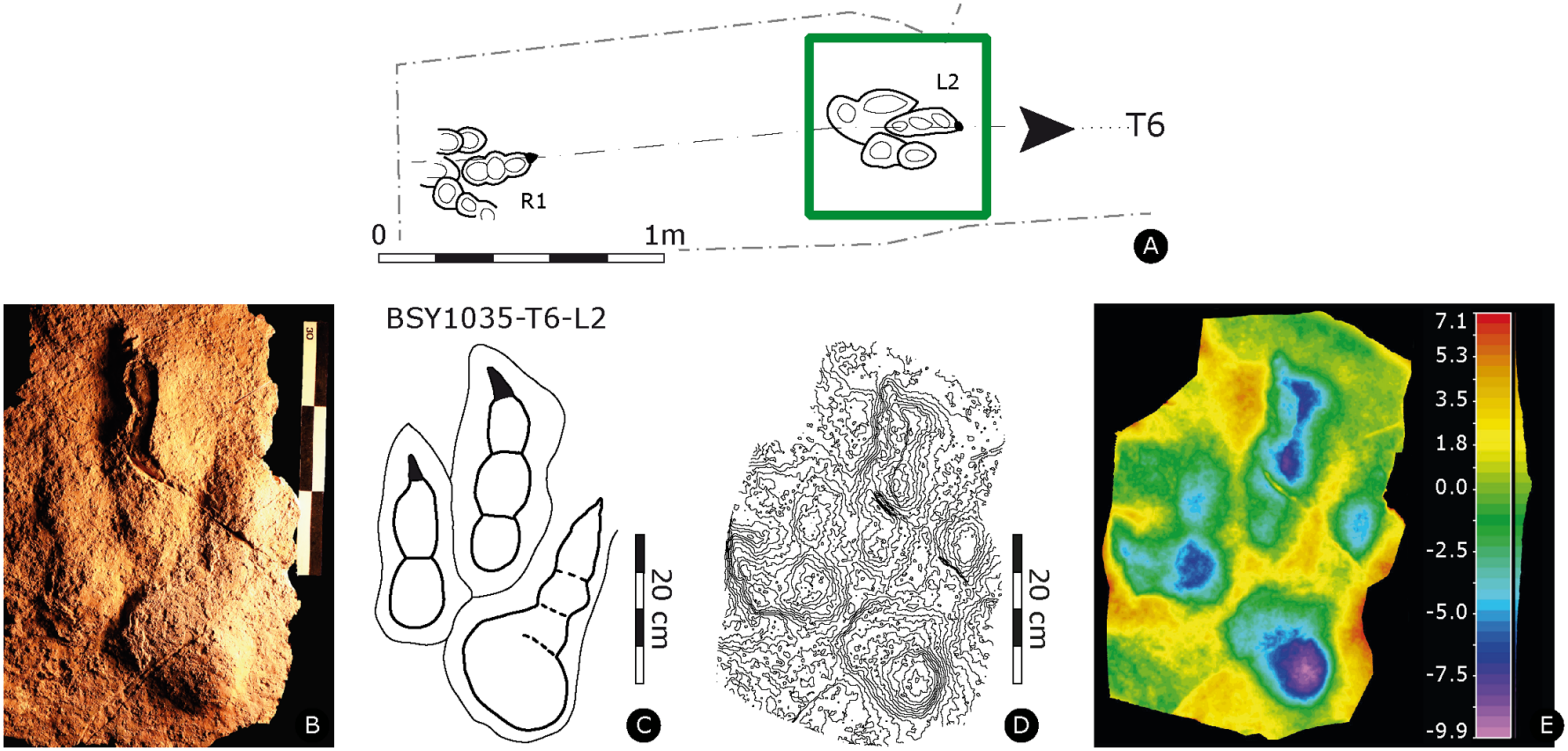
Paratype of *Megalosauripus transjuranicus*. Specimen BSY1035-T6-L2 (MJSN-BSY008-330). **(A)** Trackway representation. **(B)** Photo of the specimen. Scale 30 cm. **(C)** Interpretative outline drawing. **(D)** Contour-lines. Spacing 1 mm. **(E)** False-color depth map. Depth measured in mm.

Figs 4, 5, 6, 7, 8, 9 and 10

Diagnosis of *Megalosauripus* in [1]: large (length>40 cm to a maximum of 80 cm), elongate (average length:width ratio 1:2), asymmetric tridactyl tracks; clear pad impressions that match the typical theropod phalangeal formula (2:3:4 corresponding to digits II, III and IV); sigmoidal impression of digit III; ungueal impression of digit I oriented posteriorly and medially; large impression of the metatarsal phalangeal pad of digits II and III and average divarication angle between digit II-III of 40°,and between digits III-IV of 30°. Trackway show irregular track morphology, with variable steps length and pace angulation; prominent inward rotation with respect to the trackway midline of the distal end of digit III.

### Etymology

In analogy to Highway A16, also called ‘Transjurane’ in French. All dinosaur track excavations prior to the construction of the highway were financed by 95% by the ASTRA (Swiss Federal Roads Authority), and herewith we want to acknowledge this important and unique contribution to paleontology in Switzerland. Trans from Latin meaning across, through or bandura stands for the provenance (Jura Mountains, Jura Canton), and is derived from the Celtic/Gaulish word ‘Jor’ meaning forest or ‘mountains with forest’. The fossil-rich limestones of the Jura Mountains, which was called by [70] ‘Jura Kalkstein’, are the basis of the name of the Jurassic Period [71,72].

### Holotype

TCH1030-T6-L1 (original specimen, collection no.: MJSN-TCH006-1319).

### Paratypes

BSY1035-T6-L2 (original specimen, collection no.: MJSN-BSY008-330), BSY1040-T1-R1 (original specimen, collection no.: MJSN-BSY008-339), TCH1025-T2-L1 (original specimen, collection no.: MJSN-TCH006-1329), TCH1030-T2-R2 (original specimen, collection no.: MJSN-TCH006-1087), TCH1030-T2-L3 (original specimen, collection no.: MJSN-TCH006-1022), TCH1030-T7-L2 (original specimen, collection no.: MJSN-TCH006-1317).

### Referred specimens

All referred specimens (tracks) are preserved as an original specimen. TCH1015-T1-L2 (MJSNTCH006-1348), TCH1015-T1-R3 (MJSN-TCH006-1357), TCH1030-T2-R3 (MJSN-TCH006-1034), BSY1040-T1-L2 (MJSN-BSY008-338), BSY1040-T1-R2 (MJSN-BSY008-337), BSY1040-T1-L3 (MJSN-BSY008-336), BSY1040-T9-R3 (MJSN-BSY008-334).

### Diagnosis

Functionally tridactyl, asymmetrical track, clearly longer than wide ([PL/PW]-ratio ranges from 1.17 to 2.02, track length ranges from 35.5 to 44.5 cm, with a moderate mesaxony ([te/PW]-ratio ranges from 0.35–0.73). Slender digits are well separated, often by small sediment ridges. Digit IV is the longest, followed by dIII and dII. Digit III is the widest, followed by dIV and dII. Tracks exhibit the typical theropod phalangeal pad formula of 2-3-4 corresponding to digits II, III and IV [57] in well-preserved tracks, while in slightly less well-preserved tracks, PIV3 and PIV2 are often not clearly discernible. PIV1 is very characteristic, as it has a circular (rounded) shape, it is the widest and largest phalangeal pad and is connected to the rest of dIV impression as it forms the round heel of the tracks. PIV1 is generally twice the width of the rest of dIV impression. Presence of well-marked and elongated claws, straight or sometimes inwardly/outwardly oriented on the tips of all three digits II-III-IV. Below digit II (between dII and dIV) a postero-medial indentation (notch) is well developed. Digit III impression is straight to sigmoidal; dII impression is generally inwardly oriented. Tips of dII and dIV can sometimes be on a line perpendicular to the long axis of dIII. Tips of digits II and IV can sometimes be on a line perpendicular to the long axis of digit III. Digit IV impression is the shallowest of the track. There is no evidence for a digit I (hallux) impression.

Trackway configuration is generally quite regular; paces are subequal in length between the right and left sides, with no significant differences registered. Pace length ranges from 83 to 150 cm, pace angulation from 160° to 176°, and stride length from 235 to 301 cm. The gauge is variable with a [WAP/PL]-ratio of 0 to 0.5 indicating a trackmaker with a (very) narrow posture.

### Distribution

Late Jurassic (Kimmeridgian).

### Type locality

Courtedoux—Tchâfouè and Courtedoux—Bois de Sylleux tracksites, Ajoie district, Canton Jura, NW Switzerland.

### Type horizon

Intermediate track-bearing levels [20,23] of the Nerinean limestones *sensu* Jank [32,33] of the Courtedoux Member [28] of the Reuchenette Formation [29].

### Age

Tethyan Divisum to Acanthicum ammonite zones, late Early to early Late Kimmeridgian, Late Jurassic [21,28,31–33].

### Holotype description

#### TCH1030-T6-L1

Left pes track (MJSN-TCH006-1319, Fig 5, S29 Fig). Tridactyl, asymmetrical track with slender and well-segmented digits, with a phalangeal pad formula of 2-3-4 and with well-marked and inwardly-rotated digit II claw marks. Digits are well separated, with well discernible sediment ridges between digits II-III and III-IV, and sediment displacement rims surrounding the track. The track is deep, especially digit IV, which is deeper than digits II digit III. The track is narrow, with asymmetric and low interdigital angles (29° for II^III and 22° for III^IV). The track is longer than wide ([PW/PL]-ratio = 1.5), the mesaxonic index is medium and not extremely pronounced ([te/PW]-ratio = 0.58). Presence of wrinkle marks is due to growth of microbial mats, as also displayed by the well-laminated track-bearing layer. Two pads compose digit II. PII2 is longer and bigger than PII1 (proximal). There is a pronounced postero-medial indentation below the digit II impression. Digit III has a sigmoidal shape, and digit IV has four visible phalanges, whereas PIV1 is circular (well rounded), deep, and it is the largest phalangeal pad impression. It is also the deepest part of the track and forms the rounded heel of the track.

### Paratypes descriptions

#### TCH1030-T7-L2

Left pes track (MJSN-TCH006-1317, Fig 6, S30 Fig). Tridactyl, asymmetrical track, slender and well-segmented digits, phalangeal formula 2-3-4 with well-marked and forwardly-directed claw marks. On digit II the claw mark is slightly inward oriented. Digits are well separated, with well-discernible sediment ridges between II-III and III-IV. The track is narrow, interdigital angles are asymmetric and low (9° for II^III and 21° for III^IV). The track is clearly longer than wide ([PW/PL]-ratio = 1.85), the mesaxonic index is medium and not extremely pronounced ([te/PW]-ratio = 0.73). The track still preserves some track fillings. Digit II is composed of two pads, whereas PII2 is longer and bigger than PII1 (proximal). There is a pronounced postero-medial indentation below digit II impression. In digit III, PIII2 is longer than the proximal and distal pads. Digit IV has four clearly visible phalanges. PIV1 is well rounded, deep and large. It is the deepest part of the track. Furthermore, the impression of digit IV is shallower than digits II and III. PIV2 and PIV3 are not well discernible and not very big (marked as fused in S1 Table). PIV2-3-4 are shallower than PIV1.

#### BSY1040-T1-R1

Right pes track (MJSN-BSY008-339, Fig 7, S14 Fig). Very shallow, tridactyl, asymmetrical track, with slender and well-separated digits. All phalanges, except for PIV3 and PIV2, are well discernible. Claw marks are slender and comparatively short, probably due to the firmness of the substrate, as reflected by the shallowness of the track. Presence of a pronounced postero-medial indentation below dII. Narrow track with asymmetric and low interdigital angles (5° for II^III and 14° for III^IV). The track is longer than wide (PL/PW = 1.8), the mesaxonic index is medium and not extremely pronounced ([te/PW]-ratio = 0.6). The slab is characterized by a wrinkled surface (‘wrinkle marks’), most likely due to the former presence of microbial mats [73,74].

#### TCH1025-T2-L1

Left pes track (MJSN-TCH006-1329, Fig 8, S24 Fig). Tridactyl, asymmetrical track, with digits III and IV very well preserved and digit II in interference with track TCH1025-T1-L4 (preserved on the same slab MJSN-TCH006-1329) and therefore not clearly discernible. Digits are well separated by very steep, pronounced and narrow sediment ridges between digits II-III, suggesting that sediment was squeezed between the two digits. Claws are forwardly directed forming shapes recalling isosceles triangles. Track is narrow, interdigital angle is asymmetric and low (6° for II^III and 20° for III^IV). The track is longer than wide ([PW/PL]-ratio = 1.8), the mesaxonic index is medium and not extremely pronounced ([te/PW]-ratio = 0.51). Presence of wrinkle marks is due to the growth of microbial mats [73,74], as displayed also by the finely-laminated track-bearing layer.

#### TCH1030-T2-R2

Right pes track (MJSN-TCH006-1087, Fig 9B–9E, S26 Fig). Tridactyl, asymmetrical track, with slender and well-separated digits. Digits appear to be well impressed as the track shows a certain depth and digit IV impression is much shallower with respect to the other digits. All phalanges, apart from PIV3 and PIV2, are well discernible. Impression of PIV1 phalangeal pad is rounded and it measures 10 cm in diameter. Digits II-IV distal endings impressions are not aligned. Digits II-III impressions are inwardly rotated. Claw marks are slender and well developed, forming isosceles triangle shape in correspondence of digit II and scalene triangle shapes for digit III impression. No claw mark is observed in correspondence to digit IV impression. The interdigital angles are asymmetric (18° for II^III and 30° for III^IV). The track is longer than wide ([PW/PL]-ratio = 1.5), the mesaxonic index is medium and not extremely pronounced ([te/PW]-ratio = 0.6). The slab is characterized by a wrinkled surface (wrinkle marks), most likely due to the former presence of microbial mats [73,74].

#### TCH1030-T2-L3

Left pes track (MJSN-TCH006-1022, Fig 9F–9I, S21 Fig). Shallow, tridactyl, asymmetrical track, with slender and well-separated digits. All phalanges, except for PIV3 and PIV2, are well discernible. Impression of PIV1 phalangeal pad is rounded and measures 10 cm in diameter. Digit IV impression is the shallowest. Digits II-IV distal endings impressions are slightly diverging from one another, meaning that they are not aligned. Digits II-III impressions are inwardly rotated. Claw marks are slender and well developed, forming a scalene triangle shape in digit II and isosceles shapes for digits III and IV impressions. The interdigital angles are subequal in divergence (19° for II^III and 19° for III^IV). The track is longer than wide ([PW/PL]-ratio = 1.67), the mesaxonic index is medium and not extremely pronounced ([te/PW]-ratio = 0.58). The slab is characterized by a wrinkled surface (wrinkle marks), most likely due to the former presence of microbial mats [73,74].

#### BSY1035-T6-L2

Left pes track (MJSN-BSY008-330, Fig 10, S13 Fig). Tridactyl, asymmetrical track cast, with slender and well-separated digits. Digits appear to be well impressed as the track shows a certain depth and the digit IV impression is somewhat shallower with respect to the other digits, although at least PIV1, PIV2 and PIV4 phalangeal pad impressions are appreciable. Phalangeal pad impressions for all digits are well discernible, except for PIV3. Impression of PIV1 phalangeal pad is rounded and very well developed and impressed, measuring 11.5 cm in diameter. Digits II-IV distal endings impressions are not aligned and digit II impression is slightly inwardly oriented, with a subparallel orientation with respect to digit III impression. Claw marks are slender and well developed, forming isosceles triangle shape in correspondence of digit II and inwardly oriented ‘D-shape’ for digit III impression. No claw mark is observed in correspondence to digit IV impression. The interdigital angles are subequal and narrow (10° for II^III and 15° for III^IV). The track is much longer than wide ([PW/PL]-ratio = 1.8), the mesaxonic index is medium and not extremely pronounced ([te/PW]-ratio = 0.5).

### Differential diagnosis

**Fig 11:**
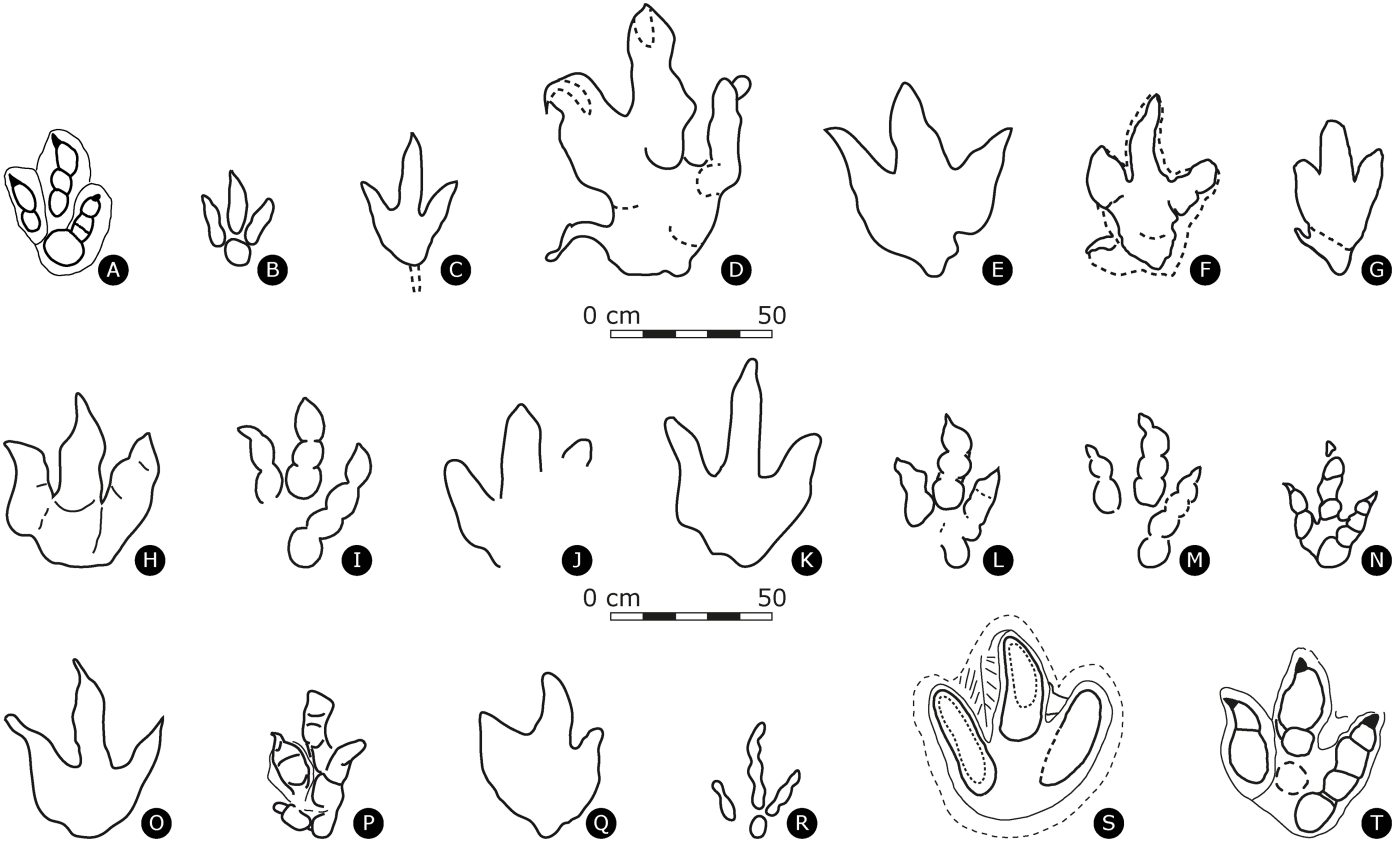
Outline drawings of selected large theropod ichnotaxa, all drawn to the same scale. Left tracks are mirrored as right tracks. **(A)** Holotype of *Megalosauripus transjurani* (TCH1030-T6-L1, MJSN-TCH006-1319). **(B)** *Asianopodus*, redrawn from [90]. **(C)** *Irenesauripus* redrawn from [86]. **(D)** *Tyrannosauripus pillmorei*, redrawn from [93]. **(E)** *Bellatoripes fredlundi*, redrawn from [94]. **(F)** *Bueckeburgichnus maximus*, redrawn from [84]. **(G)** *Euthynichnium lusitanicum*, redrawn from [3]. **(H)** *Iberosauripus grandis*, redrawn from [85]. **(I)** *Megalosauripus uzbekistanicus*, redrawn from [6]. **(J)** *Megalosauripus*-like track, redrawn from [172]. **(K)** *Megalosauripus*-like track, redrawn from [19]. **(L)** *Megalosauripus* from Arizona, redrawn from [3]. **(M)** *Megalosauripus* from Utah, redrawn from [3]. **(N)** *Megalosauripus*-like track, from [96]. **(O)** *Megalosauripus*-like track, from [108]. **(P)** *Boutakioutichnium atlasicus*, redrawn from [81]. **(Q)** Holotype of *Hispanosauropus*, redrawn from [4]. **(R)** *Megalosauropus broomensis*, redrawn from [83]. **(S)** *Megalosauripus teutonicus*, redrawn from [76]. **(T)** *Jurabrontes curtedulensis*, holotype (SCR1500-T1-L8, MJSN-SCR011-553), from [98].

Valid ichnospecies of *Megalosauripus* are *M*. *uzbekistanicus* and *M*. *teutonicus*. The major difference of *M*. *transjuranicus* with *M*. *uzbekistanicus* from the Late Jurassic of Turkmenistan and Uzbekistan [3,6,75], and *M*. *teutonicus* from the Late Jurassic (Kimmeridgian) of Northern Germany (Barkhausen tracksite; *Megalosauropus* in [76]; amended as *Megalosauripus* in [3]) lies in the size of PIV1, which is twice the width of digit IV in *M*. *transjuranicus* and is much smaller in *M*. *uzbekistanicus* (comparable to the width of digit IV) and absent in *M*. *teutonicus*. The amended diagnosis for *Megalosauripus uzbekistanicus* of [6] adds the presence of the hallux or digit I impression posteriorly and laterally oriented, which in all specimens of *M*. *transjuranicus*, independently of tracks depth, is always absent. *M*. *teutonicus* is characterized by deep tracks (10 cm), broad and short, deeply impressed digits, lack of any discrete phalangeal pad impressions and it is generally poorly preserved. All these features are clearly different from the shallow and well-defined *M*. *transjuranicus* features (Fig 11).

*Carmelopodus* tracks from the Middle Jurassic of Utah differ in the phalangeal pad formula (2:3:3 for digits II, III and IV in [77]), and in the PL: PW ratio, which characterizes *Carmelopodus* tracks as almost as long and wide.

*Kayentapus* tracks from Lower Jurassic of Arizona [78] differ in their much smaller size, the higher PL/PW-ratio index, the greater width of the interdigital angles (considering variations) and the detached digital pad PIV1 from the rest of the digit IV impression.

*Grallator* and *Eubrontes* tracks from the Early Jurassic of Connecticut and Massachusetts [79] differ in the much higher PL/PW-ratio, in the greatly developed digit III impression in *Grallator* and in the shape of the PIV1 impression in *Eubrontes*.

*Euthynichnium lusitanicum* from the Late Jurassic of Cabo Mondego, Portugal [80], amended in [3], is considered very distinctive because of the small and slender anteromedially facing hallux impression, which makes the track tetradactyl with three large, non-tapering digits with no clear phalangeal pad impressions. All these characteristics are very different from *M*. *transjuranicus*. For similar reasons the tetradactyl *Boutakioutichnium atlasicus* from the Late Jurassic of Morocco [81] is also considered different from the studied tracks.

*Megalosauropus broomensis* from the Early Cretaceous of Australia [82,83] is defined by a quite an atypical phalangeal pad formula of 3-4-5 for digits II-III-IV, which is absent in *M*. *transjuranicus*.

The taxonomic position of *Bueckeburgichnus maximus* from the Early Cretaceous of Northern Germany from the Wealden beds near Bückeburg [17], was the subject of disagreements between [84] and [5]. Although it is true that the concept of *Bueckeburgichnus maximus*, (redescribed in [84] as: “large tetradactyl theropod track with a small hallux (digit I), digit II wide and well-padded, digit III parallel sided proximally but strongly tapering distally, digit IV narrow with traces of discrete digital pads”), differs greatly from *Megalosauripus transjuranicus*, it should be underscored that the amended diagnosis of [84] is based on a different specimen with respect to the original diagnosis by [17]. In fact, the holotype of *Bueckeburgichnus maximus* is, as pointed out by [5], the same specimen on which the taxon *Megalosauripus* was coined by [14], who was referring to the drawings in fig. 4 [15] and in fig. 120 [16].

The material of *Iberosauripus grandis* from the Jurassic–Cretaceous transition of the Iberian range [85] is rather poorly preserved. Based on the illustrations and descriptions in [85], and on our own personal observations, *I*. *grandis* differs from *M*. *transjuranicus* in the width of the track, almost as wide as long (PL/PW = 1.2, table 2 in [85]), the broadness of its digit impressions, the lack of a strongly developed PIV1 impression, the general symmetric aspect with lateral digits II and IV impressions subequal in length, and a very weak mesaxonic index (0.3).

*Irenesauripus acutus* from the Aptian–Albian of Canada ([86], fig. 2, p.64) strongly differs from *M*. *transjuranicus* because of the very elongated, narrow and slender digits, larger interdigital angles, and absence of phalangeal pad impressions. This ichnotaxon is erected on a clearly compromised track due to rheological bias, which was left in a water-saturated sediment causing mud collapse and sealing of digit impressions after its formations.

*Irenichnites* [86] from the Lower Cretaceous of British Columbia is clearly different from *M*. *transjuranicus* because the heel pad is not completely developed and the track is very broad and very small, the longest measure for PL is of 15 cm.

*M*. *transjuranicus* lacks the sigmoidal digit III impression and exhibits much better-defined phalangeal pad marks with respect to *Hispanosauropus hauboldi* ([87], revised in [4] and in [88]) which is indicated as plantigrade. Although a clear and diagnostic description for this ichnogenus is not provided in these papers, it is indicated that at least some digit pad impressions are present in most examples, but they are usually not well defined [88].

*Asianopodus* from the Valaginian to Barremian of Japan [89] is diagnosed as small to medium-sized tridactyl, mesaxonic and subsymmetric track with a distinct ‘bulbous’ heel impression, and Xing et al. [90] also reported *Asianopodus* from the Early Cretaceous of China displaying a well-developed and sub-rounded metatarsophalangeal pad located axially posterior to the axis of digit III. Despite some similarity with *M*. *transjuranicus* regarding the metatarsophalangeal pad area (presence of a large PIV1), *Asianopodus* is different because of the more central position of the metatarsophalangeal pad PIV1, giving the track a symmetrical shape and because of the clear separation of PIV1 with all digit impressions. Note that [90] (fig. 6B, p. 310) figured a track as *Megalosauripus* isp., although this track is the type specimen of *Euthynichnium lusitanicum* (compare with [3], fig.7).

*Jialingpus* from the Late Jurassic of China [91,92] is similar to *M*. *transjuranicus* in respect to the size of PIV1 but differs in the overall morphology since it displays a different phalangeal formula of 2-3-3-4 respectively for digits I-II-III-IV with two developed metatarsophalangeal pads that connect with lateral digits II and IV, and the presence of a hallux (dI) impression.

Giant (PL>70 cm) theropod ichnotaxa such as *Tyrannosauripus* from the Late Cretaceous of New Mexico [93], *Bellatoripes* from the Late Cretaceous of Canada [94] and some other large to giant unnamed tracks from the Late Jurassic of Portugal [95] and Morocco [96,97] are not considered here, as they significantly differ from *Megalosauripus* and *Megalosauripus transjuranicus*. Giant (PL>50 cm) theropod tracks from Highway A16 that are significantly different from *M*. *transjuranicus* have recently been named *Jurabrontes curtedulensis* [98].

### Other *Megalosauripus*-type tracks in the tracksites

The great amount of large tridactyl tracks and trackways uncovered on six different tracksites and ten different track levels on Highway A16 allowed recognizing a wide range of morphological variations registered on the different levels and even along the course of individual trackways (especially on levels 500, 1000, 1020, 1030 and 1040). For this reason, only the best-preserved tracks were classified in the new ichnospecies *Megalosauripus transjuranicus*. Trackways that do not retain any track with sufficient diagnostic features to assign it to this new ichnospecies were addressed as: *Megalosauripus* cf. *transjuranicus*, *Megalosauripus*? *transjuranicus*, and Morphotype II *sensu* [20]. These three different morphotypes may occur on the same level and even along the course of a single trackway due to changes in substrate properties and/or kinematics of the trackmaker and this has important implications for the understanding and classification (ichnotaxonomy) of large tridactyl tracks. These aspects will further be commented in the discussion.

During the classification of the studied trackways, always the best-preserved track of a given trackway is used. A trackway with a track exhibiting diagnostic features of *M*. *transjuranicus* is classified as such even if other tracks along the trackway course do not exhibit the typical features or even do resemble Morphotype II tracks. Trackways that cannot unambiguously be classified as *M*. *transjuranicus* are classified using open nomenclature (see [99,100]) using cf. *transjuranicus* and? *transjuranicus*.

#### *Megalosauripus* cf. *transjuranicus* (Fig 12A–12H)

**Fig 12:**
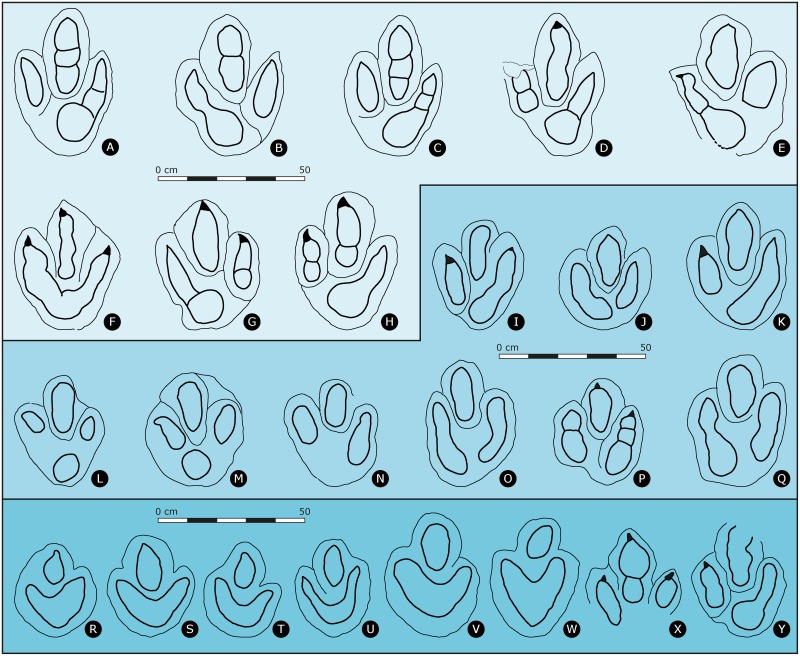
Outline drawings of *Megalosauripus* cf. *transjuranicus* (A-H), *Megalosauripus*? *transjuranicus* (I-Q), and of Morphotype II (R-Y) tracks. All tracks are drawn to the same scale. **(A)** SCR1000-T23-R1. **(B)** SCR1000-T23-L2. **(C)** SCR1000-T23-R2. **(D)** TCH1020-T1-R2. **(E)** TCH1020-T2-L1. **(F)** TCH1020-T2-R1. **(G)** TCH1020-T2-L2. **(H)** TCH1020-T2-R2. **(I)** TCH1000-TR1-R2. **(J)** TCH1000-TR1-L3. **(K)** TCH1000-TR1-R3. **(L)** TCH1000-TR2-R9. **(M)** TCH1000-TR2-L10. **(N)** TCH1000-TR2-R10. **(O)** TCH1000-TR2-L12. **(P)** TCH1000-TR2-R12. **(Q)** TCH1000-TR2-L13. **(R)** BEB500-TR7-L2. **(S)** BEB500-TR7-R2. **(T)** BEB500-TR7-R7. **(U)** BEB500-TR7-L10. **(V)** BEB500-TR7-R10. **(W)** BEB500-TR7-L11. **(X)** SCR1000-T18-R1. **(Y)** TCH1030-T1-R4.

Tracksite and levels: SCR1000, TCH1020, TCH1025, TCH1030, BSY1035. Trackways: SCR1000-T23, T24 (S6 and S7 Figs); TCH1020-T1, T2 (S20 and S21 Figs); TCH1025-T1 (S23 Fig); TCH1030-T5 (S27 Fig); BSY1035-T1, T7 (S11 Fig).

*Megalosauripus* cf. *transjuranicus* open nomenclature of the new ichnospecies is used when the track morphology is well preserved and the large and wide metatarso-phalangeal pad PIV1 is discernible and connected to digit IV, and when digits are well separated. However, not all the phalangeal pads and three claws are impressed and clearly discernible.

#### *Megalosauripus*? *transjuranicus* (Fig 12I–12Q)

Tracksites and levels: TCH1000, BSY1020, BSY1025. Trackways: TCH1000-TR1, TR2 (Fig 12I–12Q, S17 and S18 Figs); BSY1020-T1 (S9 Fig); BSY1025-T3 (S9 Fig), BEB500-TR1, TR2, TR5, TR8 (S2 Fig).

*Megalosauripus*? *transjuranicus* open nomenclature is used when tracks reflect a limited preservational variation on the strict definition of this ichnogenus [3] and therefore when digits are separated and distinguishable from one another, heel pad (PIV1) is not very discernible, and morphometric parameters for tracks and trackway configuration are typical, such as tracks longer than wide, elongated, asymmetric, moderate mesaxony, notch developed between digit II and heel area impression, trackway configuration somewhat irregular. This classification is linked to a preservational variation of the *M*. *transjuranicus* track morphology due to substrate consistency, limb kinematics and/or behavior of the trackmaker [101] rather than foot anatomy (different trackmaker).

### Other occurrences of *Megalosauripus*-type and other similar theropod tracks

Ichnotaxonomy of medium-sized to giant theropod tracks, especially of those from the Late Jurassic and Early Cretaceous, is a complicated matter and has been debated in many different papers [1–6]. Many ichnotaxa have been discussed and reviewed by various authors, but, at the moment, there is no consensus about the validity or redundancy of many of these [1–6]. The main ichnotaxa that are similar to the new ichnospecies *Megalosauripus transjuranicus* are discussed in the differential diagnosis (see section 4.2).

Lockley et al. [4] pointed out that tracks named ‘*Megalosaurus’* from the Late Jurassic of Cabo Mondego (Portugal), named *Euthynichnium lusitanicum* in [3], and problematic ichnotaxa such as *Megalosauripus* [3,5,14] and *Megalosauropus* [76] have some potential relationship to *Hispanosauropus hauboldi* [87]. Avanzini et al. [88] noted that the relationship between *Hispanosauropus* and megalosaur tracks (*Megalosauripus*) is complex. However, [102] (p.76) strongly suggested that *Hispanosauropus* should not be used anymore, and should rather be included in the *Megalosauripus* definition [3], due to the lack of a holotype and to the poor preservation of the designated topotype at the la Griega tracksite. Nevertheless, it is of no surprise that all these ichnotaxa that describe large to giant Late Jurassic theropod tracks from the Iberian Peninsula and Western Europe are morphologically similar, and to some degree also to *Megalosauripus*. In addition, some theropod tracks of comparable size from Asturias were addressed with the compound name *Megalosauripus-Kayentapus* (figured and described in [102], p.87 and fig. 9.1.7,). These tracks are clearly different from the studied material, especially because of the lack of the diagnostic large and round PIV1 connected to dIV.

In the Jura Mountains, other so far unnamed large theropod tracks occur at the Kimmeridgian La Heutte II, Grenchenberg, and Glovelier—Côte du Crêt (GLO–CCR) tracksites in NW Switzerland, and at the Tithonian Plagne tracksite in France [103].

The Glovelier tracksite was originally discovered in 1998 [104]. It consists of six tracks that are poorly preserved, lack any anatomical details such as phalangeal pads or claw marks, and that are either strongly weathered and/or they are undertracks (NLR, DM, MB pers. obs. 2016), so that they can at best be identified as tridactyl tracks of likely theropod origin, somewhat similar to Morphotype II *sensu* [20], but clearly different from *M*. *transjuranicus*.

At Grenchenberg, a trackway of a large theropod was discovered within this interval. This 3-m long trackway consists of four consecutive tracks with a mean PL of 35 cm and a slightly-curved digit III. It is similar to the giant tracks of Highway A16 [98] with a PL of nearly 80 cm [105]. From the La Heutte II tracksite, [106] described a single large theropod track that is longer than wide, has narrow interdigital angles, claw marks and pad impressions. However, based on fig. 3 in [106], the digits have no pad impressions discernible, they are not separated and they are fused in a large and rounded heel area. Based on the figure and description of [106], this track cannot be assigned to *Megalosauripus transjuranicus*, but a cast of the track is housed at the Naturmuseum Solothurn and is worth to be re-studied.

At Plagne (France), at least two trackways of medium-sized to large theropods are well preserved, but they are not yet published. Judging on personal observations (DM, 2012) both trackways labelled ‘PD’ and ‘PG’, respectively, are characterized by tracks with a PL of approximately 25–30 cm, well-separated digits with phalangeal pad impressions, and by the presence of a large PIV1 pad. These tracks can be assigned to *Megalosauripus* and maybe even *M*. *transjuranicus*, but this has to be confirmed by future studies.

The Late Jurassic Loulle tracksite in the French Jura Mountains exhibits an eight-step trackway of a giant theropod (LOU 20, mean PL = 77 cm) that was tentatively assigned to *Megalosauripus* by [107] (see their fig. 14). These authors stated that this trackway with an irregular gait exhibits asymmetric tracks with three well-separated digits, three claw marks, and a phalangeal pad configuration of 2-3-3 or -4 but that cannot be determined with confidence. Based on descriptions and fig. 14 in [107] there is no evidence for a large PIV1 in connection with dIV and for this reason, and also because of their much larger size, these tracks are not similar to *M*. *transjuranicus*. Mazin et al. [107] have also described three trackways (LOU 05, LOU 06, LOU 13) with a PL between 21–24.3 cm, which they referred to *Carmelopodus*. However, these tracks also resemble *Megalosauripus* because of their slender and well-separated digits with clear phalangeal pad impressions, but they are smaller than typical *Megalosauripus* tracks. Also, these tracks do not show evidence for a large PIV1 in connection with dIV and for this reason they cannot be assigned to *M*. *transjuranicus*.

Recently, [108] classified large theropod tracks (of up to 80 cm in pes length) from the Middle Jurassic Vale de Meios quarry from the Lusitanian basin of Portugal as *Megalosauripus* isp. Where preserved (e.g., track VMX.1 in [108], fig. 5), the phalangeal pad formula is 2-3-4 for digits II-III-IV but these *Megalosauripus* tracks do not show the diagnostic large PIV1 pad of *M*. *transjuranicus* and are for this reason different (Fig 11).

Another famous Middle Jurassic tracksite is that of Ardley Quarry (Oxfordshire, UK), which displays large tridactyl tracks indicated as *Megalosauripus*-like tracks in [19,109] and analyzed from a biomechanical and biological aspect in [110]. The best-preserved tracks such as R20 of trackway T80 ([110], fig. 6) perfectly fit this ichnogenus classification because it exhibits well-separated, slender digits with claw marks, the average TL/TW index (1.40), the inward rotation of digit III and phalangeal pads possibly with a 2-3-4 phalangeal pad configuration for dII-III-IV, the PIV1 phalangeal pad connecting to digit IV impression and the postero-medial indentation developing from the posterior margin of digit II impression. Nevertheless, Ardley Quarry tracks are larger in size and the diagnostic PIV1 is particularly small in the Ardley Quarry tracks and not as developed as in *M*. *transjuranicus*.

Other tracks assigned to *Megalosauripus* from the Late Jurassic [96] and Early Cretaceous of Morocco [111], Arizona and Utah [3,112], Poland [113], and Germany [13,18,114], and from the Middle Jurassic of Madagascar [115] and China [10] either display subequal phalangeal pads when preserved (Arizona, Utah, Germany, Morocco), lack a discrete phalangeal pad formula (Madagascar, England, Poland) or are too poorly preserved for a sound comparison (China).

To summarize, tracks from North America, Europe, North Africa and Asia that have been assigned to *Megalosauripus* differ from the new proposed ichnospecies *M*. *transjuranicus* described herein. Certainly, the most diagnostic feature of *M*. *transjuranicus* certainly is the large and rounded PIV1 pad in connection with dIV. Accordingly, this feature may become an important characteristic for future classifications of theropod ichnotaxa.

In [3], the smallest *Megalosauripus* tracks from North America, Central Asia and Europe have a PL of 39 cm, whereas the mean PL value is about 50 cm and the maximum PL 77 cm. The English tracks [19,109], and some from Uzbekistan and Turkmenistan [6], are at the upper end of the size range of *Megalosauripus* tracks. Generally, *Megalosauripus* track lengths from specimens from North America, Iberian Peninsula and Central Asia (Uzbekistan and Turkmenistan) are ranging from 40 cm to a maximum of 80 cm, while the material from Highway A16 ranges from 35.5 cm to a maximum of 45.5 cm, and thus is at the lower end for *Megalosauripus* tracks. This size range is also encompassed in *Megalosauripus* isp. from the Late Jurassic–Early Cretaceous of China ([10]: PL = 38.3 cm), Middle Jurassic of Portugal ([108]: PL = 20–80 cm) and Madagascar ([115]: average PL = 34 cm).

Although size alone should not be a criterion for ichnotaxonomic discrimination [115,116], some tracksites (Vale de Meios tracksite, [108]; Tsisandro tracksite, [115]) display some track lengths values that are outliers (smallest track recorded in Vale de Meios, Portugal is of 22 cm) of the typical *Megalosauripus* size range (PL>50 cm) at this site.

It is not unusual to observe tracks retaining similar morphologies with different sizes [10,108,117,118] because of different ontogenetic stages of the trackmakers [119], osteological convergence among different trackmakers or extramorphological factors biasing track morphology. On the other hand, it is noteworthy that among ten different stratigraphical levels analyzed, the material clearly identified as *M*. *transjuranicus* are distributed over a narrow size-range (from 35.5 cm to 42.5 cm) and have relatively small mean PL values compared to other occurrences of *Megalosauripus* tracks (see Fig 11I–11O). An explanation for this evidence might be an age-segregation distribution among these theropods [118,120], perhaps concentrating larger theropod tracks (adult-individuals) attributable to *Megalosauripus* in other areas, leaving the tidal flat as the favored environment for subadult individuals confining the size of the footprints to a narrow range [19,110]. However, that narrow PL range of *M*. *transjuranicus* indicates that the trackmaker was a medium-sized theropod, smaller than the trackmaker of other areas displaying much larger *Megalosauripus*-type tracks.

### Description of Morphotype II *sensu* [20]

Tracksites and levels: CRO500, BEB500, SCR1000, TCH1020, TCH1030, BSY1040. Trackways: CRO500-T43 (S1 Fig), BEB500-TR3, TR4, TR7 (S2 and S2 Fig), SCR1000-T18 (Fig 12X, S5 Fig), TCH1020-T3 (S22 Fig), TCH1030-T1 (Fig 12R and 12Y, S25 Fig), BSY1040-T8 (S15 Fig).

Morphotype II (Fig 12R–12Y) is characterized by subsymmetric tridactyl tracks, most of them as wide as long but also with some specimens much longer than wide ([PW/PL]-ratio ranging from 1.18–1.72) and a moderate mesaxony ([te/PW]-ratio ranging from 0.45–0.58). Digits are not well separated as in *Megalosauripus* tracks, especially the lateral digits II and IV, which are also typically merged in the heel without evidence for a postero-medial indentation below dII. Digits are tapered and rounded to slightly pointed, but no claw marks can be identified. Digit III impression is well separated from dII and dIV and blunt with a trapezoidal (clover-like) shape with the maximum width located in the medium to anterior part of the digit. It is short and forwardly directed, not sigmoidal. No phalangeal pads are discernible. All three digits are of about the same length. Tracks are usually inclined towards the digit III impression, which is the deepest one. Interdigital angles are roughly subequal. Displacement rims are often well developed, especially around digit III, sometimes they are present all around the track. There is no evidence for any manus tracks in association to the pes impressions. The [WAP/PL]-ratio ranges from 0.2 to 0.6, trackway configuration is quite variable, ranging from narrow and very straight to sinusoidal trackways displaying a ‘zig-zag’ pattern with outwardly rotated tracks and a wider gauge. Rare trackway patterns such as ‘standing still’ (pair of parallel tracks, commonly showing a small inward rotation, [121] were documented. Intra-trackway stride lengths range from 173 to 317 cm, pace lengths from 86 to 138 cm, pace angulation from 149° to 176° and speed estimations from 5.3 to 12.7 km/h.

### Description of ichnoassemblages

Large tridactyl tracks are quite a common element of the Ajoie ichnocoenosis, even though with 49 trackways they make up for only 19.4% of all documented tridactyl trackways and 10.3% of all documented dinosaur (tridactyl & sauropod) trackways.

Within both the lower and the intermediate track levels, only the lowermost main track level (i.e., level 500 and 1000, respectively) can be correlated between different sites. For instance, level SCR1000 can be correlated with TCH1000. Level CRO500 can be correlated with BEB500 and most likely also with CPP500, which, however, is located in Porrentruy about 5 km to the east of the CRO and BEB Highway A16 tracksites that are located only about 1.5 km from one another.

In most ichnoassemblages several large tridactyl trackways were documented, and they are associated with minute, small, and medium-sized theropod tracks, and with tiny, small, and medium-sized sauropod tracks. However, they are never found together with all of these different size classes of theropod and sauropod dinosaurs.

Moreover, they are never directly associated with giant (PL>50 cm) theropod and large (PL>75 cm) sauropod trackways, even though at the BSY tracksite, trackways of both giant theropods and large sauropods occur on level BSY1050 [98], only 10 cm above level BSY1040 with several trackways of *M*. *transjuranicus* including the paratype trackway. Also on BSY1040, trackway T1 clearly overprints sauropod trackway S1. This is the only case where a large tridactyl trackway overprints a sauropod trackway and therefore clearly has passed by after the sauropod.

## Discussion

### Trackmaker identification for *Megalosauripus transjuranicus*

During the Late Jurassic, the taxonomic diversity of large theropods is quite high, including the families Allosauridae, Megalosauroidea, Ceratosauria and Coelurosauria. The osteological convergence and substantial overlap in phalangeal proportions of the theropod foot would not allow a lower level distinction among different theropod taxa [122,123].

In addition, the autopodium of all these theropods are very conservative concerning the functional morphology [124–126]. Moreover, those features that could help with the identification of the trackmaker, e.g., shape, metatarsal impressions and position and orientation of digit I [127], are not preserved in the analyzed tracks. However, considering additional data such as the size and the provenance (considering both temporal and spatial distributions), a discussion on the skeletal remains of similar age as the *M*. *transjuranicus* tracks is attempted here. An identification of the large theropod trackmaker is not easy because of the scarcity of their skeletal remains in the Late Jurassic deposits of the Jura carbonate platform or surrounding areas. In the Swiss Jura Mountains, the body fossil record of theropods is scarce and only comprises an isolated allosaurid tooth from the Silberhöhle near Röschenz (Late Oxfordian, Canton Baselland; [128]); two isolated theropod teeth from the Solothurn Turtle Limestone (Late Kimmeridgian, Canton Solothurn; [129]), one of which is similar to dromaeosaurid teeth [128]; and, finally, a large (total length of about 7 cm) theropod tooth from the Moutier sauropod bone assemblage (Early Kimmeridgian, Canton Bern) initially figured in [130], and attributed to *Ceratosaurus* [131].

In the French Jura Mountains, a couple of theropod teeth are known from the Damparis sauropod assemblage [132,133] one of which is considerably large (total length of 11cm) and was attributed to *Megalosaurus insignis* by [132]. Furthermore, several isolated, huge vertebrae are known from the Oxfordian of Plaimbois-du-Miroir, Doubs Department [134], corroborating the presence of a “very large theropod of uncertain affinity” [135], but these remains still lack any closer scientific description. However, the teeth from Damparis and the remains from Plaimbois-du-Miroir are large enough to represent potential trackmakers from the Loulle quarry in the French Jura Mountains [107]. Therefore, the presence of megatheropods on the Swiss and French Jura carbonate platform during the Late Oxfordian and Kimmeridgian can now be confirmed by both the skeletal and track record.

Apart from those of the Jura Mountains, there are potential large theropod trackmakers known from the Late Jurassic in Europe and notably Portugal. These include members of the Ceratosauridae, Allosauridae, and Megalosauridae (‘megalosaur’ or ‘megalosaurid’), e.g., [2,3,84,136–139].

*Allosaurus* specimens described from the Late Jurassic of Portugal and assigned to *Allosaurus fragilis* [140,141] and *Allosaurus europaeus* [142] with an estimated hip height of 2.4 m seem plausible for having left tracks smaller than 50 cm in total length and covering the size range of *M*. *transjuranicus*. On the other hand, the largest *Allosaurus* specimens such as *Allosaurus fragilis* from the Cleveland-Lloyd Dinosaur Quarry with an estimated total body length of up to 12.5 [143,144] and notably *Saurophaganax* [145] from the Late Jurassic Morrison Formation, USA were indeed too large and probably would have left tracks much bigger (PL>50 cm) than the trackmaker of *M*. *transjuranicus*. Ceratosauridae are also known from the Late Jurassic of Portugal [146].

A juvenile allosaurid, *Sciuromimus albersdoerferi* [147], has been recently described from the Kimmeridgian of Bavaria. The estimated size for the adult animal, around 5 m in length, is compatible with the described size range for the *M*. *transjuranicus* tracks. Also *Ceratosaurus*, with a similar estimated body length [122,148] is a possible candidate for producing tracks ranging from 30 to 50 cm in pes length.

Megalosauridae or ‘megalosaurs’ are poorly understood, both in their anatomy and their phylogenetic affinities [149,150], and Thulborn [5] stated: “there exists no definite conception of megalosaurs or of their tracks”. However, *Torvosaurus* is a member of the Megalosauridae known from Colorado [151] and Portugal [95,142]. Its body length ranges from 8–12 m, which is too large for the studied tracks. Cobos et al. [85] have suggested that tracks classified as *Bueckeburgichnus*, *Hispanosauropus*, *Megalosauripus* were probably left by members of the Allosauridae. A recent paper [123] described a new large theropod, *Wiehenvenator albati*, from the Callovian of Germany, a derived megalosaurine megalosaurid closely related to *Torvosaurus*. However, an allosaurid trackmaker seems much more likely for *M*. *transjuranicus* tracks than a ‘megalosaurid’ trackmaker, which would have left larger tracks. There are larger (giant) theropod tracks discovered on the Highway A16 tracksites, i.e., *J*. *curtedulensis* [98].

### Interpretation of Morphotype II tracks

Some of the studied tracks are classified as Morphotype II *sensu* [20], characterized as subsymmetric, large, slightly mesaxonic, slightly longer than wide (sometimes almost as wide as long), with subsymmetric interdigital angles, and with blunt toes and without evidence for the impression of discrete phalangeal pads and claws. In the field, most (but not all) of these trackways were labelled as ‘TR’ trackways, as they did not show clear theropod features.

On four other different levels (1000, 1020, 1030 and 1040) from three different tracksites (BSY; TCH, SCR) Morphotype II tracks are associated with *Megalosauripus transjuranicus* (and/or cf. *transjuranicus*, and/or *Megalosauripus*? *transjuranicus*). For instance, the main track level 1000 can be correlated between the SCR and TCH tracksites; Morphotype II trackway SCR-1000-T18 is associated on the same level with the trackways *Megalosauripus* cf. *transjuranicus* SCR-1000-T23 and -T24, and with the poorly-preserved trackways *Megalosauripus*? *transjuranicus* TCH-1000-TR1 and -TR2 (S7 Fig). On level TCH1020, Morphotype II trackway TCH1020-T3 is associated with the trackways *Megalosauripus* cf. *transjuranicus* TCH1020-T1 and -T2. On level BSY1040 a Morphotype II trackway (BSY1040-T8) co-occurs with the trackways *Megalosauripus transjuranicus* BSY1040-T1, -T7 and -T9.

On level TCH1030, Morphotype II tracks even occur within trackways that can clearly be assigned to *Megalosauripus transjuranicus* such as trackways TCH1030-T2 -T6 (holotype trackway of *M*. *transjuranicus*, Fig 5), and -T7. For instance, the track TCH1030-T1-R4 clearly represents a *Megalosauripus* morphology while most other tracks of trackway TCH1030-T1 recall a Morphotype II morphology (S25 Fig). Accordingly, these Morphotype II tracks clearly are preservational variants of *Megalosauripus* tracks, related to variable substrate properties and/or trackmaker locomotion. Trackway TCH1000-TR2 (S7 Fig) is another interesting example: because of the preserved phalangeal pads and claw marks in some of the tracks, it is classified as *Megalosauripus*? *transjuranicus*, even though along the 25-m-long trackway, tracks show different morphologies, some of which strongly recall Morphotype II (e.g., TCH1000-TR2-R9, L10, R10, S7 Fig). Another example is trackway BSY1040-T8 (S15 Fig), which has the highest stride lengths (> 3 m) and speed estimation (12.7 km/h) of all studied trackways. In this trackway, tracks with altered morphology where the merging lateral digits and heel area is not discernible (more pronounced digitigrade stance due to higher locomotion speed), resemble Morphotype II tracks. Further, digit III is strongly indented into the sediment, indicating that the trackmaker was moving fast, with a high digitigrade posture causing the merging of lateral digits and the lack of a clear PIV1 impression. Accordingly, Morphotype II tracks occurring in these trackways clearly are preservation variants of *Megalosauripus* or even *M*. *transjuranicus*. All these trackways are nice examples for intra-trackway variability, where track morphology changes along the trackway course with single tracks resembling different morphotypes and even different ichnotaxa.

However, since Morphotype II tracks can, sometimes, resemble the *Therangospodus* ichnotaxon, when the two morphotypes are not co-occurring along the same trackway, it is difficult to unambiguously assess if a given Morphotype II trackway is representing the *Therangospodus* ichnotaxon or if it is a preservation variant of *Megalosauripus*. Lockley et al. [136] described *T*. *pandemicus* as medium-sized, elongated, asymmetric tracks (therefore longer than wide and not as long as wide), having digital pad impressions without creases separating discrete phalangeal pads, but which, when appreciable, suggest a 2-3-4 phalangeal pad formula; claw marks sometimes preserved; and a trackway configuration quite similar to *Megalosauripus* (narrow trackway, variable step lengths and a high 170° pace angulation). In other words, the diagnosis of *Therangospodus* [21,22,152,153] is entirely based on the lack of those features that are diagnostic for *Megalosauripus*, i.e., oval digital pads not separated into discrete phalangeal pads, no rotation of digit III, no separation on the proximal margins of the digits by a hypex.

Gierliński et al. [154] (p. 445) reported a tridactyl track from the Toarcian of Poland with more distinct phalangeal pads, but these authors stated that they were "not sure if *Therangospodus* should be distinguished from *Megalosauripus*". A very important consideration in [154] is that they have noticed that "diagnostic features separating them (= *Therangospodus* vs. *Megalosauripus*) are entirely extramorphological and subject to growth and behavioral changes or potentially influenced by the substrate nature, so they may not reflect real taxonomic differences". Piñuela [102] also pointed out that because of the resemblance of *Therangospodus pandemicus* with altered and poorly-preserved specimens of *Megalosauripus*, this might at least in some cases be indicative, that *Therangospodus pandemicus* is the product of a morphological variation of the *Megalosauripus* ichnogenus. This would favor the interpretation of some of the tracks included in Morphotype II trackways as preservation variants of *Megalosauripus transjuranicus* or *Megalosauripus* in general, rather than assigning them to *Therangospodus pandemicus*. On the other hand, [6], although noticing that some of the weathered *Megalosauripus* tracks are similar in overall morphology to *Therangospodus*, but larger in size, concluded that tracks preserved at the Khodja-Pil-Ata site (Turkmenistan) represent the two distinct and valid ichnotaxa *Megalosauripus* and *Therangospodus*.

The theropod-like Morphotype II tracks are, however, identified as variants of *Megalosauripus* rather than *Therangospodus*. Nevertheless, as the distinction between the two ichnotaxa is very weak and preservation-dependent, further investigations is needed in order to clearly trace this differentiation. However, there are some Morphotype II tracks that consistently show a morphology different from the ‘classical’ theropod one. On level 500 (the lowermost track level) at the CRO and BEB tracksites, Morphotype II tracks systematically occur along several trackways (CRO500-T43, BEB500-TR3, -TR4, -TR7, S1–S3 Figs) without their morphology being susceptible to a noticeable degree of intra-trackway variability. Some of the trackways of BEB500 are very long and exhibit more than 40 tracks per trackway consistently exhibiting a Morphotype II morphology. These are not the only large tridactyl tracks preserved on level BEB500: some trackways (BEB500-TR1, -TR2, -TR5, -TR8) also contain tracks that can be attributed to *Megalosauripus* isp., and on level CPP500 some *Megalosauripus*? *transjuranicus*, but no Morphotype II tracks occurred.

Accordingly, the interpretation of the large tridactyl trackways of level 500 is not straightforward and unambiguous. The long trackways consistently exhibiting a Morphotype II track morphology were most likely left by a different trackmaker from that of the *Megalosauripus* trackways, as it is much less likely, although not impossible, to systematically encounter a preservation variant with a well-defined and consistent morphology that is so different from the ‘normal’ (*Megalosauripus*) track morphology.

In the case where some of the tracks along a trackway were attributed to *Megalosauripus* isp., the rest of Morphotype II tracks are likely to be preservational variants of this ichnotaxon, which presence is confirmed in level 500 (i.e., CPP500-T1). However, it is worth considering a second scenario in which the more theropodian tracks are the preservation variants of the less detailed and more abundant Morphotype II tracks. Anyhow, considering the entire ichnoassociation, this latter scenario is less likely than the former one. The Morphotype II trackways of level BEB500 that contain some tracks attributed to *Megalosauripus* isp. were left by a ‘*Megalosauripus*-trackmaker’.

To summarize, it is concluded that the trackways of level BEB500 consistently exhibiting Morphotype II morphologies that are not preservational variants of *Megalosauripus* isp. or *M*. *transjuranicus*, and that they belong to a different trackmaker, possibly an ornithopod dinosaur. Hence, the present evidence also indicates that (at least) on level 500, trackways consistently exhibiting Morphotype II morphology associated with *Megalosauripus*-type trackways, imply the presence of two different large tridactyl trackmakers.

For level CRO500, and specifically for the Morphotype II trackway CRO500-T43 (S1 Fig), Marty [20] stated that this) shares both ornithopod and theropod characteristics, that it clearly differs from *Megalosauripus* (*sensu* [3]), and that it may have been left by a trackmaker with a well-padded, fleshy foot. Similarities with the ichnotaxon *Therangospodus* were noted but the tracks cannot be unambiguously assigned, as this ichnotaxon is, at present, not clearly defined and it would need a revision before being considered.

The trackways BEB500-TR5, TR7, TR8 and CRO500-T43 consistently exhibit Morphotype II tracks that systematically lack most of morphological features (e.g., phalangeal pads, claw marks). However, the absence of a feature does not necessarily imply that the trackmaker's foot anatomically lacks this feature (i.e., phalangeal pads, claw marks), as the absence of a feature could also be related to substrate properties, kinematics, and/or taphonomical and preservational reasons (see also [54]). Considering these possible preservational issues, CRO500-T43 and the BEB500 Morphotype II trackways displaying a consistent morphology without marked intra-trackway variability indicate that they were left by a different trackmaker than the *Megalosauripus transjuranicus* trackways, or in other words, these trackways are not poorly-preserved *Megalosauripus* trackways.

### Trackmaker identification for purported ornithopod (Morphotype II) tracks

Given the peculiar features of Morphotype II tracks of BEB500 and CRO500 levels, and despite that a trackmaker cannot unambiguously be identified, the hypothesis of an ornithopod trackmaker is here supported. Accordingly, this would be the first evidence for the possible presence of ornithopod dinosaurs on the Jura carbonate platform.

The presence of a medium-sized to large sized ornithopod trackmaker during the Late Jurassic (Kimmeridgian-Tithonian) is supported by the skeletal record of the Iberian Peninsula [155–159], England [160] and North America (Morrison Formation, [161,162]) that indicate the presence of both Ankylopollexia and Dryomorpha for non-Iguanodontoidea ornithopod. While skeletal remains of the hypsilophodontid *Othnielia* from the Kimmeridgian-Tithonian are quite abundant in the Morrison Formation [161], Late Jurassic records of hypsilophodontid ornithopods are scarce and few osteological remains were found in Portugal [155,156]. Anyhow, the high degree of morphological convergence of the foot osteology between medium-sized theropods and ornithopods from the Late Jurassic complicates a clear trackmaker assignment [116,152,163].

Even with a lot of available material, the present case of the Morphotype II trackways shows the difficulty to distinguish between poorly-preserved theropod tracks and (poorly-preserved) tracks that were likely left by ornithopods.

### Paleoecological and paleo(bio)geographical implications

The frequency of *Megalosauripus transjuranicus* trackways in the Ajoie ichnocoenosis indicates that large theropods were commonly present in tidal-flat environments of the Jura carbonate platform and represent a quite common and typical element of this ichnocoenosis. Within the Ajoie ichnocoenosis, *Megalosauripus* is associated with tiny, small, and medium-sized sauropod, and minute, small, and medium-sized theropod tracks. Giant (i.e., PL > 50 cm) theropod tracks, i.e. *Jurabrontes curtedulensis*, are also present but much rarer and, when preserved, they never co-occur on the same level nor the same ichnoassemblages *M*. *transjuranicus* [98]. On several ichnoassemblages, *Megalosauripus transjuranicus* trackways head in similar directions as sauropod trackways and at least one trackway overprints (‘follows’) a small sauropod trackway. Consequently, it can be assumed that in an open, flat and easily-overviewed tidal-flat paleoenvironment with harsh or no vegetation cover, sauropods, and notably the smaller animals, were exposed to a severe predation hazard.

As most other known occurrences of *Megalosauripus* tracks, *M*. *transjuranicus* is found in coastal tidal-flat deposits, likely reflecting the preference of the trackmakers for broad, flat areas, with abundance of food (other dinosaurs, fishes, invertebrates) and good hunting possibilities (as also seen in [108]).

Level 500 is the only level where Morphotype II tracks are tentatively assigned to an ornithopod trackmaker. The possible presence of ornithopods has important implications for the interpretation of the dinosaur community on the Jura carbonate platform. Apart from one track produced by a large ornithopod from the Late Jurassic of Portugal [158], [102] and [163] have recently reported four parallel trackways of medium-sized and robust ornithopods from the Late Jurassic of Asturias (Spain), which constitute the first ornithopod trackways known from the Late Jurassic of Europe (the one described in [9] is a theropod track; see also [18]). This fact reinforces the possibility of having both large theropods and ornithopods trackways in the Ajoie ichnocoenosis.

The widespread and rich dinosaur track record of the Jura Mountains indicates that large parts of the Jura carbonate platform were emergent during several and prolonged time periods allowing the development of a soil [164] and vegetation cover [165–168], freshwater sources [46], and *in situ* dinosaur populations [20,169]. This is further supported by the frequency of large theropod tracks and points to a ‘faunal exchange corridor’ for the exchange (on geological time spans) of dinosaur faunas between further south (Iberian Massif–Massif Central) and further north (Rhenish Massif–London-Brabant Massif) [20,48,49]. Skeletal remains of *Allosaurus fragilis* [140] and *Stegosaurus* [170] indicate land bridges over the North Atlantic [171] and via Portugal, because of dinosaur remains with Morrison Formation affinity [142,146,156]. Such faunal exchanges are supported by the presence of large theropod tracks (with a similar morphology) in the Late Jurassic of France (*Megalosauripus*), N Germany (*Megalosauripus*), Morocco, Portugal (*Euthynichnium*), and Uzbekistan (*Megalosauripus*).

## Conclusions and outlook

- Based on very well-preserved and rich material including trackways with several well-preserved tracks exhibiting substantial anatomical details, *Megalosauripus transjuranicus*, a new ichnospecies of a large theropod dinosaur is erected and described in detail.
- *M*. *transjuranicus* is easily differentiated from previously-named ichnotaxa by the presence of a pronounced, large and well-rounded proximal pad on dIV. This feature does not occur in any of the many minute, small, and medium-sized tridactyl trackways documented on Highway A16 tracksites.
- All trackways assigned to *M*. *transjuranicus* fall into a narrow size range with a mean PL ranging from 35.5 to 42.5 cm. This indicates a large predator as trackmaker, but by far not the largest theropod known from the Late Jurassic.
- An allosaurid theropod is considered as the most likely trackmaker for *Megalosauripus transjuranicus*, although Megalosaurids might also be possible candidates.
- Most of Morphotype II trackways (including all from the intermediate track levels) are preservational variants of *Megalosauripus* trackways, as indicated by trackways exhibiting both morphotypes along their course.
- Trackway CRO500-T43 and several considerably long trackways on level BEB500 systematically show Morphotype II tracks without any evidence for typical *Megalosauripus* features (such as phalangeal pads). These trackways are tentatively interpreted as produced by an ornithopod trackmaker and therefore, this would be the first evidence for ornithopod dinosaurs on the Jura carbonate platform.
- Trackways of possible large ornithopods and large theropods co-occur on level 500, although not in the same ichnoassemblage (site). Nonetheless, this indicates the coeval presence of large carnivorous theropod and herbivorous ornithopod dinosaurs on the Jura carbonate platform.
- The studied material shows that Morphotype II tracks could represent at least two different trackmakers (ornithopod and theropod), and that poorly-preserved *Megalosauripus* tracks, often, cannot be clearly distinguished and may be confounded with each other.
- The frequent presence of *Megalosauripus transjuranicus* trackways in the Ajoie ichnocoenosis indicates that large theropods were common in tidal-flat environments of the Jura carbonate platform.
- Within the Ajoie ichnocoenosis, *Megalosauripus transjuranicus* is associated with tiny, small, and medium-sized sauropod, and minute, small, and medium-sized theropod tracks.
- Within the Ajoie ichnocoenosis giant (i.e., PL> 50 cm) theropod tracks are rare and never co-occur with *M*. *transjuranicus* on the same level.
- *Megalosauripus transjuranicus* trackways generally head in similar directions as sauropod trackways, and at least one trackway (BSY1040-T1) overprints (‘follows’) a small sauropod trackway.
- During the Late Jurassic, the Jura carbonate platform may have represented a ‘migration corridor’ for the exchange (on geological time spans) of dinosaur faunas between further south (Iberian Massif–Massif Central) and further north.

## Supporting information

S1 Text Description and interpretation of tracks and trackways. (DOC)

S1 Fig CRO500-T43. Outline drawing of the trackway (scale 1:50).
(TIF)

S2 Fig BEB500. Outline drawings of trackways from BEB500 (scale 1:50). **(A)** BEB500-TR1. **(B)** BEB500-TR2. **(C)** BEB500-TR3. **(D)** BEB500-TR4. **(E)** BEB500-TR5. **(F)** BEB500-TR8.
(TIF)

S3 Fig BEB500-TR7. **(A)** Outline drawing of the trackway (scale 1:50). **(B)** Photo of BEB500-TR7-L2. Scale bar 20 cm. **(C)** Interpretative outline drawing of BEB500-TR7-L2. **(D)** False-color depth map of BEB500-TR7-L2. Depth measured in mm. **(E)** Photo of BEB500-TR7-R2. Scale 20 cm. **(F)** Interpretative outline drawing of BEB500-TR7-R2. **(G)** False-color depth map of BEB500-TR7-R2. Depth measured in mm. **(H)** False-color depth map of BEB500-TR7-R5 obtained from laserscanner. Depth measured in mm. **(I)** Photo of BEB500-TR7-R7. Scale 20 cm. **(J)** Interpretative outline drawing of BEB500-TR7-R7. **(K)** False-color depth map of BEB500-TR7-R7. Depth measured in mm. **(L)** Photo of BEB500-TR7-L10. Scale 20 cm. **(M)** Interpretative outline drawing of BEB500-TR7-L10. **(N)** False-color depth map of BEB500-TR7-L10. Depth measured in mm. **(L)** Photo of BEB500-TR7-R10. Scale 20 cm. **(P)** Interpretative outline drawing of BEB500-TR7-R10. **(Q)** False-color depth map of BEB500-TR7-R10. Depth measured in mm. **(R)** Photo of BEB500-TR7-L11. Scale 20 cm. **(S)** Interpretative outline drawing of BEB500-TR7-L11. **(T)** False-color depth map of BEB500-TR7-L11. Depth measured in mm.
(TIF)

S4 Fig CPP500-T1. Outline drawing of the trackway (scale 1:50).
(TIF)

S5 Fig SCR1000-T18. **(A)** Outline drawing of the trackway (scale 1:50). **(B)** Photo of SCR1000-T18-R1. Scale bar 20 cm. **(C)** Interpretative outline drawing of SCR1000-T18-R1. **(D)** False-color depth map of SCR1000-T18-R1. Depth measured in mm.
(TIF)

S6 Fig SCR1000-T23. **(A)** Outline drawing of the trackway (scale 1:50). **(B)** Photo of SCR1000-T23-R1. Scale bar 30 cm. **(C)** Interpretative outline drawing of SCR1000-T23-R1. **(D)** False-color depth map of SCR1000-T23-R1. Depth measured in mm. **(E)** Photo of SCR1000-T23-L2. Scale 30 cm. **(F)** Interpretative outline drawing of SCR1000-T23-L2. **(G)** False-color depth map of SCR1000-T23-L1. Depth measured in mm. **(H)** Photo of SCR1000-T23-R2. Scale 30 cm. **(I)** Interpretative outline drawing of SCR1000-T23-R2. **(K)** False-color depth map of SCR1000-T23-R2. Depth measured in mm.
(TIF)

S7 Fig Trackways from levels SCR1000 and TCH1000. Outline drawings at 1:50 scale of trackways from SCR1000 (A-B) and TCH1000 (C-D). **(A)** SCR1000-T23. **(B)** SCR1000-T24. **(C)** TCH1000-TR1. **(D)** TCH1000-TR2.
(TIF)

S8 Fig Trackways from levels BSY1000, BSY1005, BSY1010, and BSY 1040. Outline drawings at 1:50 scale of trackways from different levels of BSY. **(A)** BSY1005-T1. **(B)** BSY1010-T1. **(C)** BSY1015-T1. **(D)** BSY1040-T7.
(TIF)

S9 Fig Trackways from levels BSY1020, and BSY1025. Outline drawings at 1:50 scale of trackways from different levels of BSY. **(A)** BSY1020-T1. **(B)** BSY1025-T3.
(TIF)

S10 Fig Trackways from level BSY1025. Outline drawings at 1:50 scale of trackways from BSY1025. **(A)** BSY1025-T1. **(B)** BSY1025-T2.
(TIF)

S11 Fig Trackways from level BSY1035. Outline drawings at 1:50 scale of trackways from BSY1035. **(A)** BSY1035-T1. **(B)** BSY1035-T7.
(TIF)

S12 Fig Trackways from level BSY1025. Outline drawings at 1:50 scale of trackways from BSY1035. **(A)** BSY1035-T2. **(B)** BSY1035-T5. **(C)** BSY1035-T3. **(D)** BSY1035-T4. **(E)** BSY1035-T8.
(TIF)

S13 Fig BSY1035-T6-L2 (paratype). **(A)** Outline drawing at 1:50 scale. **(B)** Photo. Scale 30 cm. **(C)** Interpretative outline drawing. **(D)** False-color depth map. Depth measured in mm.
(TIF)

S14 Fig BSY1040-T1. **(A)** Outline drawing at 1:50 scale of the trackway. **(B)** Photo of BSY1040-T1-R1 (paratype). Scale bar 20 cm. **(C)** Interpretative outline drawing of BSY1040-T1-R1. **(D)** False-color depth map of BSY1040-T1-R1. Depth measured in mm. **(E)** Photo of BSY1040-T1-L2. Scale bar 20 cm. **(F)** Interpretative outline drawing of BSY1040-T1-L2. **(G)** False-color depth map of BSY1040-T1-L2. Depth measured in mm. **(H)** Photo of BSY1040-T1-R2. Scale bar 20 cm. **(I)** Interpretative outline drawing of BSY1040-T1-R2. **(J)** False-color depth map of BSY1040-T1-R2. Depth measured in mm. **(K)** Photo of BSY1040-T1-L3. Scale bar 20 cm. **(L)** Interpretative outline drawing of BSY1040-T1-L3. **(M)** False-color depth map of BSY1040-T1-L3. Depth measured in mm.
(TIF)

S15 Fig BSY1040-T8. Outline drawing at 1:50 scale of the trackway.
(TIF)

S16 Fig BSY1040-T9. **(A)** Outline drawing at 1:50 scale of the trackway. **(B)** Photo of BSY1040-T9-R3. Scale bar 30 cm. **(C)** Interpretative outline drawing of BSY1040-T9-R3. **(D)** False-color depth map of BSY1040-T9-R3. Depth measured in mm.
(TIF)

S17 Fig TCH1000-TR1. **(A)** Outline drawing at 1:50 scale of the trackway. **(B)** Photo of TCH1000-TR1-R2. Scale bar 30 cm. **(C)** Interpretative outline drawing of TCH1000-TR1-R2. **(D)** False-color depth map of TCH1000-TR1-R2. Depth measured in mm. **(E)** Photo of TCH1000-TR1-L3. Scale bar 30 cm. **(F)** Interpretative outline drawing of TCH1000-TR1-L3. **(G)** False-color depth map of TCH1000-TR1-L3. Depth measured in mm. **(H)** Photo of TCH1000-TR1-R3. Scale bar 30 cm. **(I)** Interpretative outline drawing of TCH1000-TR1-R3. **(J)** False-color depth map of TCH1000-TR1-R3. Depth measured in mm.
(TIF)

S18 Fig TCH1000-TR2. **(A)** Outline drawing at 1:50 scale of the trackway. **(B)** Photo of TCH1000-TR1-R9. Scale bar 30 cm. **(C)** Interpretative outline drawing of TCH1000-TR1-R9. **(D)** False-color depth map of TCH1000-TR1-R9. Depth measured in mm. **(E)** Photo of TCH1000-TR1-L10. Scale bar 30 cm. **(F)** Interpretative outline drawing of TCH1000-TR1-L10. **(G)** False-color depth map of TCH1000-TR1-L10. Depth measured in mm. **(H)** Photo of TCH1000-TR1-R10. Scale bar 30 cm. **(I)** Interpretative outline drawing of TCH1000-TR1-R10. **(J)** False-color depth map of TCH1000-TR1-R10. Depth measured in mm. **(K)** Photo of TCH1000-TR1-L12. Scale bar 30 cm. **(L)** Interpretative outline drawing of TCH1000-TR1-L12. **(M)** False-color depth map of TCH1000-TR1-L12. Depth measured in mm. **(N)** Photo of TCH1000-TR1-R12. Scale 30 cm. **(O)** interpretative outline drawing of TCH1000-TR1-R12. **(P)** False-color depth map of TCH1000-TR1-R12. Depth measured in mm. **(Q)** Photo of TCH1000-TR1-L13. Scale bar 30 cm. **(R)** Interpretative outline drawing of TCH1000-TR1-L13. **(S)** False-color depth map of TCH1000-TR1-L13. Depth measured in mm.
(TIF)

S19 Fig TCH1015-T1. **(A)** Outline drawing at 1:50 scale of the trackway. **(B)** Photo of TCH1015-T1-L2. Scale bar 30 cm. **(C)** Interpretative outline drawing of TCH1015-T1-L2. **(D)** False-color depth map of TCH1015-T1-L2. Depth measured in mm. **(E)** Photo of TCH1015-T1-R3. Scale 20 cm. **(F)** Interpretative outline drawing of TCH1015-T1-R3. **(G)** False-color depth map of TCH1015-T1-R3. Depth measured in mm.
(TIF)

S20 Fig TCH1020-T1. **(A)** Outline drawing at 1:50 scale of the trackway. **(B)** Photo of TCH1020-T1-R2. Scale bar 30 cm. **(C)** Interpretative outline drawing of TCH1020-T1-R2. **(D)** False-color depth map of TCH1020-T1-R2. Depth measured in mm.
(TIF)

S21 Fig TCH1020-T2. **(A)** Outline drawing at 1:50 scale of the trackway. **(B)** Photo of TCH1020-T2-L1. Scale bar 18 cm (10 cm for the black/white scale bar). **(C)** Interpretative outline drawing of TCH1020-T2-L1. **(D)** False-color depth map of TCH1020-T2-L1. Depth measured in mm. **(E)** Photo of TCH1020-T2-R1. Scale bar 20 cm. **(F)** Interpretative outline drawing of TCH1020-T2-R1. **(G)** False-color depth map of TCH1020-T2-R1. Depth measured in mm. **(H)** Photo of TCH1020-T2-R2. Scale bar 18 cm (10 cm for the black/white scale bar). **(I)** Interpretative outline drawing of TCH1020-T2-R2. **(J)** False-color depth map of TCH1020-T2-R2. Depth measured in mm.
(TIF)

S22 Fig TCH1020-T3. Outline drawing at 1:50 scale of the trackway.
(TIF)

S23 Fig TCH1025-T1. **(A)** Outline drawing at 1:50 scale of the trackway. **(B)** Photo of TCH1025-T1-L4. Scale bar 20 cm. **(C)** Interpretative outline drawing of TCH1025-T2-L1. **(D)** False-color depth map of TCH1025-T1-L4. Depth measured in mm.
(TIF)

S24 Fig TCH1025-T2. **(A)** Outline drawing at 1:50 scale of the trackway. **(B)** Photo of TCH1025-T2-L1 (paratype). Scale bar 20 cm. **(C)** Interpretative outline drawing of TCH1025-T2-L1. **(D)** False-color depth map of TCH1025-T2-L1 Depth measured in mm.
(TIF)

S25 Fig TCH1030-T1. **(A)** Outline drawing at 1:50 scale of the trackway. **(B)** Photo of TCH1030-T1-R4. Scale bar 20 cm. **(C)** Interpretative outline drawing of TCH1030-T1-R4. **(D)** False-color depth map of TCH1030-T1-R4. Depth measured in mm.
(TIF)

S26 Fig TCH1030-T2. **(A)** Outline drawing at 1:50 scale of the trackway. **(B)** Photo of TCH1030-T2-R2 (paratype). Scale bar 30 cm. **(C)** interpretative outline drawing of TCH1030-T2-R2. **(D)** False-color depth map of TCH1030-T2-R2. Depth measured in mm. **(E)** Photo of TCH1030-T2-L3 (paratype). Scale bar 30 cm. **(F)** Interpretative outline drawing of TCH1030-T2-L3. **(G)** False-color depth map of TCH1030-T2-L3. Depth measured in mm. **(H)** Photo of TCH1030-T2-R3. Scale bar 30 cm. **(I)** Interpretative outline drawing of TCH1030-T2-R3. **(J)** False-color depth map of TCH1030-T2-R3. Depth measured in mm.
(TIF)

S27 Fig Trackways from level TCH1030. **(A)** Outline drawing at 1:50 scale of TCH1030-T3. **(B)** Photo of TCH1030-T3-L1. Scale bar 30 cm. **(C)** Interpretative outline drawing of TCH1030-T3-L1. **(D)** False-color depth map of TCH1030-T3-L1. Depth measured in mm. **(E)** Outline drawing of TCH1030-T3 (scale 1:50).
(TIF)

S28 Fig TCH1020-T3. Outline drawing at 1:50 scale of the trackway.
(TIF)

S29 Fig TCH1030-T6. **(A)** Outline drawing at 1:50 scale of the trackway. **(B)** Photo of TCH1030-T6-L2 (holotype). Scale bar 30 cm. **(C)** Interpretative outline drawing of TCH1030-T6-L2. **(D)** False-color depth map of TCH1030-T6-L2. Depth measured in mm.
(TIF)

S30 Fig TCH1030-T7. **(A)** Outline drawing at 1:50 scale of the trackway. **(B)** Photo of TCH1030-T7-L2 (paratype). Scale bar 30 cm. **(C)** interpretative outline drawing of TCH1030-T7-L2. **(D)** False-color depth map of TCH1030-T7-L2. Depth measured in mm.
(TIF)

S1 Table Measurement tables. **(A)**Measurements made on material in the collection. **(B)** Measurements taken in the field. **(C)** Averages calculated from the field data. **(D)**Standard deviations for the field data.
(XLSX)

## Abbreviations

MJSN: JURASSICA Muséum (Musée jurassien des sciences naturelles), Porrentruy, Canton Jura, Switzerland
NMS: Naturmuseum Solothurn
PALA16: Paléontologie A16, Section d’archéologie et paléontologie, Office de la culture, Porrentruy, Canton Jura, Switzerland
