# Supplementary material for: Megalosauripus transjuranicus ichnosp. nov. A new Late Jurassic theropod ichnotaxon from NW Switzerland and implications for tridactyl dinosaur ichnology and ichnotaxomy
Source: PLoS One. 2017 Jul 17;12(7):e0180289. doi: 10.1371/journal.pone.0180289 (PMC5513421; doi:10.1371/journal.pone.0180289)
Supplement: S1 Text — (DOC) [file pone.0180289.s001.doc]

**S1. Description and interpretation of tracks and trackways**

Generally, the quality of the tracks varies a lot, but all the key specimens are amongst the best-preserved ones (preservation quality > 2.5 *sensu* [60]). A specific preservation value *sensu* [60] is indicated for the best-preserved tracks in the detailed descriptions below.

In the following descriptions, the studied tracks are identified and assigned to:

(1) *Megalosauripus transjuranicus* (see Systematic paleontology).

(2) *Megalosauripus* cf. *transjuranicus* in open nomenclature when metatarso-phalangeal pad PIV1 is appreciable, large and connected to digit IV, digits are well separated, at least one phalangeal pad per digit is discernible, and claw marks are preserved.

(3) *Megalosauripus* *?transjuranicus* in open nomenclature when digits are separated, heel pad not very well discernable, and morphometric parameters for tracks and trackway configuration typical for this ichnotaxon accordingly to the definition of [3].

(4) Morphotype II *sensu* [20] (p.135) when tracks are subsymmetric, almost as wide as long, digits are not well separated, phalangeal pads are absent, digits II and IV are merged in the heel.

Please note that the described trackways are illustrated in the Supporting Information files S2 to S31 and that all track and trackway parameter data can be found in the Supporting Information file S32.

**Chevenez—Combe Ronde (CHE–CRO) tracksite**

**Level 500**

**Trackway CRO500-T43 (S2)**

Description: Five-tracks continuous trackway located in the northeasternmost part of excavation area 2, crossing three sauropod trackways. The total length of the trackway is of 4.4 m, [WAP/PL]-ratio is 0.2 indicating a very narrow gauge. The mean rotation of the tracks is low, slightly outward (+5°) for the left and slightly inward (-5°) for the right pes tracks. The mean PaL is 96.5 cm for the left-right pace and 112.0 cm for the right-left pace, giving the trackway a slightly irregular configuration. Average SL is 207.3 cm, PA is 171º and speed estimation is 7.7 km/h. Mean PL is 30 cm and PW 27 cm. Identification of left and right tracks was not so obvious, because the trackway is indeed narrow, and because the tracks are subsymmetric in shape. The quality of the tracks falls in grade 1 [60]. Tracks are almost as long as wide and lack a differentiated phalangeal pad configuration. Lateral and medial digits II and IV are connected in the heel area. DIII is separated from dII and dIV and has a clover-like shape.

Interpretation: Trackway configuration is reflecting a slight variability in the PaL and in the alternating pes rotation, which together with the very low [WAP/PL]-ratio suggest a trackmaker with a very narrow posture, which is not related to a faster gait, as the speed estimation is within the walking range.

**Courtedoux—Béchat Bovais tracksite (CTD–BEB)**

**Level 500**

**Trackway BEB500-TR1 (S3)**

Description: Fifteen-tracks discontinuous, 18.5 m long trackway located in excavation area 1. It crosses eleven small tridactyl trackways and two sauropod trackways. It has a very narrow gauge as expressed by a [WAP/PL]-ratio of 0.2. Average PaL is 118.4 cm for the left-right pace and 114.1 cm for the right-left pace. Average SL is 230.5 cm, PA is 173º and speed estimation is 7.8 km/h. Mean PL is 34.3 cm and PW 30.1 cm. Quality of the tracks is of grade 2. Phalangeal pads are present in L2; claw marks occur in L3 and L4.

Identification of left and right tracks was not so obvious, because the trackway is narrow, and because the tracks are subsymmetric in shape. Tracks are almost as long as wide and lack a differentiated phalangeal pad configuration. Lateral and medial digits II and IV are connected in the heel area.

**Trackway BEB500-TR2 (S3)**

Description: Eighteen-tracks continuous trackway located in excavation area 1 with a total length of 17.9 m. It crosses 18 smaller tridactyl trackways, two of which are subparallel to the studied trackway but with opposite direction, and three sauropod trackways. It has a comparatively wider gauge as expressed by a [WAP/PL]-ratio of 0.6. Average PaL is 106 cm for the left-right pace and 110 cm for the right-left pace. Average SL is 196 cm, PA is 149º and speed estimation is 6.4 km/h. Mean PL is 34 cm and PW 30.6 cm. Quality of the tracks is of grade 2. Tracks are almost as long as wide, with a very weak mesaxony. Phalangeal pads are present in R5; claw marks occur in R2, L5, R6, and L6. Trackway configuration is very irregular, with a marked sinusoidal and intermittent aspect caused by differences in pace lengths. This is especially visible in tracks L7 and R7, which are located besides each other and are here considered as ‘standing-still’ tracks. This rare record of ‘standing still’ tracks is expressed by a pair of parallel tracks, both showing a small inward rotation. Moreover, a decrease/increase in pace lengths is visible before and after the standing tracks, indicating that the trackmaker was decelerating before and accelerating after stopping.

**Trackway BEB500-TR3 (S3)**

Description: Fifteen-tracks continuous 14.5 m long trackway, located in excavation area 1. It crosses one sauropod trackway and twelve small tridactyl trackways. The gauge is very narrow as expressed by a [WAP/PL]-ratio of 0.3. Average PaL is 123 cm for the left-right pace and 124 cm for the right-left pace. Average SL is 242 cm, PA is 167º and speed estimation is 9 km/h. Mean PL is 32.6 cm and PW 28.5 cm. Quality of the tracks is of grade 2. Identification of left and right tracks is not so obvious, because the trackway is narrow, tracks are sometimes alternatively outwardly and inwardly oriented, and because the tracks are subsymmetric in shape. Tracks are almost as long as wide and lack a differentiated phalangeal pad configuration. Lateral and medial digits II and IV are connected in the heel area.

**Trackway BEB500-TR4 (S3)**

Description: Eleven-tracks discontinuous trackway of 14.5 m length, located in excavation area 1. It crosses one sauropod trackway and twenty small tridactyl trackways. It has a very narrow gauge as expressed by a [WAP/PL]-ratio of 0.4. Average PaL is 95 cm for the left-right pace and 102 cm for the right-left pace. Average SL is 198 cm, PA is 160º and speed estimation is 5.3 km/h. Track length is 38.4 cm and track width is 27.7 cm. Quality of the tracks is of grade 1.5. The trackway is quite irregular due to the lack of some tracks. Tracks are almost as long as wide and lack a differentiated phalangeal pad configuration. Lateral and medial digits II and IV are connected in the heel area.

**Trackway BEB500-TR5 (S3)**

Description: 40-tracks discontinuous trackway, 34 m long, located in excavation area 1. It crosses 30 small tridactyl trackways and three sauropod trackways. It has a very narrow gauge as expressed by a [WAP/PL]-ratio of 0.4. Average PaL is 93.1 cm for the left-right pace and 94 cm for the right-left pace. Average SL is 181.9 cm, PA is 160º and speed estimation is 5.3 km/h. Mean PL is 29.6 cm and PW 25.2 cm. Tracks are almost as long as wide and lack a differentiated phalangeal pad configuration. Quality of the tracks is of grade 1.5. Tracks are very poorly preserved throughout the trackway, which has a marked sinusoidal configuration. However, phalangeal pads can be recognized in R1 and R11. Lateral and medial digit impressions II and IV connect, forming a (sub)rounded heel.

**Trackway BEB500-TR7 (S4)**

Description: 35-tracks discontinuous, 42.8 m long trackway located in excavation area 1. It crosses seventeen small tridactyl trackways, six sauropod trackways and it is parallel to two other small tridactyl trackways. It has a narrow gauge as expressed by a [WAP/PL]-ratio of 0.5. Average PaL is 127 cm for the left-right pace and 126 cm for the right-left pace. Average SL is 244 cm, PA is 158º and speed estimation is 7.2 km/h. Mean PL is 40 cm and PW 47 cm. Quality of the tracks is of grade 2. Tracks are almost as long as wide, with a very weak mesaxony. Sometimes one pad per digit is recognizable, although the general shape strongly recalls morphotype II *sensu* [20]. Trackway configuration is very irregular, with a marked sinusoidal and intermittent aspect caused by differences in pace lengths and directional changes. Tracks R9 and L10 are parallel, both showing a small inward rotation and are considered as ‘standing-still’ tracks. Moreover, a directional change is also visible right after the ‘standing still’ tracks, indicating that the trackmaker is milling on the tracksite along its course. Track L11 has a peculiar very elongated heel (metatarsal) impression.

**Trackway BEB500-TR8 (S3)**

Description: 32-tracks discontinuous trackway located in excavation area 1. Total length of the trackway is 40.3 m. It crosses BEB500-TR7 (see above), ten small tridactyl trackways, two sauropod trackways, and it is parallel to three other small tridactyl trackways. It has a very narrow gauge as expressed by a [WAP/PL]-ratio of 0.3. Average PaL is 104 cm for the left-right pace and 106 cm for the right-left pace. Average SL is 206 cm, PA is 168º and speed estimation is 6.5 km/h. Mean PL is 34 cm and PW is 30 cm. Quality of the tracks is of grade 2. Tracks are almost as long as wide; phalangeal pads are present in R2, R3, R7, R12, L13, R13, L14, R14, L15, and L17; a single claw mark occurs only in dIII of R14. The heel area is poorly preserved.

**Interpretation of trackways BEB500**

Trackways TR1 to TR5, TR7 and TR8 (S3, S4), from the BEB500 level share common features in their track morphology. They are all almost as long as wide, subsymmetric, with robust and blunt toes; TR3, TR4, TR7 have no evidence for discrete digital pads and claws, whereas TR1, TR2, TR5 and TR8 sporadically show some phalangeal pad impressions and claw marks. Generally, the best-defined digit is digit III, which is separated from digits II and IV and has a slightly angular, trapezoidal (clover-like) shape with the maximum width located in the middle to anterior part of the digit. Digits II and IV, independently from the occurrence of phalangeal pad impressions, are less well-defined, more oval and more or less merged in a rather short, broad and rounded heel, without evidence for a postero-medial indentation on the heel, which is typically seen in theropod tracks and also in *M. transjuranicus*.

Morphology in TR3, TR4 and TR7 (S3, S4) is consistent throughout the trackways and strongly recalls that of Morphotype II. Compared to *Megalosauripus* tracks, these tracks are wider (less slender) and less mesaxonic and they have a particular elongated trapezoidal shape of digit III and oval and blunt digits II and IV without evidence for impressions of phalangeal pads and claw marks. Generally, the sinusoidal or straight trackways are characterized by a narrow gauge where pes tracks intersect or touch the trackway midline.

BEB500-TR7 (S4) shows a slightly wider gauge with tracks aligned in a zig-zag fashion. Because speed estimations are all indicating slow moving trackmakers, and because tracks do not appear to be susceptible to a clear intra-trackway morphological variability due to changing substrate properties or kinematics, this trackway cannot be assigned to *Megalosauripus*. Instead, these trackways are tentatively assigned to *Iguanodontipus*? *oncalensis* (*sensu* Castanera et al., 2013),

Trackways TR1, TR2, TR5 and TR8 (S3), on the other hand, present some intra-trackway morphological variations, due to the occasional occurrence of phalangeal pad impressions and claw marks. Because of these features, and considering also the overall morphology of these tracks, they are most likely preservation variants of *Megalosauripus* *transjuranicus*, even though they are also slightly similar to *Therangospodus* *pandemicus* (see discussion).

**Porrentruy—CPP (a.k.a. ‘Dinotec’, POR–CPP) tracksite**

**Level 500**

**Trackway CPP500-T1 (S5)**

Description: Discontinuous trackway with 8 tracks preserved within a total length of about 12 m, crossing two parallel trackways of small sauropods and one trackway of a medium-sized sauropod.

Average PL is 37.4 cm and PW is 27.9 cm. IIIV divarication angle is 38°. The mean track rotation is low, slightly inward (-4°) for the left and slightly inward (4°) for the right pes tracks. It has a narrow gauge as expressed by a [WAP/PL]-ratio of 0.4. Average PaL is 80.5 cm for the left-right pace and 87.7 cm for the right-left pace. Average SL is 158 cm, PA 157º and speed estimation 3.8 km/h. Several tracks but notably L8 (S5) are well preserved (grade 2.5), exhibiting slender and separated digits with phalangeal pads and three claw mark impressions. Several tracks (L6–L8) are protected *in situ* in a showcase in the schoolyard of the school ‘Ecole des métiers techniques de Porrentruy’ (CEJEF – Division Technique, DIVTEC).

Interpretation: Because this trackway exhibits phalangeal pads, claw marks, and rather slender and well-separated digits, it can be assigned to *Megalosauripus* ?*transjuranicus*. This is the only trackway on level 500 that can be clearly assigned to *Megalosauripus* ?*transjuranicus,* documenting that Morphotype II trackway BEB-TR7 (S4) is associated with *Megalosauripus* ?*transjuranicus* tracks on the same level (level 500), even though not at the same site (ichnoassemblage). This implicates the following two hypotheses: (1) A distinct Morphotype II left by another tridactyl trackmaker that co-occurs with *Megalosauripus transjuranicus*, or (2) Morphotype II tracks are preservation variants of *Megalosauripus transjuranicus* tracks. These two hypotheses are further analyzed in the discussion.

**Courtedoux—Sur Combe Ronde tracksite (CTD–SCR)**

**Level 1000**

**Trackway SCR1000-T18 (S6)**

Description: Trackway T18 is discontinuous, with only 4 tracks preserved within a total length of 11.1 m. It is located in excavation area 2, crossing three sauropod trackways, one of which heading in the opposite direction. With a [WAP/PL]-ratio of 1.0 it has quite a comparatively wide-gauge for a bipedal tridactyl trackway.

The mean rotation of the tracks is low, strongly outward (+17°) for the left and slightly inward (-3°) for the right pes tracks. Due to the many missing tracks of the overall trackway, it was not possible to calculate mean PaL, SL, and PA. Average PL is 27.2 cm and PW is 25.6 cm. Quality of the tracks is of grade 1. Tracks are almost as long as wide, with no clear heel area impressed, non-tapering digits, and notably dIII with a trapezoidal, clover-like shape and an overall symmetric aspect. Track morphologies are very variable along the trackway, with anatomical details only present on the track R1.

Interpretation: Regarding the well-preserved track R2, this track recalls Morphotype II and an ornithopod morphology. However, there is not enough evidence to suggest an ornithopod trackmaker origin based on one single track only. The third digit may simply have indented deeper into the sediment, implying a sensitive influence of kinematics during track formation and which found its expression in this particular track morphology.

**Trackway SCR1000-T23 (S7, S8)**

Description: Seven-tracks continuous trackway located in excavation area 18, crossing a sauropod trackway moving in the same direction and almost parallel to an opposite-directed sauropod trackway. Total length of the trackway is of 8.4 m. It is narrow gauge with a [WAP/PL]-ratio of 0.3. The mean rotation of the tracks is quite low, parallel to the midline for the left (0°) and slightly inward (-7°) for the right pes tracks. Average PaL is 133.5 cm for the left-right pace and 130.0 cm for the right-left pace. Average SL is 267.5 cm, PA is 165º and speed estimation is 8.2 km/h. Mean PL is 41 cm and PW 26.3 cm. Quality of the tracks is of grade 2. Tracks are elongated and narrow, with a pronounced mesaxony. Digits II and IV impressions display a low interdigital divarication angle. Tracks display one pad per digit, usually digit II and IV are not joint in the heel area, giving the track morphology an asymmetric aspect.

Interpretation: Trackway configuration is very regular with a straight disposition of the tracks on the midline, as underscored by the very small difference between right and left pace lengths, and the high pace angulation. Despite the absence of clear phalangeal pad impressions, track morphology is clearly that of a theropod and strongly recalls that of *Megalosauripus ?transjuranicus*.

**Trackway SCR1000-T24 (S8)**

Description: Nine-tracks continuous trackway located in excavation area 18, is crossing three subparallel sauropod trackways with the same orientation and one subparallel overlapping and oppositely-directed sauropod trackway. Total length of the trackway is of 10.2 m. It has a relatively wide gauge with a [WAP/PL]-ratio of 0.8. The mean rotation of the tracks is consistently inward with -7° for the left and -4° for the right pes tracks. Average PaL is 130 cm for the left-right pace and 123.3 cm for the right-left pace. Average SL is 247.6 cm, PA is 157º and speed estimation is 9.8 km/h. Mean PL is 31.6 cm and PW 28 cm. Quality of the tracks is of grade 2. Tracks are elongated, but mesaxony is not so pronounced. Only one pad per digit is discernable and lateral and medial digit II and IV impressions are not joined in the heel area, giving the track morphology an asymmetric aspect.

Interpretation: Trackway configuration is quite irregular, with a "zig-zag" pattern and a clear inward rotation for both right and left tracks. Pace lengths are slightly different between the right and left sides. The wide disposition of the tracks with respect to the midline results in a pronounced pace angulation and comparatively high [WAP/PL]-ratio, despite a rather high speed estimation. This suggests a trackmaker with a wider posture. The general asymmetry of the track morphology due to the presence of a large PIV1 pad suggests an affinity to *Megalosauripus* cf. *transjuranicus*.

**Courtedoux—Bois de Sylleux tracksite (CTD–BSY)**

**Level 1005**

**Trackway BSY1005-T1 (S9)**

Description: Three-tracks continuous trackway located in excavation area 20. Total length of the trackway is of 2.7 m. The gauge is very narrow with a [WAP/PL]-ratio of 0.4. The mean rotation of the tracks is slightly outward rotated for two left pedes (5°), while the only right pes R1 appears to be aligned to the midline (no measurement available). Average PaL is 107 cm for the left-right pace and 83 cm for the right-left pace. Average SL is 221 cm, PA is 163º and speed estimation is 6.6 km/h. Mean PL is 37.3 cm and PW 21.5 cm. Quality of the tracks is between 2.5 and 3. Tracks are elongated and narrow, with a pronounced mesaxony. A clear phalangeal pad configuration is visible, with a formula 2-3-4 (for dII-III-IV) and claw marks on dIV in track L1. Lateral and medial digits II and IV impressions are not joined in the heel area and digit II impression is isolated from digit IV and digit III, giving the track morphology an asymmetric aspect.

Interpretation: Trackway is too short in order to describe its configuration, although the alternating pace lengths pattern together with the low speed estimation and the moderate [WAP/PL]-ratio suggest that the trackmaker was walking quite slowly. Track morphology is very well preserved and the presence of a clear phalangeal pad configuration, with a special emphasis on the PIV1 phalangeal pad diameter (very wide) allow the assignment of these tracks to *Megalosauripus transjuranicus.*

**Level 1010**

**Trackway BSY1010-T1 (S9)**

Description: Eight-tracks discontinuous trackway located in excavation area 20. Total length of the trackway is 12.5 m. The gauge is very narrow with a [WAP/PL]-ratio of 0.4. The mean rotation of the tracks is slightly inward rotated for the left pes (-3°), while right pedes are slightly outward rotated (7º). Average PaL is 140 cm for the left-right pace and 132 cm for the right-left pace. Average SL is 270 cm, PA is 170º and speed estimation is 8.1 km/h. Mean pes length is 41.8 cm and track width 28.5 cm. Quality of the tracks is between 2 and 2.5. Tracks are elongated and narrow, with a pronounced mesaxony. A clear phalangeal pad configuration is visible only for one track (R5), with a formula 2-3-4 (for dII-III-IV) but no clear claw marks. For the rest of the tracks, only one pad per digit is discerned while lateral and medial digits II and IV impressions are separated from one another and do not meet in the heel area. Phalangeal pad PIV1 is very wide and connected to digit IV, giving the track morphology an asymmetric aspect.

Interpretation: This trackway has quite a regular overall configuration as shown by the very small pace length difference between right and left sides. Track morphology is generally well preserved; although a clear phalangeal pad formula of 2-3-4 is only appreciable in one track. Because of the connection of digit IV impression with the wide metatarsal-phalangeal pad (PIV1), this trackway belongs to *Megalosauripus transjuranicus*.

**Level 1015**

**Trackway BSY1015-T1 (S9)**

Description: Two-tracks trackway located in excavation area 20. Trackway length is of 1.6 m. Trackway gauge cannot be determined because the presence of only two tracks. The mean rotation of the tracks is slightly outward rotated for the left pes (5°), while the right pes R1 appears to be aligned to the midline. PaL is 125 cm. Mean PL is 41.5 cm and PW 27.5 cm. Quality of the tracks is of grade 2. Both tracks are elongated and narrow, with a pronounced mesaxony. Lateral and medial digits II and IV impressions are not joined in the heel area and digit II impression is isolated from digit IV. Despite the absence of clear phalangeal pad impressions, track morphology is clearly that of a theropod.

Interpretation: Trackway is too short to describe its configuration. The phalangeal formula of 2-3-4 for digits II-III and IV respectively is not discernible. Track morphology is characterized by one pad per digit, with PIV1 connected to digit IV. Moreover, the PIV1 phalangeal pad diameter is quite wide, allowing the classification of these tracks as *Megalosauripus transjuranicus.*

**Level 1020**

**Trackway BSY1020-T1 (S10)**

Description: Five-tracks continuous trackway located in excavation area 20, crossing a sauropod trackway with the same orientation. Trackway length is 6.1 m. It has a very narrow gauge with a [WAP/PL]-ratio of 0.4. The mean rotation of the tracks is outward for left tracks (13°) and right (2º) to a lesser extent. Average PaL is 137 cm for the left-right pace and 134.8 cm for the right-left pace. Average SL is 252.8 cm, PA is 167º and speed estimation is 7.2 km/h. Mean PL is 42 cm and PW 29.3 cm. Quality of the tracks is of grade 2. Tracks are elongated but not very narrow. Tracks show only one pad per digit and lateral and medial digits II and IV impressions are separated from one another and do not meet in the heel area, giving the track morphology an asymmetric aspect.

Interpretation: Trackway configuration is not very regular, although there is no significant difference in pace lengths between right and left sides. Trackway and footprint morphometric parameters and overall shape correspond to *Megalosauripus ?transjuranicus* because no further morphological details are preserved.

**Level 1025**

**Trackway BSY1025-T1 (S11)**

Description: Eleven-tracks discontinuous and partial trackway located in excavation area 20. Trackway length is 17.2 m. The gauge is very narrow with a [WAP/PL]-ratio of 0.4. The mean rotation of the tracks is slightly outward rotated for the left pes (4°) and right pes (2º) tracks. Average PaL is 128.9 cm for the left-right pace and 135 cm for the right-left pace. Average SL is 263 cm, PA is 167º and speed estimation is 8 km/h. Mean PL is 40.6 cm and PW 26.1 cm. Quality of the tracks is between grades 2 and 2.5. Tracks are elongated and narrow, with a moderate to pronounced mesaxony. A clear phalangeal pad configuration is appreciable in most of the tracks, with a 2-3-4 (for dII-III-IV) formula and claw marks in digits II and III of track R4. Lateral and medial digits II and IV impressions are not joined in the heel area and digit II impression is isolated from digit IV (apart from track L7) and digit III, giving the track morphology an asymmetric aspect.

Interpretation: This trackway has quite a regular overall configuration as shown by the very small difference in PaL between right and left sides. Track morphology is generally very well preserved, and a clear phalangeal pad formula of 2-3-4 for dII-III-IV is visible in the majority of the tracks. However, it is worth mentioning here that track morphology along this trackway is susceptible to pronounced variations with the presence of a continuum of morphologies from *Megalosauripus transjuranicus* (i.e. tracks L3, R3, R5) over *Megalosauripus* ?*transjuranicus* (i.e. tracks R1, L5, L6) to Morphotype II *sensu* [20] in track LP7. However, because of the features of the best-preserved tracks, this trackway is assigned to *Megalosauripus transjuranicus*.

**Trackway BSY1025-T2 (S11)**

Description: Eight-tracks discontinuous trackway located in excavation area 20. The trackway is 11.7 m long. The gauge is very narrow with a [WAP/PL]-ratio of 0.4. The mean rotation of the tracks is slightly outward oriented for the left tracks (3°) and in a greater extent for the right tracks (9º). Average PaL is 126.8 cm for the left-right pace and 128.5 cm for the right-left pace. Average SL is 254.8 cm, PA is 166º and speed estimation is 7.3 km/h. Mean PL is 42.2 cm and PW 24.5 cm. Quality of the tracks is between grades 2 and 2.5. Tracks are elongated and narrow, with a moderate to pronounced mesaxony. A clear phalangeal pad configuration is appreciable in most of the tracks, with a 2-3-4 (for dII-III-IV) formula and claw marks on all digits in track R1.

Interpretation: Trackway with a quite regular overall configuration as shown by the very small pace length differences between right and left sides. Track morphology is generally well preserved, the clear phalangeal pad formula of 2-3-4 is appreciable in the majority of the tracks, even if track morphology is susceptible to some small changes along the trackway. Nevertheless, a wide and very well developed metatarso-phalangeal pad (PIV1) is quite consistently visible, and this is the diagnostic feature to assign this trackway to *Megalosauripus transjuranicus.*

**Trackway BSY1025-T3 (S10)**

Description: Three-tracks continuous trackway located in excavation area 20, subparallel to an oppositely-directed sauropod trackway. The trackway is 2.6 m long. It has a narrow gauge with a [WAP/PL]-ratio of 0.3. The mean rotation of the tracks is outwardly rotated for left tracks (8°). Average PaL is 113 cm for the left-right pace and 121 cm for the right-left pace. Average SL is 233 cm, PA is 170º and speed estimation is 7.6 km/h. Mean PL is 36 cm and PW 29 cm. Quality of the tracks is between grades 1 and 2. Tracks have a weak mesaxony and display one thick pad per digit impression, and a poorly-preserved metatarso-phalangeal pad.

Interpretation: Trackway configuration is not very regular. Track morphometric parameters and overall shape allow an identifiction as *Megalosauripus ?transjuranicus,* in open nomenclature because not all the characteristic morphological details are preserved.

**Level 1035**

**Trackway BSY1035-T1 (S12)**

Description: Three-tracks continuous trackway located in excavation area 20, parallel to a sauropod trackway. The trackway is 3 m long. The gauge is narrow with a [WAP/PL]-ratio of 0.5. The mean track rotation is outward for the right pes (13°), but this parameter is measured from one track only, as the other two are too poorly preserved. Average PaL is 138 cm for the left-right pace and 139 cm for the right-left pace. Average SL is 275 cm, PA is 167º and speed estimation is 8.8 km/h. Mean PL is 40 cm and PW 26 cm. Quality of the tracks is between grades 1 and 2. Tracks are elongated and narrow, with a pronounced mesaxony. A clear phalangeal pad configuration is not appreciable, but all the digits are clearly separated from each other and track R2 shows a large PIV1 connected to digit IV impression.

Interpretation: Trackway is too short to describe its configuration. The occurrence of very large PIV1 phalangeal pad impressions allows the classification of these tracks as *Megalosauripus* cf. *transjuranicus.*

**Trackway BSY1035-T2 (S13)**

Description: Two-tracks partial trackway located in excavation area 20. Total length is of 1.9 m. Trackway gauge cannot be determined because the presence of only two tracks. Average PaL is 143 cm. Mean PL is 43.5 cm PW 25 cm. Quality of the tracks is of grade 2. Tracks are very elongated and narrow, with a pronounced mesaxony. A clear phalangeal pad configuration is appreciable, with a 2-3-4 (for dII-III-IV) formula.

Interpretation: Trackway is too short to describe its configuration. Track morphology is very well preserved and the presence of clear phalangeal pads together with a very large PIV1 phalangeal pad assigns them to *Megalosauripus transjuranicus.*

**Trackway BSY1035-T3 (S13)**

Description: Six-tracks discontinuous trackway, located in excavation area 20, subparallel to trackway T4 and crossing a sauropod trackway. Total length is 8.4 m. The gauge is narrow with a [WAP/PL]-ratio of 0.3. The mean rotation of the tracks is slightly inward rotated for the left pedes (-3°), while right pedes are aligned to the midline (0º). Average PaL is 114 cm for the left-right pace and 131 cm for the right-left pace. Average SL is 235.8 cm, PA is 167º and speed estimation is 6.9 km/h. Mean PL is 39.6 cm and PW 27.7 cm. Quality of the tracks is of grade 2. Tracks are elongated and narrow, with a moderate to pronounced mesaxony. A clear phalangeal pad configuration is appreciable in tracks L2 and R3, with a 2-3-4 (for dII-III-IV) formula.

Interpretation: Trackway with quite a regular and narrow overall configuration, despite some pace length differences between right and left sides. Track morphology is generally well preserved, digit impressions are always clearly separated from each other, and if visible the phalangeal pad formula is 2-3-4. Because of the connection of digit IV impression with the wide metatarso-phalangeal pad (PIV1), this trackway belongs to *Megalosauripus transjuranicus*.

**Trackway BSY1035-T4 (S13)**

Description: Three-tracks partial trackway located in excavation area 20, subparallel to T3. Total length of the trackway is 2.9 m. The gauge is narrow with a [WAP/PL]-ratio of 0.3. Left tacks are slightly inward rotated (-3º). Average PaL is 122 cm for left-to-right pace and 126 cm for right-to-left pace; average SL is 243 cm; PA is 167º; speed estimation is not possible. Mean PL is 41.3 cm and PW 25 cm. Quality of the tracks is of grade 2. Tracks are very elongate and narrow, with a pronounced mesaxony. A clear phalangeal pad configuration is visible in digit III in track L1 (3 pads). All tracks display a wide PIV1 pad impression.

Interpretation: Trackway is too short to describe its configuration. Track morphology is very well preserved and the presence of a large PIV1 phalangeal pad allows the classification as *Megalosauripus transjuranicus.*

**Trackway BSY1035-T5 (S13)**

Description: Three-tracks discontinuous trackway located in the excavation area 20. Total length of the trackway is of 4.3 m. The gauge is very narrow with a [WAP/PL]-ratio of 0. Right tacks are slightly outward rotated (5º). Average PaL, measured for tracks R2-L3 is 118 cm, average SL is 268 cm. PA could not be measured. Speed estimation is 7.8 km/h. Mean PL is 43 cm and PW 27 cm. Quality of the tracks is between grades 2 and 2.5. Tracks are very elongated and narrow, with a pronounced mesaxony. A clear 2-3-4 phalangeal pad configuration is visible in track R1, which also bears a claw mark in digit IV. All tracks display a wide PIV1 pad impression.

**Interpretation:** Trackway is too short and a track is missing to describe its configuration. Track morphology is very well preserved and the presence of a wide PIV1 phalangeal pad allows the classification of these tracks as *Megalosauripus transjuranicus.*

**Trackway BSY1035-T6 (S14)**

Description: Two-tracks trackway located in excavation area 20. Total length of the trackway is 1.9 m. Trackway gauge cannot be determined because the presence of only two tracks. PaL is 150 cm. Mean PL is 40 cm and PW 22 cm. Quality of the tracks is of grade 2.5. Tracks are very elongate and narrow, with a pronounced mesaxony. A clear 2-3-4 phalangeal pad configuration for digits II-III-IV is exhibited in both tracks, which also bear a claw mark on digit III. All tracks display a wide PIV1 pad impression. Track BSY1035-T6-L2 preserved as a track fill (slab number BSY008-330) is one of the paratypes of the new ichnospecies and it exhibits fine details of pad and claw impressions in all three digits.

Interpretation: Trackway is too short to describe its configuration. Track morphology is very well preserved, with anatomical details of phalangeal pads in tracks R1 and L2. The presence of the wide PIV1 phalangeal pad connected to a padded digit IV is diagnostic for the classification of these tracks as *Megalosauripus transjuranicus*.

**Trackway BSY1035-T7 (S12)**

Description: Three-tracks partial trackway located in excavation area 20, crossing a sauropod trackway. Total length is 2.7 m. The gauge is narrow with a [WAP/PL]-ratio of 0.5. The mean rotation of the tracks is outward rotated for the right pes (2°), although this parameter is measured for one track only, as the other two are poorly preserved. Average PaL is 122 cm for the left-right pace and 113 cm for the right-left pace. Average SL is 230 cm, PA is 162º and speed estimation is 6.9 km/h. Mean PL is 38 cm and PW 26.3 cm. Quality of the tracks is between grades 1.5 and 2. Tracks are elongate and narrow. Track R1 does not have an impression of the heel area, but one pad per digit is appreciable, L2 preserves only digits III and IV, R3 has a one pad per digit with digit II-III and IV clearly separated from one another and a wide PIV1 connected to digit IV impression.

Interpretation: Trackway is too short to describe its configuration. The large PIV1 phalangeal pad is diagnostic for *Megalosauripus* cf. *transjuranicus.*

**Trackway BSY1035-T8 (S13)**

Description: Three-tracks partial trackway located in excavation area 20, subparallel to T3. Total length is of 3.1 m. The gauge is very narrow as expressed by a [WAP/PL]-ratio of 0.2. Right tacks are slightly inward rotated (-2º). Average PaL is 130 cm for left-to-right pace and 136.5 cm for right-to-left pace. Average SL is 265 cm. PA is 169º and speed estimation is 8.1 km/h. Mean PL is 40.7 cm and PW 22.7 cm. Quality of the tracks is between grades 2 and 2.5. Tracks are very elongate and narrow, with a pronounced mesaxony. A clear phalangeal pad configuration is appreciable only for digit II in track L2 (2 pads), and digit III in track R2 (3 pads), which also bears a claw mark. All digit impressions are separated from one another, with a clear large PIV1 pad impression connected to digit IV impression.

Interpretation: Trackway is too short to describe its configuration. Track morphology is generally very well preserved and the phalangeal pad configuration for two out of three tracks, together with the presence of a large PIV1 phalangeal pad impression assign this trackway to *Megalosauripus transjuranicus*.

**Level 1040**

**Trackway BSY1040-T1 (S15)**

Description: Seven-tracks continuous trackway located in excavation area 20. Total length of the trackway is 8.2 m. The gauge is very narrow as expressed by a [WAP/PL]-ratio of 0.3. Both left and right pedes are outwardly rotated with 6º and 10º, respectively. Average PaL is 129.7 cm for left-to-right pace and 131 cm for right-to-left pace. Average SL is 258 cm. PA is 167º and speed estimation is 7.8 km/h. Mean PL is 40.5 cm and PW 24 cm. Quality of the tracks is of 2.5. Tracks are very elongate and narrow, with a moderate mesaxony. A clear phalangeal pad configuration 2-3-4 can be identified for digits II-III-IV of all tracks. Claw marks are preserved on digit IV of track R1 (paratype specimen number MJSN-BSY008-339) and R4 and on digit II of track R2. All tracks display a wide PIV1 pad impression, which is connected to dIV impression.

Interpretation: Trackway configuration is quite regular, with a slight ‘zig-zag’ pattern and a marked outward rotation for both right and left tracks. Pace lengths do not display any significant difference between the right and left sides. The [WAP/PL]-ratio suggests a trackmaker with a moderate narrow posture despite the relatively slow speed. These tracks are very well preserved and show all the key features (large PIV1 pad, phalangeal pad formula 2-3-4 and claw marks) of *M. transjuranicus*. Therefore, BSY1040-T1-R1 (specimen MJSN-BSY008-339) was chosen as a paratype for *Megalosauripus transjuranicus*.

**Trackway BSY1040-T7 (S9)**

Description: Eight-tracks discontinuous trackway located in excavation area 20, subparallel to trackway BSY1040-T8 (see below) and crossing several sauropod trackways and theropod trackway BSY1040-T9 (see below). Total length of the trackway is 21 m. The gauge is narrow with a [WAP/PL]-ratio of 0.5. The mean rotation of the tracks is on both sides slightly outwardly rotated (left 1º, right 1º). Average PaL is 125 cm for left-to-right pace and 131.5 cm for right-to-left pace. Average SL is 253.5 cm. PA is 172º and speed estimation is 7.9 km/h. Mean PL is 39.2 cm and PW 19.2 cm. Quality of the tracks is between grades 2 and 2.5. Tracks are very elongate and narrow, with a moderate mesaxony. A clear phalangeal pad configuration of 2-3-4 is present for digits II-III-IV for all tracks. Claw marks are preserved on digits III and IV of tracks R2 and R9, on digits IV of tracks R4 and R5 and on digits II and III of track L9. All tracks display a wide PIV1 pad impression, which is connected to dIV impression.

Interpretation: Trackway configuration is quite regular, even though several tracks are missing. Pace lengths are slightly different between the right and left sides. Tracks are very well preserved: all the diagnostic features for the assignation of these tracks to *Megalosauripus transjuranicus*, such as the presence of a large PIV1 pad, phalangeal pad formula 2-3-4 and claw marks, are discernable.

**Trackway BSY1040-T8 (S16)**

Description: Six-tracks discontinuous trackway located in excavation area 20, crossing three sauropod trackways and trackway T7 (see description above). The gauge is narrow as expressed by a [WAP/PL]-ratio of 0.3. Total length of the trackway is 13.1 m. The mean rotation of the tracks could only be measured for right tracks and is outwardly oriented (+2°). Average PaL is 149.5 cm for left-to-right pace and 145.5 cm for right-to-left pace. Average SL is 304 cm. PA is 170º and speed estimation is 12.7 km/h. Mean PL is 33.7 cm and PW 18.9 cm. Quality of the tracks is of grade 2. Tracks are slightly longer than wide, with no clear heel area impressed and non-tapering digits with a trapezoidal (clover-like) shape and an overall symmetric aspect. Track morphology is characterized by one discernable pad for digit III, while digits II and IV impressions merge together in the posterior margin of the track.

Interpretation: Digit III is strongly indented into the sediment, and due to a great deal of kinematics involved in track formation, these tracks recall Morphotype II and, more generally, ornithopod morphology. However, there is not enough evidence to suggest an ornithopod trackmaker origin and a preservational bias due to limb kinematics is suggested rather than a different trackmaker origin. In fact, trackway configuration parameters all point to a fast-moving trackmaker, with a highly digitigrade posture. High speed is a reasonable explanation of the merging lateral digits and the lack of a clear PIV1 impression.

**Trackway BSY1040-T9 (S17)**

Description: Four-tracks discontinuous trackway located in excavation area 20, crossing trackway BSY1040-T8 (see above) and a sauropod trackway. Total length of the trackway is of 6.5 m. The gauge is narrow with a [WAP/PL]-ratio of 0.5. The mean rotation of the tracks is outwardly rotated for left tracks (2º), and inwardly rotated for right tracks (-4º). Average PaL is 125 cm for left-to-right pace and 131 cm for right-to-left pace. Average SL is 260.5 cm. PA is 166º and speed estimation is 8.4 km/h. Mean PL is 38.8 cm and PW 23.9 cm. Quality of the tracks is between grades 1.5 and 2. Tracks are elongated, have massive digits and a moderate mesaxony. A clear phalangeal pad configuration 2-3-4 is appreciable for digits II-III-IV in track R3. Claw marks are not preserved. Track R3 displays a wide PIV1 pad impression, which is connected to digit IV impression. The other tracks are not well preserved but appear to display separate digit impressions. The overall track morphology is quite variable along the trackway.

Interpretation: Trackway configuration appears to be slightly irregular, with pace lengths slightly different between the right and left sides. The narrow gauge suggests a trackmaker with a fast-walking gait, as shown also by the pace angulation. Track morphology is very variable, with only one track well preserved. This track preserves a large PIV1 pad, a phalangeal pad formula 2-3-4, both diagnostic features for the assignation of the trackway to *Megalosauripus transjuranicus*.

**Courtedoux—Tchâfouè tracksite (CTD–TCH)**

**Level 1000**

**Trackway TCH1000-TR1 (S8, S18)**

Description: Seven-tracks continuous trackway located in excavation area 12, crossing eight sauropod trackways and two small tridactyl trackways. Total length is 7 m. The gauge is very narrow with a [WAP/PL]-ratio of 0.1. The mean rotation of the tracks is outwardly rotated for left tracks (4º), and strongly inwardly rotated for right tracks (-16º). Average PaL is 118 cm for left-to-right pace and 120.4 cm for right-to-left pace. Average SL is 235.8 cm. PA is 171º and speed estimation is 7.3 km/h. Mean PL is 37.6 cm and PW 26 cm. Quality of the tracks is of grade 1.5. Tracks are slightly longer than wide and have a moderate mesaxony. Digit impressions are generally separated from one another, and in tracks L1, R2, L3 and L4 it is possible to distinguish the presence of a metatarso-phalangeal pad connected to digit IV. In tracks R1, L2 and R3, this distinction is not clear because of the merging of lateral and medial digits II-IV in the heel area. Digit impressions do not preserve any phalangeal pads.

Interpretation: Trackway configuration is quite regular and straight forward, and underlined by the high pace angulation and the narrow gauge of the trackmaker. Track morphometric parameters and overall shape are typical for *Megalosauripus*.

**Trackway TCH1000-TR2 (S8, S19)**

Description: Twenty-one tracks continuous trackway, 25.6 m long, located in the excavation area 12, crossing nine sauropod trackways and one small-sized tridactyl trackway and it is subparallel to two other small tridactyl trackways in two opposite directions. The gauge is very narrow with a [WAP/PL]-ratio of 0.1. The mean rotation of the tracks is outwardly rotated for both left tracks (+3º) and right tracks (+6º). Average PaL is 124.6 cm for left-to-right pace and 127.8 cm for right-to-left pace. Average SL is 251 cm. PA is 131º and speed estimation is 8.3 km/h. Mean PL is 37.1 cm and PW 27.3 cm. Tracks are almost as long as wide, with no clear heel area impressed and non-tapering digits with a clover-like shape and an overall symmetric aspect. Quality of the tracks is of 1.5. Track morphologies are very variable along the trackway. Digit impressions, generally separated from one another, are appreciable in tracks L5, R5, R7, L8, R12 and R14. In L10 lateral and medial digits II-IV merge together in a poorly-preserved heel area, but digit III is still well separated and distinguishable. Another morphology is discernible for most of the tracks of this trackway in as far that all digits are merging in a coalesced clover-like shape. Digits impressions do not preserve any anatomical features (phalangeal pads, claws).

Interpretation: Quite irregular trackway. Poorly-preserved tracks are related to a 20º turn obviously affecting track morphologies of L6, R6 and L7. Despite the general intra-trackway variability observed, track morphometric parameters and overall shape reflect the definition of *Megalosauripus ?transjuranicus,* in open nomenclature because no further morphological details are preserved.

**Level 1015**

**Trackway TCH1015-T1 (S20)**

Description: Six-tracks discontinuous trackway located in excavation area 12, crossing seven sauropod trackways. Total length is 8.6 m. The gauge is narrow as expressed by a [WAP/PL]-ratio of 0.4. The mean rotation of the tracks is inwardly rotated for both left tracks (-6º) and right tracks (-10º). Average PaL is 139.3 cm for left-to-right pace and 140 cm for right-to-left pace. Average SL is 275.8 cm. PA is 176º and speed estimation is 10.6 km/h. Mean PL is 34.2 cm and PW 23.2 cm. Quality of the tracks is of grade 2. Tracks are very elongated and narrow, with a moderate mesaxony. A clear phalangeal pad configuration 2-3-4 is appreciable for digits II-III-IV for the majority of the tracks. Claw marks are preserved on digit III of tracks L3 and R3 and on digits III-IV of track L4. Tracks L2 (specimen MJSN-TCH006-1348) and R3 (specimen MJSN-TCH006-1357) exhibit fine details of pads and claws.

Interpretation: Trackway configuration is quite regular; pace lengths do not display any significant difference between the right and left sides. Track morphology is very well preserved, with a special remark to R3 (specimen MJSN-TCH006-1357), in which all diagnostic features for the assignation of these tracks to *Megalosauripus transjuranicus*, such as the presence of a large PIV1 pad, phalangeal pad formula 2-3-4 and claw marks, are discernable.

**Level 1020**

**Trackway TCH1020-T1 (S21)**

Description: Six-tracks discontinuous trackway located in the excavation area 12, with a relatively narrow gauge ([WAP/PL]-ratio of 0.5). Total length of the trackway is 6.6 m. The mean rotation of the tracks is outwardly rotated for both left (+7°) and right (+11°) tracks. Average PaL is 100.5 cm for the left-right pace and 107.3 cm for the right-left pace. Average SL is 205.1 cm, PA is 162º and speed estimation is 7 km/h. Mean PL is 32.4 cm and PW 25.1 cm. Quality of the tracks is of grade 2. Tracks are almost as long as wide. Digits impressions are well defined, with only one pad per digit appreciable, and lateral and medial digit II and IV impressions do not merge in the heel area, giving the track morphology an asymmetric aspect.

Interpretation: Trackway configuration is quite irregular, with a ‘zig-zag’ pattern and a clear outward rotation for both right and left tracks. Pace lengths are slightly different between the right and left sides. The wide disposition of the tracks with respect to the midline results in a pronounced pace angulation, likely due to the relatively low locomotion speed. The general track morphology with the presence of a large PIV1 pad and well-separated and discernable digits suggest a *Megalosauripus* cf. *transjuranicus* affinity.

**Trackway TCH1020-T2 (S22)**

Description: Seven-tracks discontinuous trackway located in excavation area 12, crossing a sauropod trackway and trackways TCH1020-T1 (see above, S21) and TCH1020-T3 (see below, S23). Total length is 10.6 m. It has a wide gauge, [WAP/PL]-ratio (0.6). The mean rotation of the tracks is inwardly rotated for both left (-9°) and right (-9°) tracks. Average PaL is 148.4 cm for the left-right pace and 139.3 cm for the right-left pace. Average SL is 283.5 cm, PA is 164º and speed estimation is 8.6 km/h. Mean PL is 42.8 cm and PW 30 cm. Quality of the tracks is of grade 2. Tracks are longer than wide. Digits impressions are well defined and separated from one another, with only one pad per digit appreciable and lateral and medial digits II and IV impressions not merging in the heel area, giving the track morphology an asymmetric aspect, as exhibited in R1 (specimen MJSN-TCH006-1335) and L2 (specimen MJSN-TCH006-1140).

Interpretation: Trackway configuration is quite irregular, with a ‘zig-zag’ pattern and a clear inward rotation for both right and left tracks. Pace lengths are slightly different between the right and left sides. The wide disposition of the tracks with respect to the midline results in a pronounced pace angulation. The general track morphology with the presence of a large PIV1 pad connected to digit IV impression, and well-separated and discernable digits suggest a *Megalosauripus* cf. *transjuranicus* affinity.

**Trackway TCH1020-T3 (S23)**

Description: Four-tracks partial trackway, located in excavation area 12, crossing trackway TCH1020-T2 (see above) and parallel to an opposite-directed sauropod trackway. Total length of the trackway is 4 m. The gauge is narrow ([WAP/PL]-ratio of 0.5). The mean rotation of the tracks is very outwardly oriented for both left (+12°) and right (+15°) tracks. Average PaL is 135.5 cm for the left-right pace and 133.3 cm for the right-left pace. Average SL is 267 cm, PA is 176º and speed estimation is 8.7 km/h. Mean PL is 38.8 cm and PW 23.3 cm. Quality of the tracks is of grade 1.5 but only two tracks are well discernible; the other two tracks are incomplete. Tracks are longer than wide, with no clear heel area impressed and non-tapering digits with a clover-like shape and an overall symmetric aspect. Track morphologies are very variable along the trackway and poorly-defined digits appear to be separated but not very discernable.

Interpretation: The tracks strongly recall ornithopod morphology but there is not enough evidence to suggest an ornithopod trackmaker origin based on two complete tracks only. These tracks represent a preservational morphotype that recalls Morphotype II *sensu* [20]), which will be discussed later. From the lack of clear pads defining the metatarsal-phalangeal pad region, it is not possible to identify these tracks as *Megalosauripus.*

**Level 1025**

**Trackway TCH1025-T1 (S24)**

Description: Seven-tracks discontinuous trackway located in excavation area 12, crossing a sauropod trackway and trackways TCH1025-T2 (see below, S25, paratype). Total length of the trackway is 8.3 m. It has a comparatively wide gauge with a [WAP/PL]-ratio of 0.8. The mean rotation of the tracks is inwardly rotated for left (-1°) and outwardly rotated for right (+6°) tracks. Average PaL is 112.3 cm for the left-right pace and 130 cm for the right-left pace. Average SL is 231.3 cm, PA is 153º and speed estimation is 6.4 km/h. Mean PL is 40.4 cm and PW 29.4 cm. Quality of the tracks ranges between grades 1.5 and 2. Tracks are longer than wide and generally narrow. Digit impressions are well defined and separated from one another, with only one pad per digit appreciable and lateral and medial digits II and IV impressions not merging in the heel area, giving the track morphology an asymmetric aspect visible in L4 (specimen MJSN-TCH006-1329). When preserved, PIV1 is very well discernible, wide and connected to digit IV impression.

Interpretation: Trackway configuration is quite irregular, with a ‘zig-zag’ pattern. Pace lengths are quite different between the right and left sides. The wide disposition of the tracks with respect to the midline results in a pronounced pace angulation. The general track morphology with the presence of a large PIV1 pad, slightly connected to digit IV impression and well-separated and discernable digits suggest a *Megalosauripus* cf. *transjuranicus* affinity.

**Trackway TCH1025-T2 (S25)**

Description: Three-tracks trackway located in excavation area 12, crossing theropod trackway TCH1025-T1 (see above). Total length of the trackway is 3.4 m. It has a relatively wide gauge as expressed by a [WAP/PL]-ratio of 0.9. The mean rotation of the tracks is registered only of left tracks and is a strong outward rotation (+18°). Average PaL is 142 cm for the left-right pace and 146 cm for the right-left pace. Average SL is 293.5 cm, PA is 162º and speed estimation is 11.3 km/h. Mean PL is 35.5 cm and PW 23.3 cm. Quality of the tracks is between grades 2 and 2.5. Tracks are longer than wide and generally narrow. Digit impressions are well defined and separated from one another, with the typical phalangeal pad configuration of 2-3-4 (for digits II-III-IV) and claw marks in digits III and IV. The metatarso-phalangeal pad PIV1 is very well discernible, wide and connected to digit IV impression. This is especially true for track TCH1025-T2-L1, considered a paratype for the new ichnospecies (specimen MJSN-TCH006-1329).

Interpretation: Trackway configuration is slightly irregular, with a ‘zig-zag’ pattern. Pace lengths are quite different between the right and left sides. The general track morphology evidenced by the presence of a large PIV1 pad, connected to digit IV impression and well-separated and discernable digits assign this trackway to *Megalosauripus transjuranicus*.

**Level 1030**

**Trackway TCH1030-T1 (S26)**

Description: Eight-tracks continuous trackway located in excavation area 12, parallel to an opposite-directed sauropod trackway and crossing trackway TCH1030-T3 (see below) and another sauropod trackway. Total length of the trackway is 9.7 m. The gauge is quite narrow ([WAP/PL]-ratio is 0.3). The mean rotation of the tracks is inward rotated for left (-4°) and outward rotated for right (+1°) tracks. Average PaL is 138.7 cm for the left-right pace and 129.8 cm for the right-left pace. Average SL is 264.4 cm, PA is 170º and speed estimation is 8.1 km/h. Mean PL is 40.5 cm and PW 28.6 cm. Quality of the tracks is between grades 1.5 and 2. Tracks are longer than wide, with no clear heel area impressed and non-tapering digits. Lateral and medial digit impressions are almost not appreciable, with exception of track R4 (specimen MJSN-TCH006-1023), which exhibits separated digits and a discernable PIV1 pad. Track morphologies are very variable along the trackway.

Interpretation: The track R4 (specimen MJSN-TCH006-1023) clearly represents a *Megalosauripus* morphology allowing to classify this trackway as *Megalosauripus* ?*transjuranicus*. Most other tracks recall morphotype II, which in this case is a preservational variant of *Megalosauripus* tracks, related to variable substrate properties and/or trackmaker kinematics.

**Trackway TCH1030-T2 (S27)**

Description: Five-tracks continuous trackway located in excavation area 12, subparallel to two sauropod trackways. Total length is 6.5 m. The gauge is very narrow with a [WAP/PL]-ratio of 0.1. The mean rotation of the tracks is outwardly rotated for both left tracks (+3º) and right tracks (+9º). Average PaL is 150 cm for left-to-right pace and 148 cm for right-to-left pace. Average SL is 301 cm, PA is 170º and speed estimation is 10.6 km/h. Mean PL is 37.9 cm and PW 23.7 cm. Quality of the tracks is between grades 2 and 2.5. Tracks are elongated and narrow, with a moderate mesaxony. A clear phalangeal pad configuration 2-3-4 is appreciable for digits II-III-IV in the majority of the tracks. Claw marks are preserved on digits II-III of tracks L3 (specimen MJSN-TCH006-1022) and R3 (specimen MJSN-TCH006-1034). Track R2 (specimen MJSN-TCH006-1087) exhibits a wide PIV1 connected to digit IV impression. Tracks MJSN-TCH006-1022 and MJSN-TCH006-1087 are paratypes for the new ichnospecies.

Interpretation: Trackway configuration is quite regular, pace lengths not displaying any significant differences between the right and left sides. [WAP/PL]-ratio suggests a trackmaker with a narrow posture, that is moving relatively fast as expressed by the long SL. Track morphology is very well preserved, all the diagnostic features for the assignation of these tracks to *Megalosauripus transjuranicus*, such as the presence of a large PIV1 pad connected to dIV impression, a 2-3-4 phalangeal pad formula, and claw marks, are discernable.

**Trackway TCH1030-T3 (S28)**

Description: Three-tracks discontinuous trackway located in excavation area 12, crossing theropod trackway TCH1030-T1 (see above) and three sauropod trackways. Total length is of 5.1 m. It has a very narrow gauge ([WAP/PL]-ratio of 0). The mean rotation of the tracks could only be measured for left tracks and results in an inward rotation (-12°). Average PaL is 146 cm, average SL 300 cm, and speed estimation is 10.8 km/h. Mean PL is 38 cm and PW 28.5 cm. Quality of the tracks is of grade 2. Tracks are longer than wide and narrow. Digit impressions are well defined and separated from one another. The metatarso-phalangeal pad PIV1 is well discernible, wide and connected to digit IV impression, well visible in track L1 (specimen MJSN-TCH006-1024).

Interpretation: Trackway is too short to properly describe its configuration. The general track morphology evidenced by the presence of a large PIV1 pad, connected to digit IV impression and well-separated and discernable digits suggest a *Megalosauripus* cf. *transjuranicus* affinity.

**Trackway TCH1030-T4 (S29)**

Description: Two-tracks partial trackway located in excavation area 12 and crossing TCH1030-T2. Total length of the trackway is 1.8 m. The gauge is not possible to calculate. Generally, the trackway is too poorly defined to be described. Quality of the tracks is between grades 1 and 1.5. Interpretation: Trackway is too short to properly describe its configuration. The general track morphology is only discernible in track TCH1030-T4-R1, which shows that the track is longer than wide and narrow. These tracks are similar to *Megalosauripus* isp.

**Trackway TCH1030-T5 (S28)**

Description: Two-tracks partial trackway located in excavation area 12. Total length of the trackway is 1.7 m. Trackway gauge cannot be determined because the presence of only two tracks. Average PaL is 125 cm. Mean PL is 34.8 cm and PW 27 cm. Quality of the tracks is of grade 2. Tracks are longer than wide and narrow. Track R1 is not very well preserved but displays a clear separation of digits. Track L1 is very well preserved, with digit impressions well discernible and separated from one another, with a claw mark on digit III, and the metatarso-phalangeal pad PIV1 well discernible, wide and connected to digit IV impression.

Interpretation: Trackway is too short to properly describe its configuration. The general track morphology with the presence of a large PIV1 pad connected to digit IV impression and well-separated and discernable digits suggest a *Megalosauripus* cf. *transjuranicus* affinity.

**Trackway TCH1030-T6 (S30)**

Description: Two-tracks partial trackway located in excavation area 12. Total length is 1.5 m. Trackway gauge cannot be determined because the presence of only two tracks. Average PaL is 107.5 cm. Mean PL is 34.5 cm and PW 24.3 cm. Quality of the tracks is of grade 2.5. Tracks are very elongated and narrow, with a moderate mesaxony. A clear 2-3-4 phalangeal pad configuration is recognizable in all digits II-III-IV with a claw mark on digit III in track L1 (specimen MJSN-TCH006-1319).

Interpretation: Trackway is too short to define the configuration. Track morphology is very well preserved, and track L1 (specimen MJSN-TCH006-1319) is defined as the holotype of the newly-erected ichnospecies *Megalosauripus transjuranicus* (for more detailed descriptions see the diagnosis).

**Trackway TCH1030-T7 (S31)**

Description: Three-tracks continuous trackway located in excavation area 12 with a very narrow gauge ([WAP/PL]-ratio of 0.2). Total length of the trackway is 3.4 m. Orientation could only be measured for right tracks and resulted in an outward rotation (+4º). Average PaL is 137 cm. Average SL is 274.5 cm, with a PA of 173º and a speed estimation of 9.1 km/h. Mean PL is 38.8 cm and PW 22.5 cm. Tracks are very elongated and narrow, with a moderate mesaxony. A 2-3-4 phalangeal pad configuration is discernible for digits II-III-IV. Claw marks are visible for digits III-IV in track R1, and digit IV in track L2 (specimen MJSN-TCH006-1317), which is a paratype of *Megalosauripus transjuranicus*.

Interpretation: Trackway is too short to define its configuration. [WAP/PL]-ratio suggests a trackmaker with a very narrow posture. Track morphology is very well preserved, especially in L2 (specimen MJSN-TCH006-1317), the paratype, with the presence of a large PIV1 pad and claw marks.
