# Supplementary figures and images for: Megalosauripus transjuranicus ichnosp. nov. A new Late Jurassic theropod ichnotaxon from NW Switzerland and implications for tridactyl dinosaur ichnology and ichnotaxomy
Source: PLoS One. 2017 Jul 17;12(7):e0180289. doi: 10.1371/journal.pone.0180289 (PMC5513421; doi:10.1371/journal.pone.0180289)

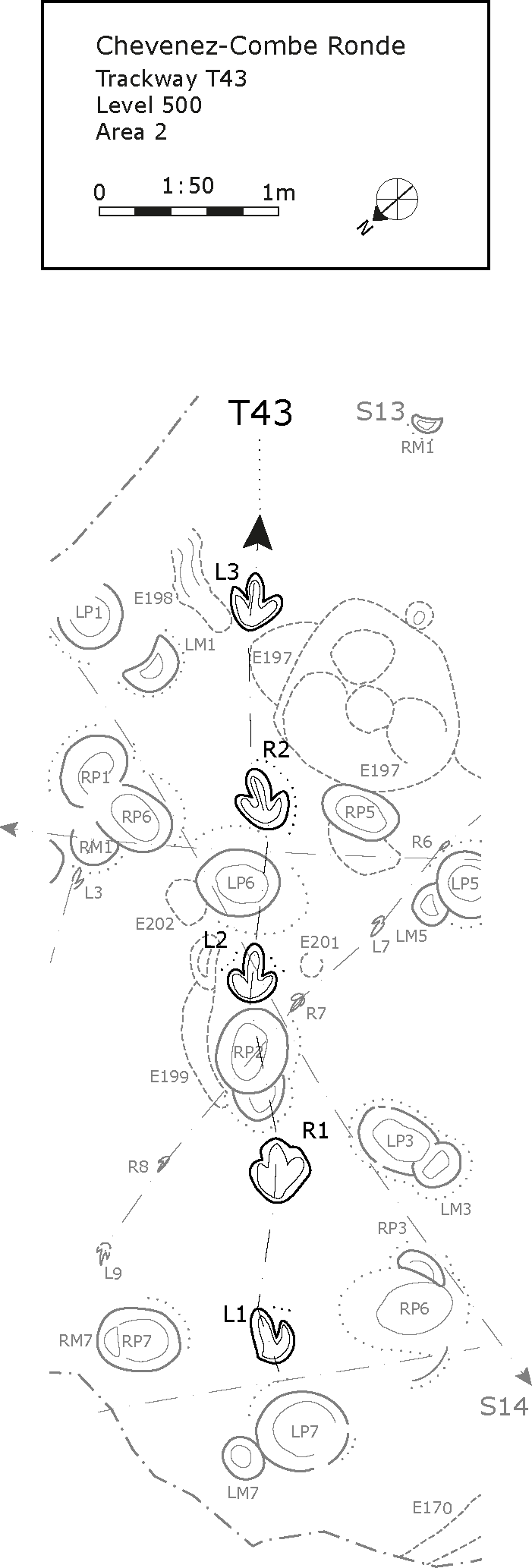

Supplement: S1 Fig — Outline drawing of the trackway (scale 1:50). (TIF) [file pone.0180289.s002.tif]

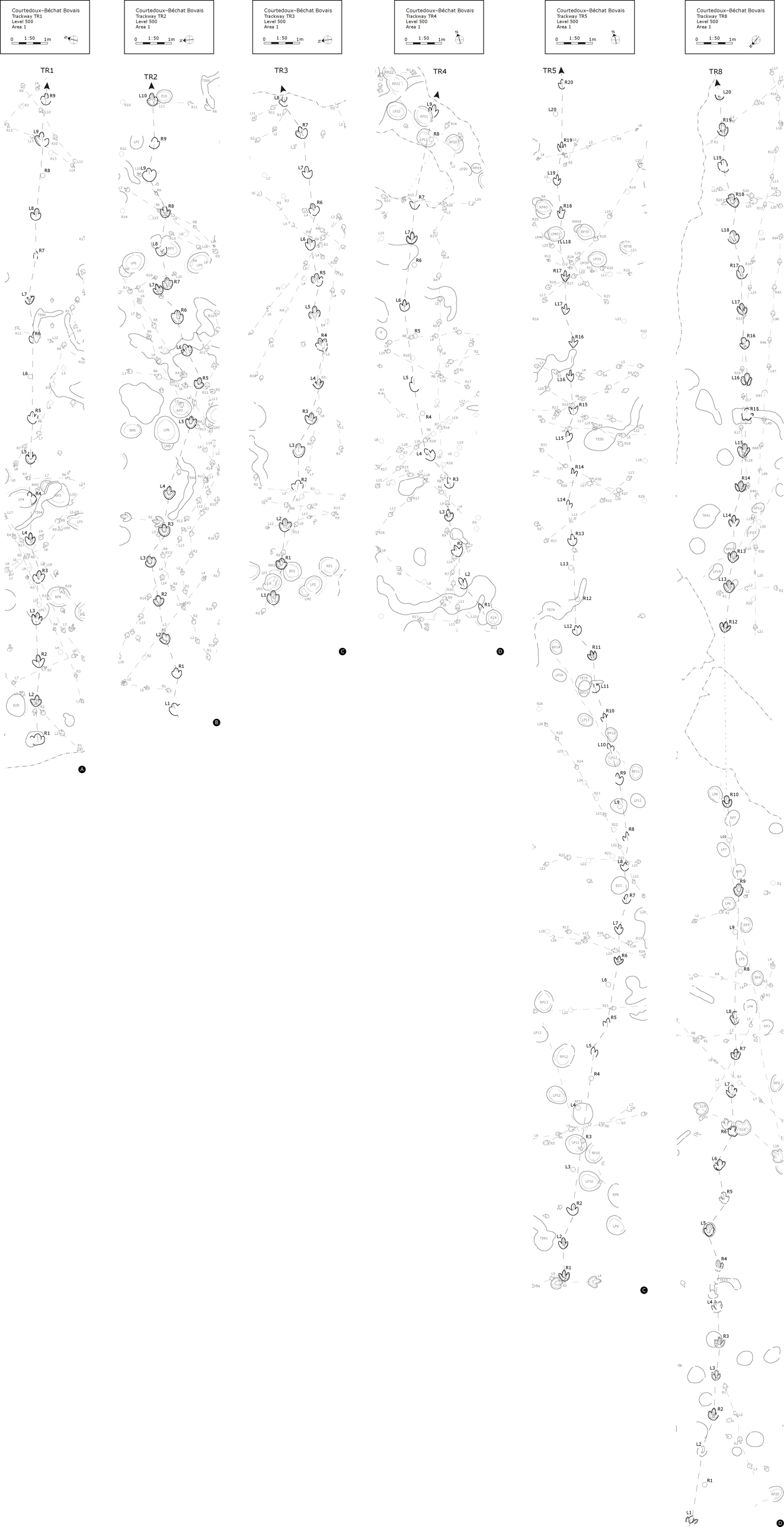

Supplement: S2 Fig — Outline drawings of trackways from BEB500 (scale 1:50). (A) BEB500-TR1. (B) BEB500-TR2. (C) BEB500-TR3. (D) BEB500-TR4. (E) BEB500-TR5. (F) BEB500-TR8. (TIF) [file pone.0180289.s003.tif]

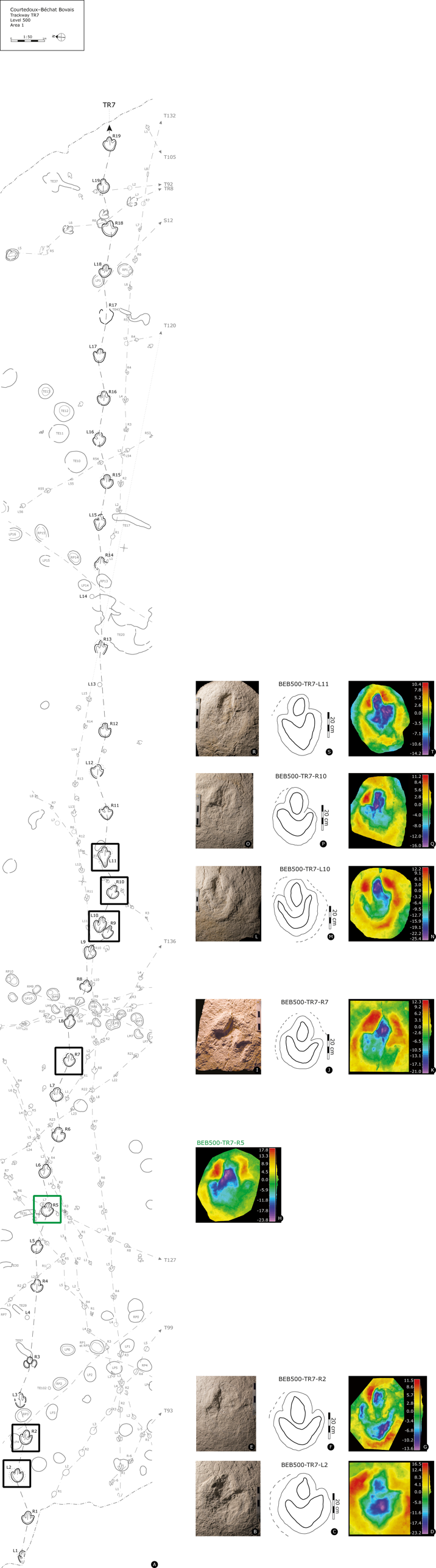

Supplement: S3 Fig — (A) Outline drawing of the trackway (scale 1:50). (B) Photo of BEB500-TR7-L2. Scale bar 20 cm. (C) Interpretative outline drawing of BEB500-TR7-L2. (D) False-color depth map of BEB500-TR7-L2. Depth measured in mm. (E) Photo of BEB500-TR7-R2. Scale 20 cm. (F) Interpretative outline drawing of BEB500-TR7-R2. (G) False-color depth map of BEB500-TR7-R2. Depth measured in mm. (H) False-color depth map of BEB500-TR7-R5 obtained from laserscanner. Depth measured in mm. (I) Photo of BEB500-TR7-R7. Scale 20 cm. (J) Interpretative outline drawing of BEB500-TR7-R7. (K) False-color depth map of BEB500-TR7-R7. Depth measured in mm. (L) Photo of BEB500-TR7-L10. Scale 20 cm. (M) Interpretative outline drawing of BEB500-TR7-L10. (N) False-color depth map of BEB500-TR7-L10. Depth measured in mm. (L) Photo of BEB500-TR7-R10. Scale 20 cm. (P) Interpretative outline drawing of BEB500-TR7-R10. (Q) False-color depth map of BEB500-TR7-R10. Depth measured in mm. (R) Photo of BEB500-TR7-L11. Scale 20 cm. (S) Interpretative outline drawing of BEB500-TR7-L11. (T) False-color depth map of BEB500-TR7-L11. Depth measured in mm. (TIF) [file pone.0180289.s004.tif]

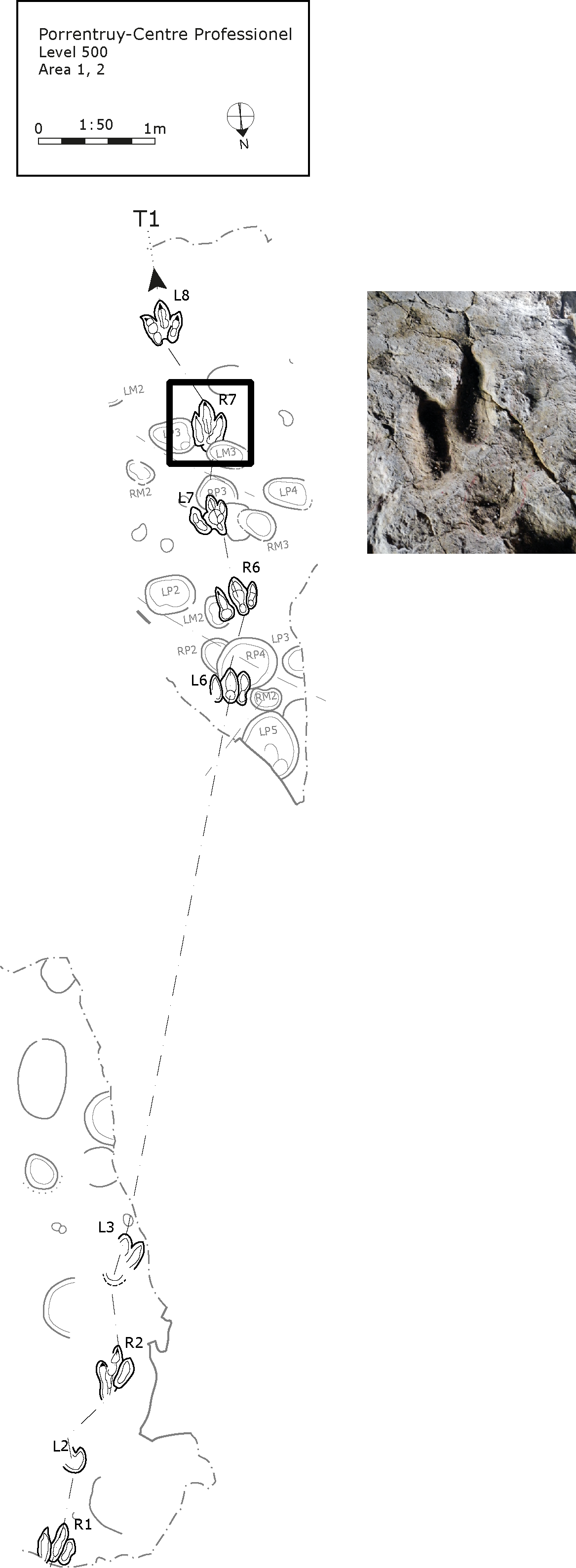

Supplement: S4 Fig — Outline drawing of the trackway (scale 1:50). (TIF) [file pone.0180289.s005.tif]

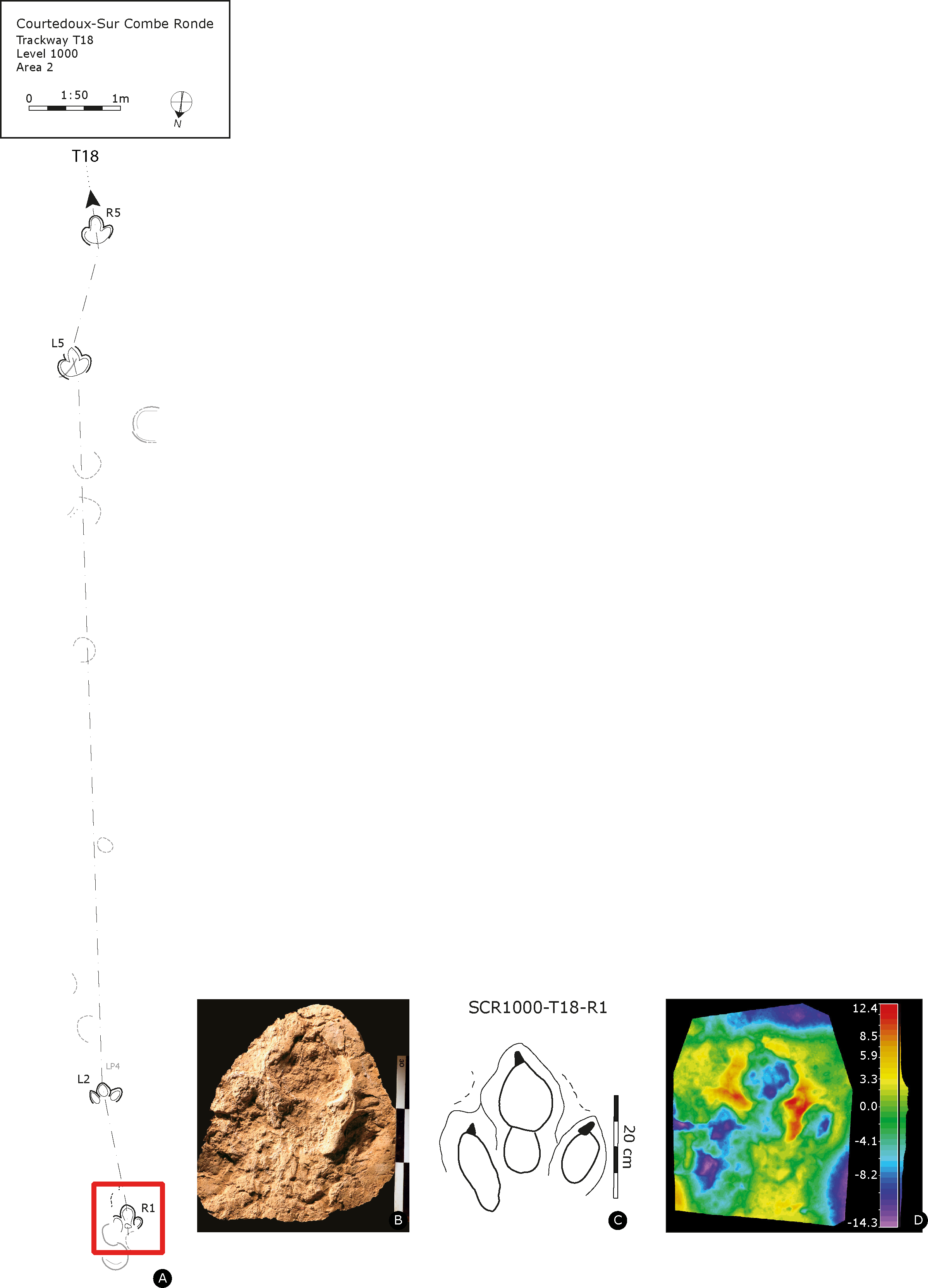

Supplement: S5 Fig — (A) Outline drawing of the trackway (scale 1:50). (B) Photo of SCR1000-T18-R1. Scale bar 20 cm. (C) Interpretative outline drawing of SCR1000-T18-R1. (D) False-color depth map of SCR1000-T18-R1. Depth measured in mm. (TIF) [file pone.0180289.s006.tif]

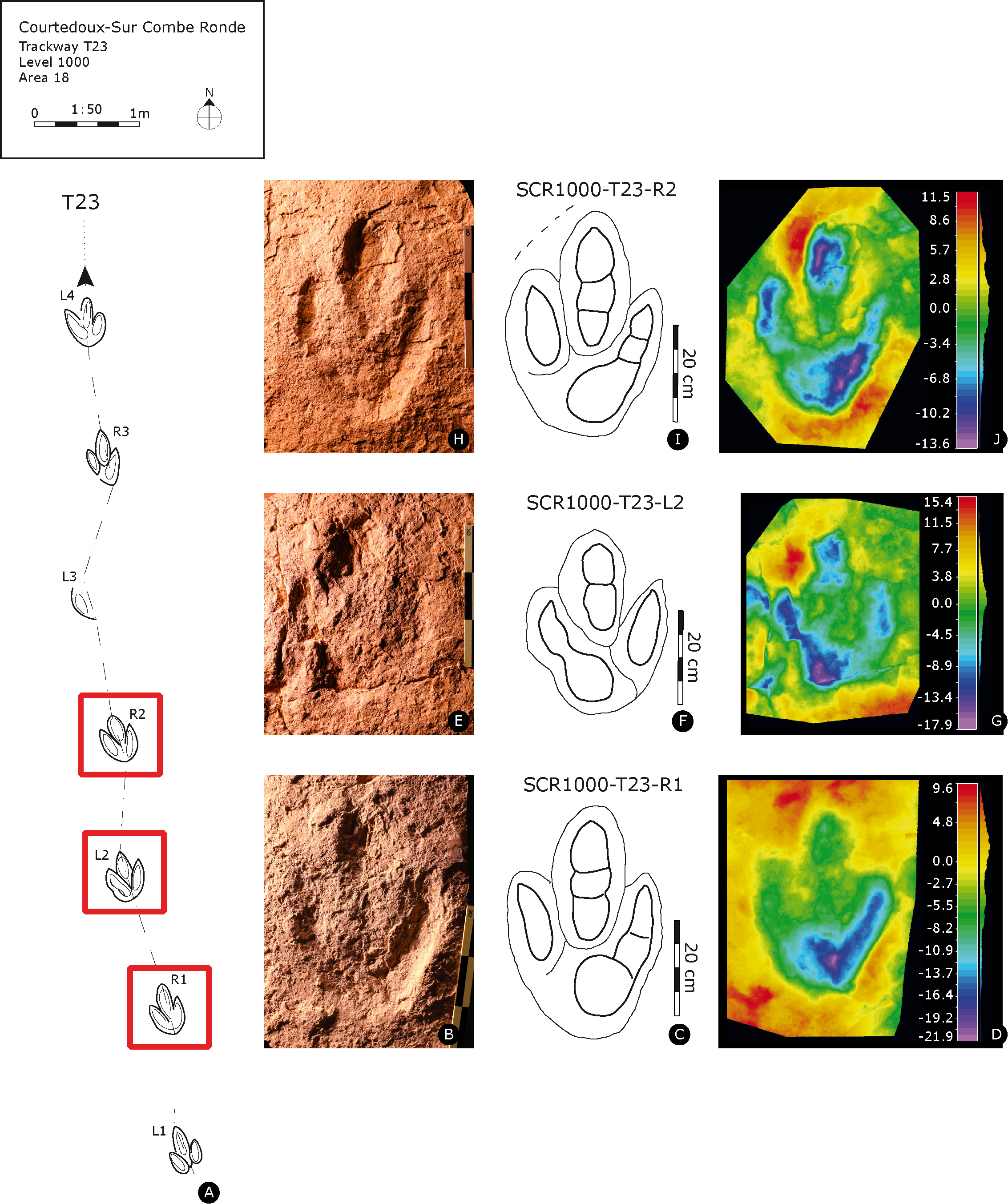

Supplement: S6 Fig — (A) Outline drawing of the trackway (scale 1:50). (B) Photo of SCR1000-T23-R1. Scale bar 30 cm. (C) Interpretative outline drawing of SCR1000-T23-R1. (D) False-color depth map of SCR1000-T23-R1. Depth measured in mm. (E) Photo of SCR1000-T23-L2. Scale 30 cm. (F) Interpretative outline drawing of SCR1000-T23-L2. (G) False-color depth map of SCR1000-T23-L1. Depth measured in mm. (H) Photo of SCR1000-T23-R2. Scale 30 cm. (I) Interpretative outline drawing of SCR1000-T23-R2. (K) False-color depth map of SCR1000-T23-R2. Depth measured in mm. (TIF) [file pone.0180289.s007.tif]

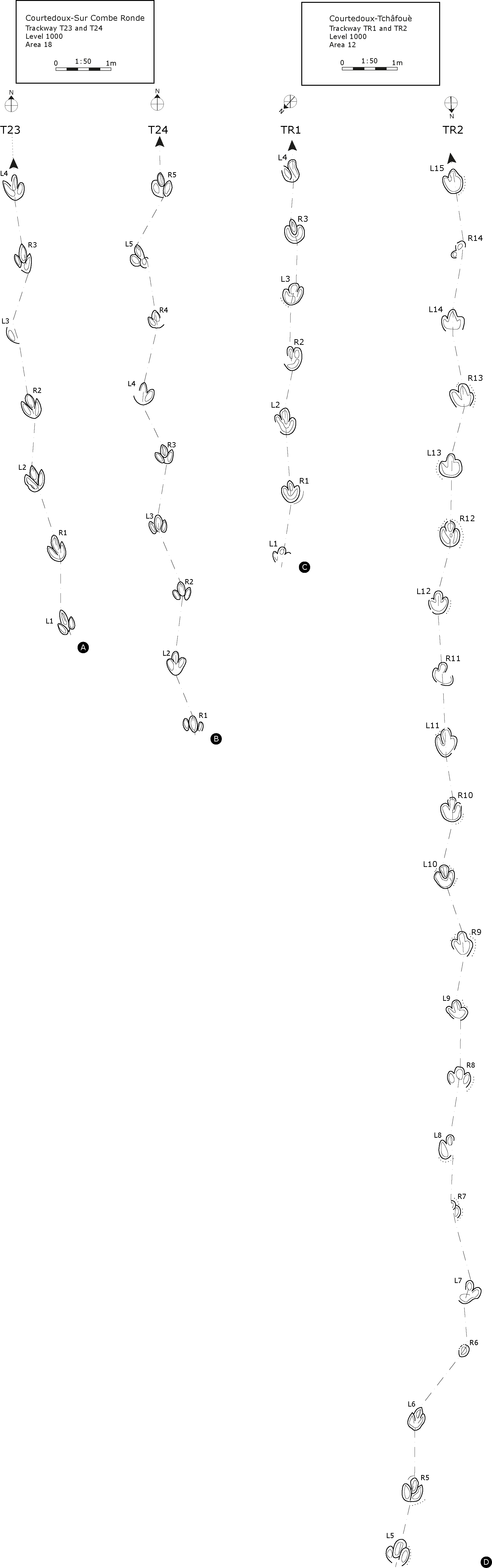

Supplement: S7 Fig — Outline drawings at 1:50 scale of trackways from SCR1000 (A-B) and TCH1000 (C-D). (A) SCR1000-T23. (B) SCR1000-T24. (C) TCH1000-TR1. (D) TCH1000-TR2. (TIF) [file pone.0180289.s008.tif]

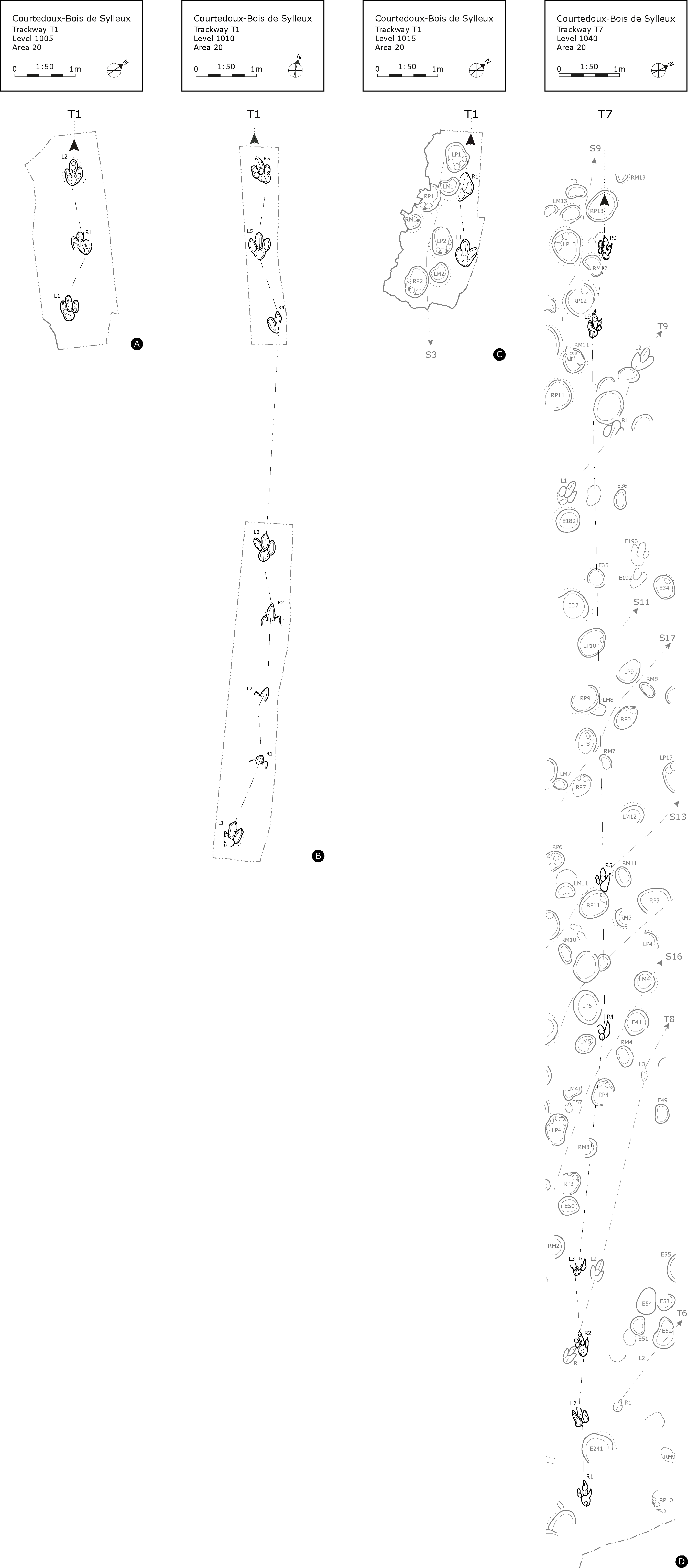

Supplement: S8 Fig — Outline drawings at 1:50 scale of trackways from different levels of BSY. (A) BSY1005-T1. (B) BSY1010-T1. (C) BSY1015-T1. (D) BSY1040-T7. (TIF) [file pone.0180289.s009.tif]

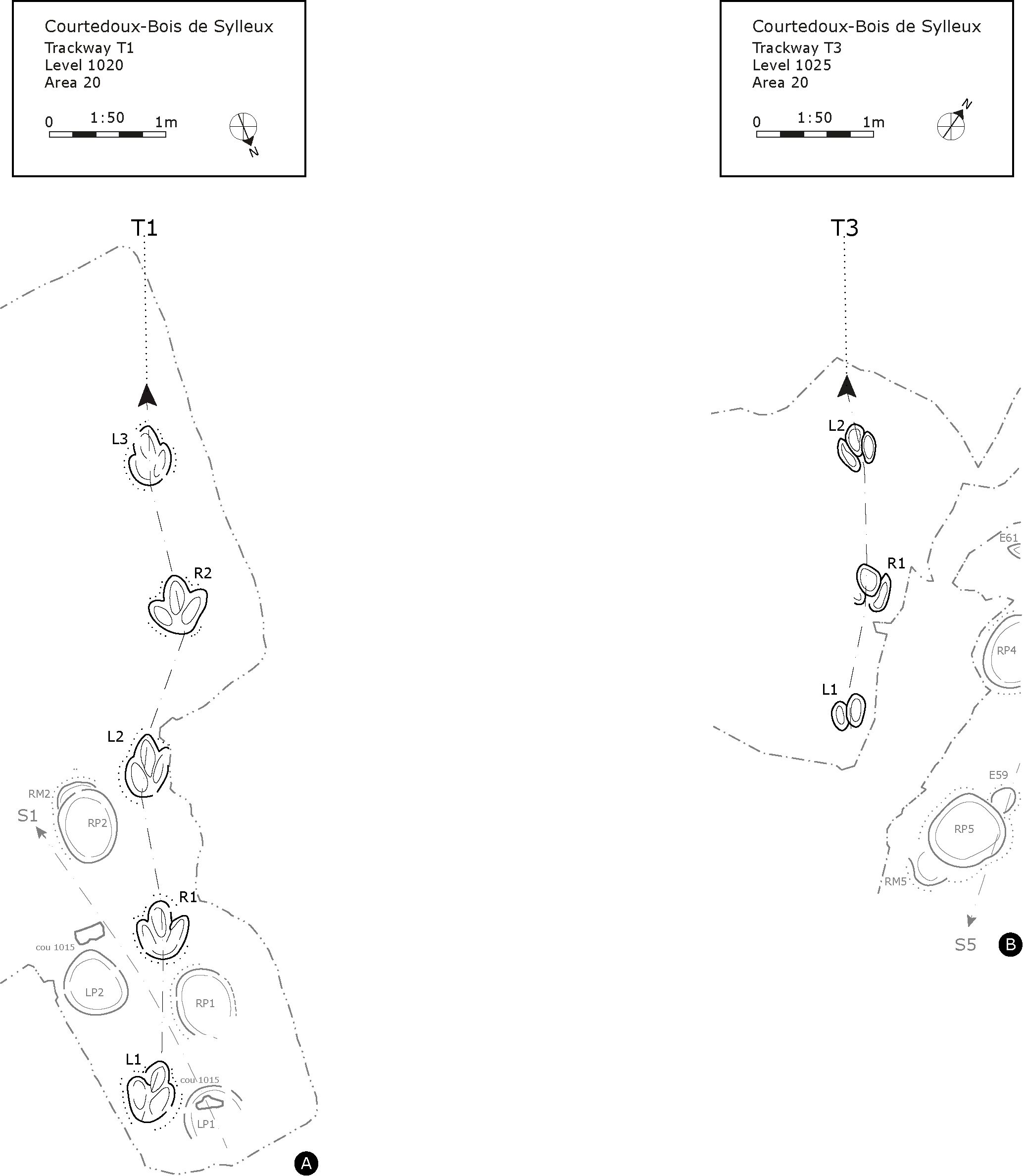

Supplement: S9 Fig — Outline drawings at 1:50 scale of trackways from different levels of BSY. (A) BSY1020-T1. (B) BSY1025-T3. (TIF) [file pone.0180289.s010.tif]

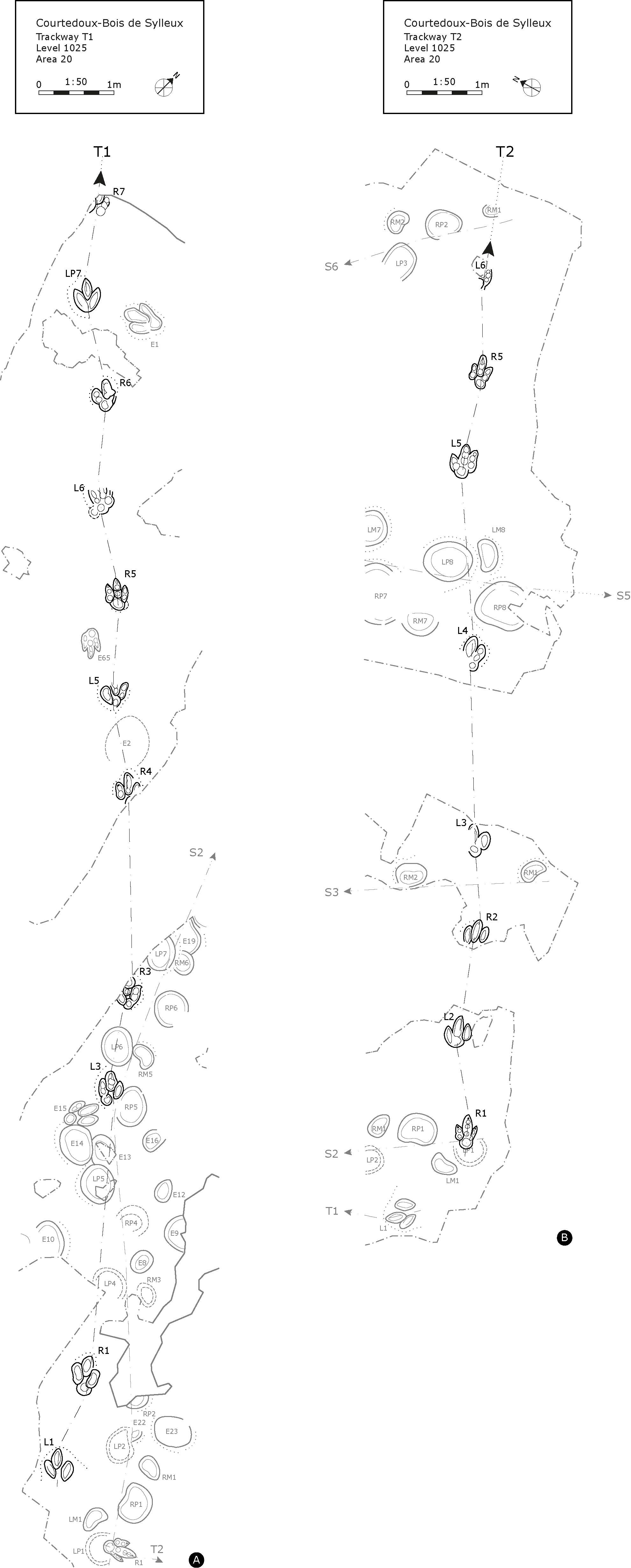

Supplement: S10 Fig — Outline drawings at 1:50 scale of trackways from BSY1025. (A) BSY1025-T1. (B) BSY1025-T2. (TIF) [file pone.0180289.s011.tif]

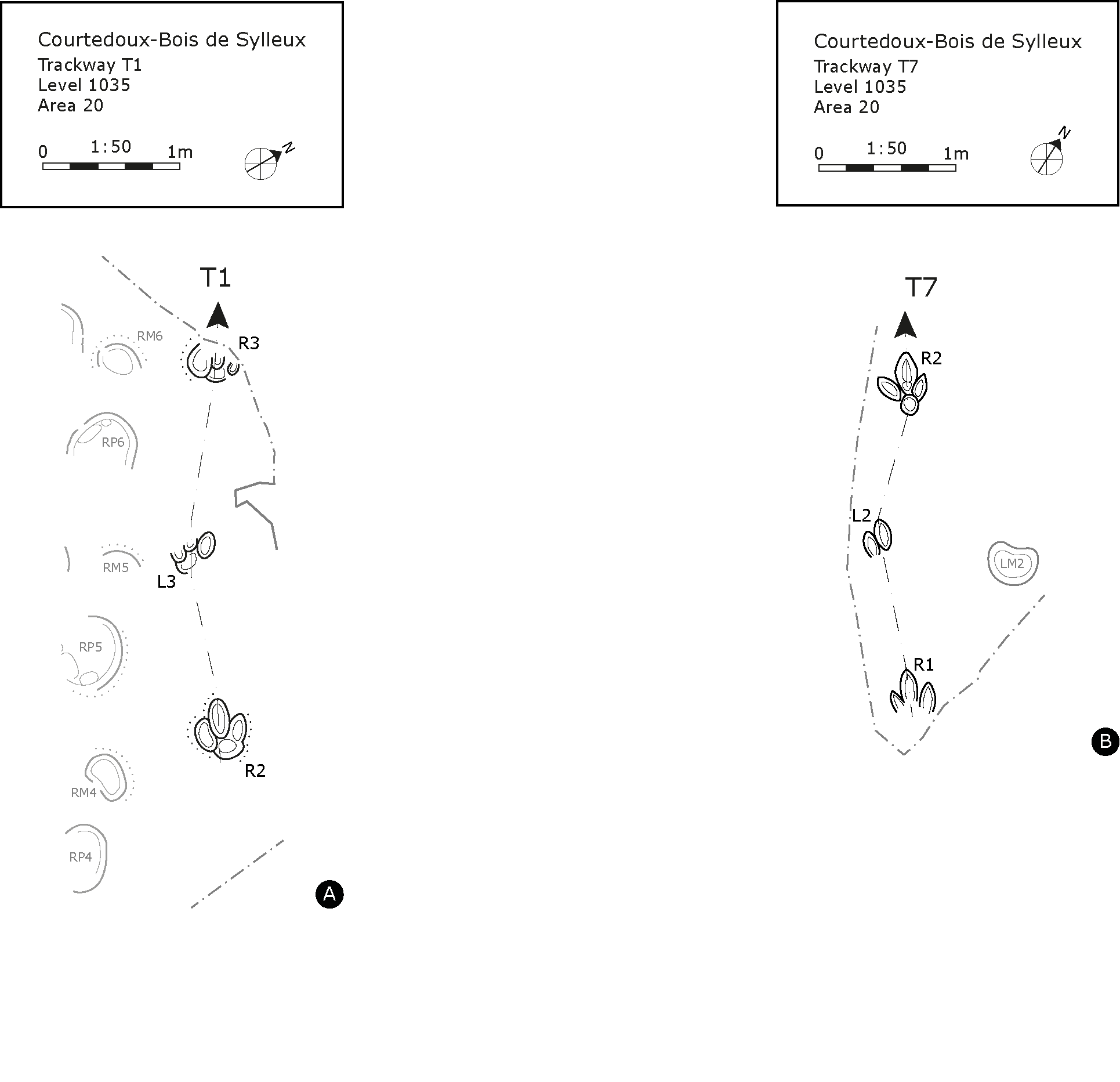

Supplement: S11 Fig — Outline drawings at 1:50 scale of trackways from BSY1035. (A) BSY1035-T1. (B) BSY1035-T7. (TIF) [file pone.0180289.s012.tif]

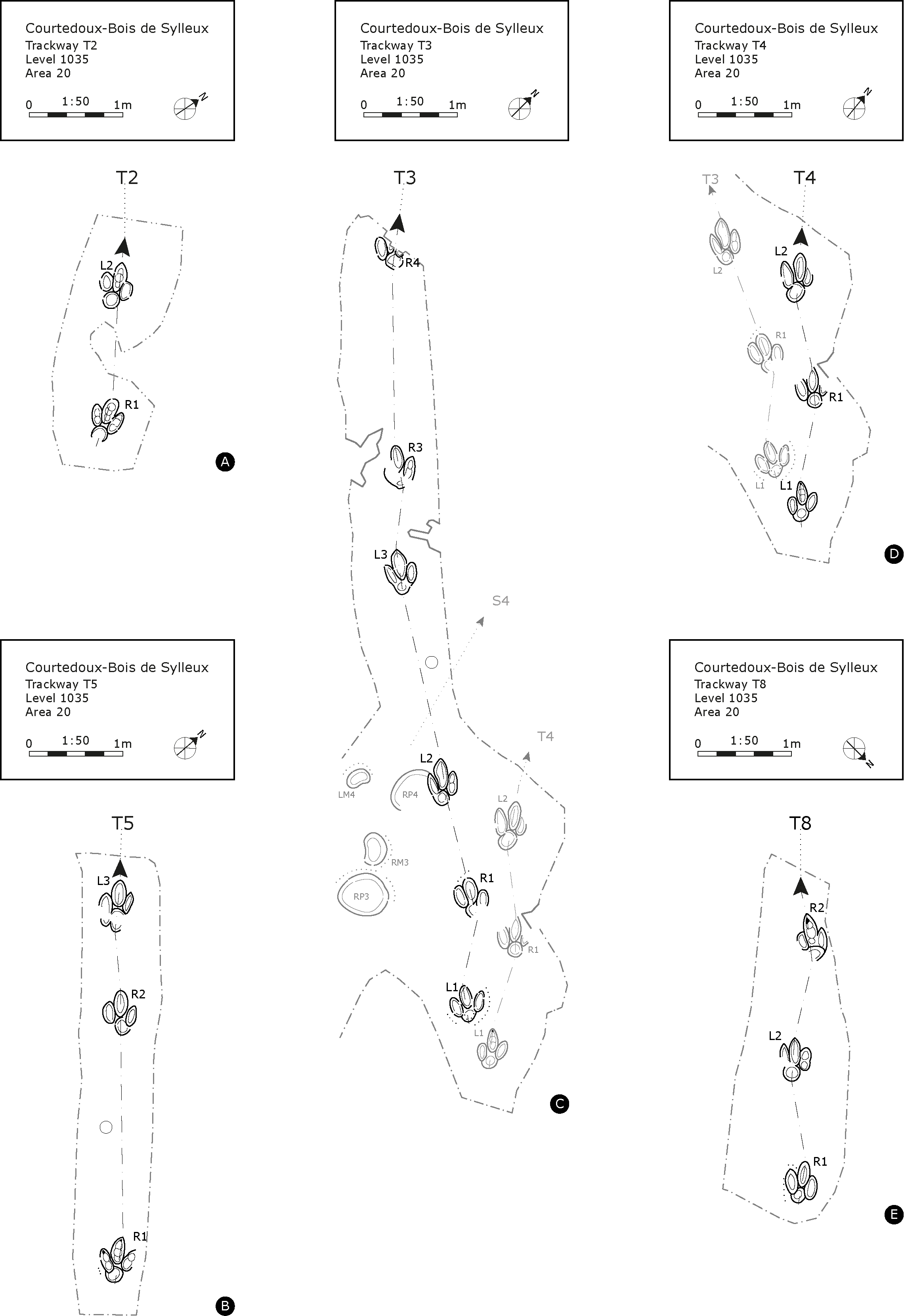

Supplement: S12 Fig — Outline drawings at 1:50 scale of trackways from BSY1035. (A) BSY1035-T2. (B) BSY1035-T5. (C) BSY1035-T3. (D) BSY1035-T4. (E) BSY1035-T8. (TIF) [file pone.0180289.s013.tif]

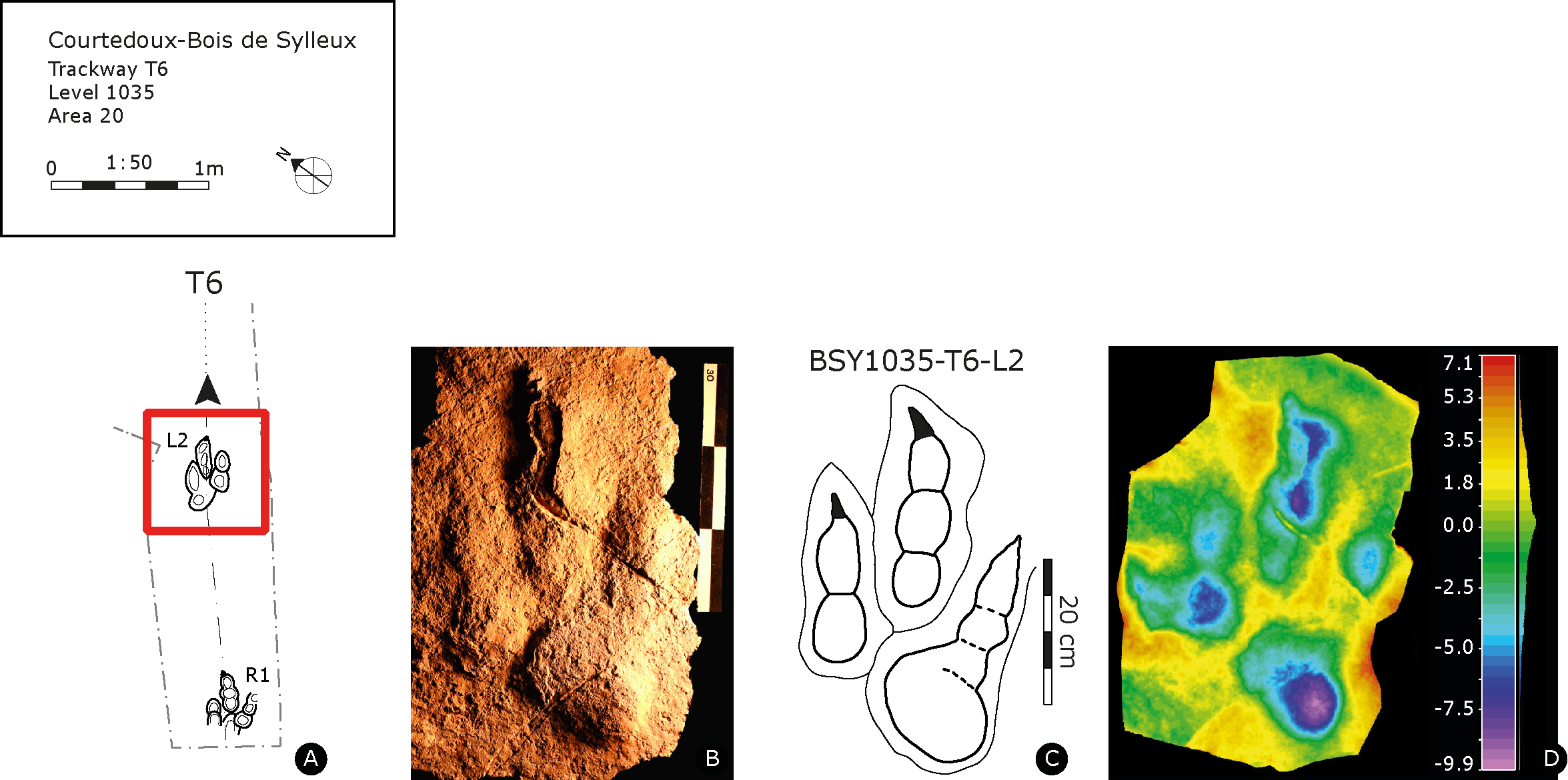

Supplement: S13 Fig — (A) Outline drawing at 1:50 scale. (B) Photo. Scale 30 cm. (C) Interpretative outline drawing. (D) False-color depth map. Depth measured in mm. (TIF) [file pone.0180289.s014.tif]

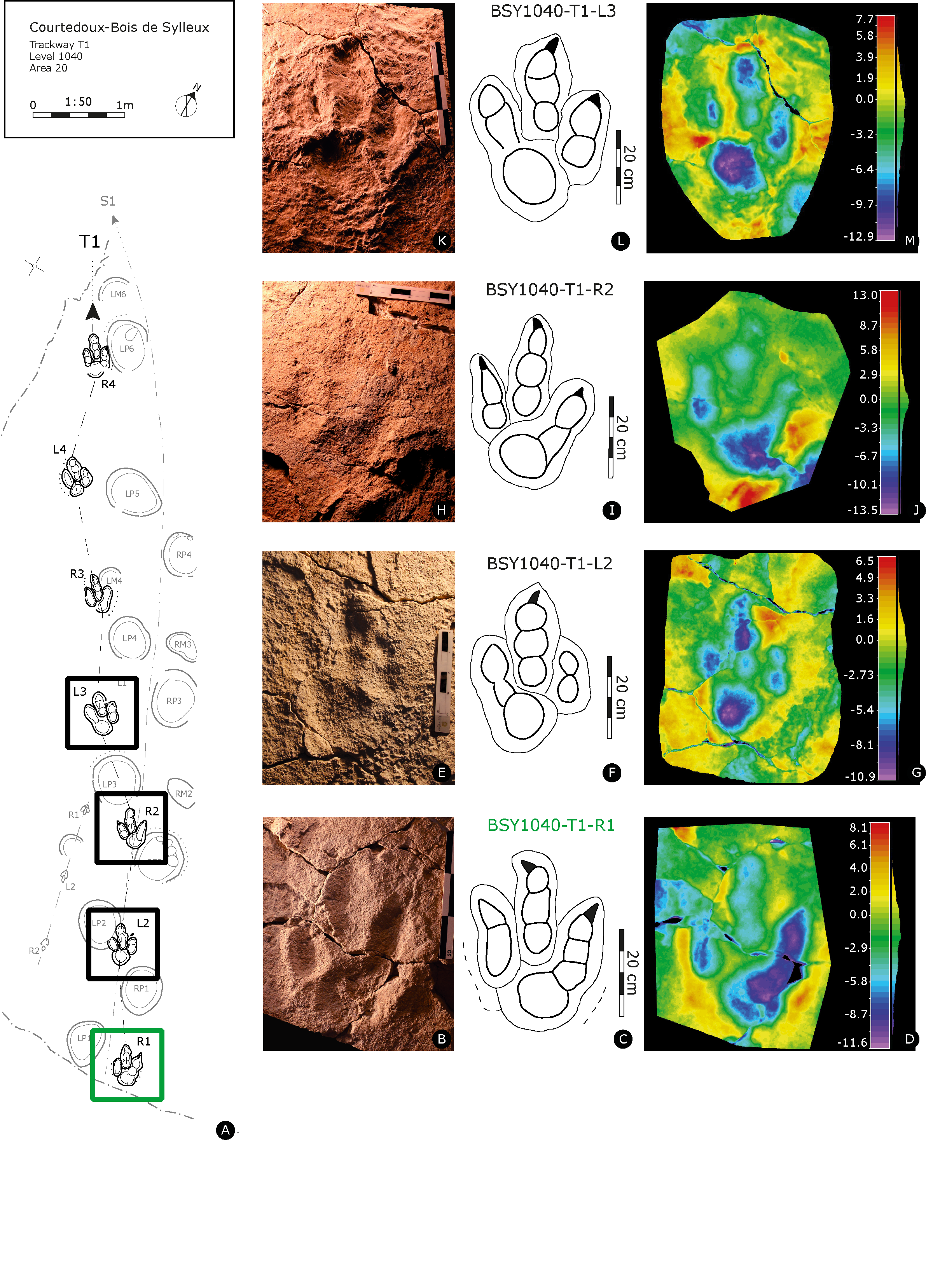

Supplement: S14 Fig — (A) Outline drawing at 1:50 scale of the trackway. (B) Photo of BSY1040-T1-R1 (paratype). Scale bar 20 cm. (C) Interpretative outline drawing of BSY1040-T1-R1. (D) False-color depth map of BSY1040-T1-R1. Depth measured in mm. (E) Photo of BSY1040-T1-L2. Scale bar 20 cm. (F) Interpretative outline drawing of BSY1040-T1-L2. (G) False-color depth map of BSY1040-T1-L2. Depth measured in mm. (H) Photo of BSY1040-T1-R2. Scale bar 20 cm. (I) Interpretative outline drawing of BSY1040-T1-R2. (J) False-color depth map of BSY1040-T1-R2. Depth measured in mm. (K) Photo of BSY1040-T1-L3. Scale bar 20 cm. (L) Interpretative outline drawing of BSY1040-T1-L3. (M) False-color depth map of BSY1040-T1-L3. Depth measured in mm. (TIF) [file pone.0180289.s015.tif]

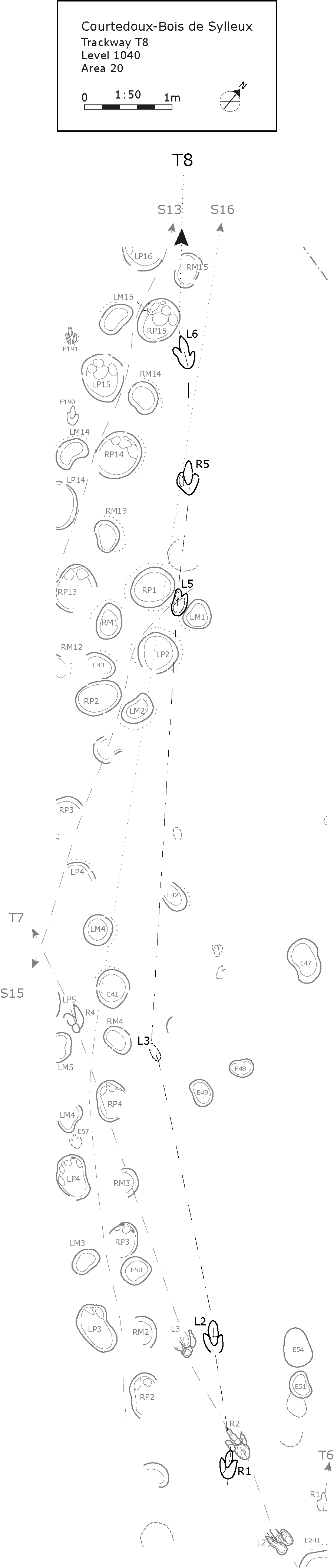

Supplement: S15 Fig — Outline drawing at 1:50 scale of the trackway. (TIF) [file pone.0180289.s016.tif]

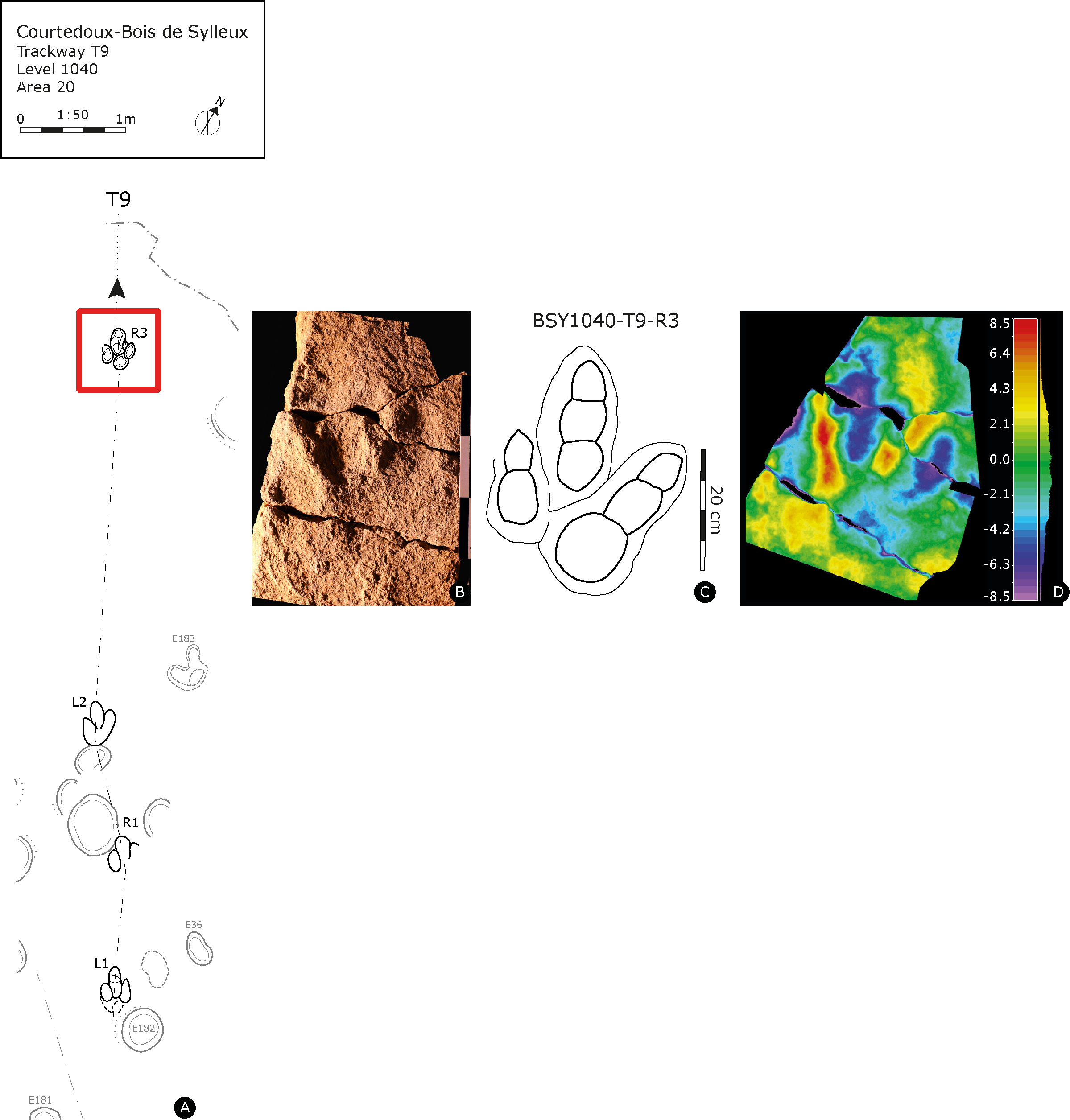

Supplement: S16 Fig — (A) Outline drawing at 1:50 scale of the trackway. (B) Photo of BSY1040-T9-R3. Scale bar 30 cm. (C) Interpretative outline drawing of BSY1040-T9-R3. (D) False-color depth map of BSY1040-T9-R3. Depth measured in mm. (TIF) [file pone.0180289.s017.tif]

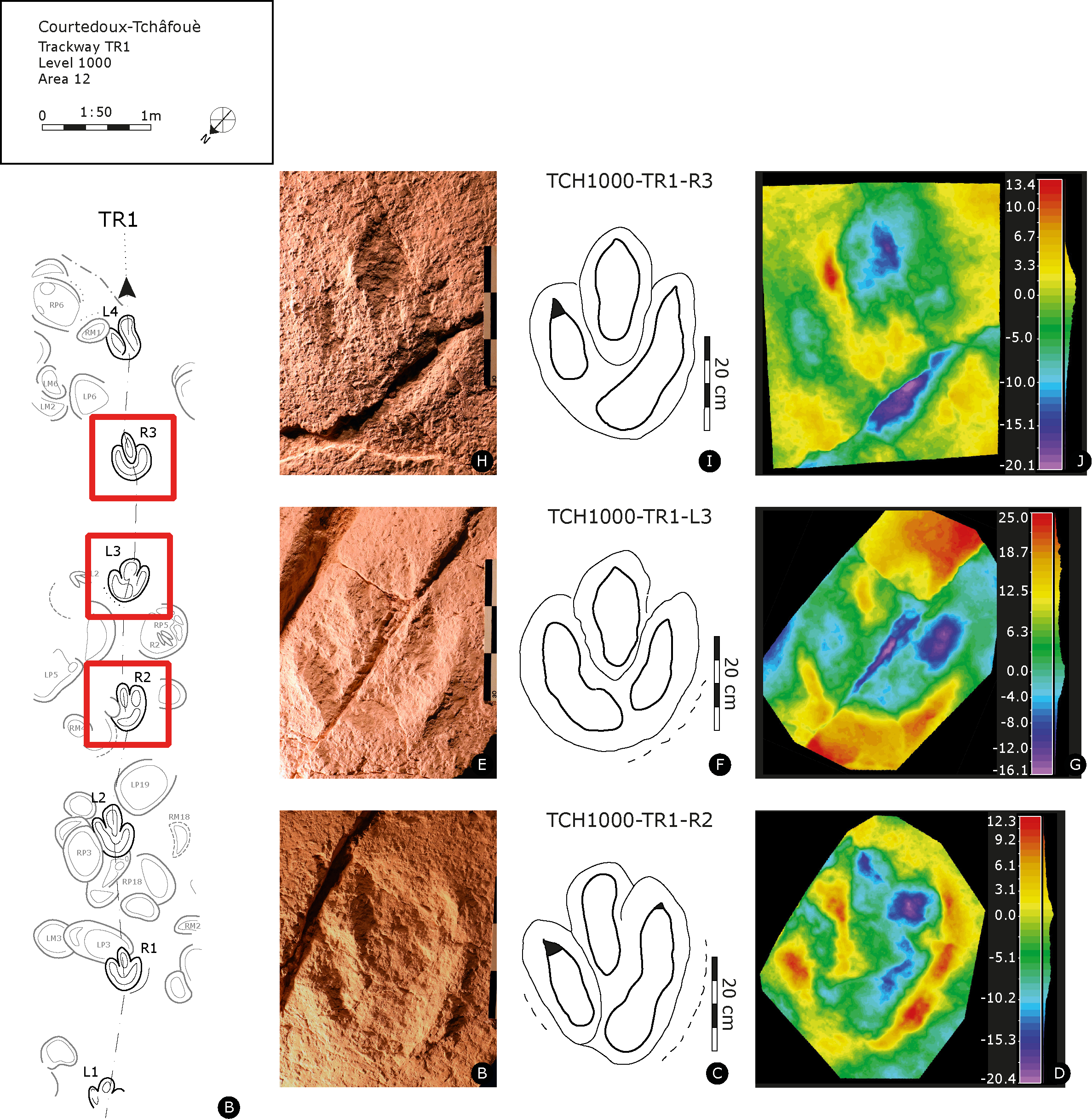

Supplement: S17 Fig — (A) Outline drawing at 1:50 scale of the trackway. (B) Photo of TCH1000-TR1-R2. Scale bar 30 cm. (C) Interpretative outline drawing of TCH1000-TR1-R2. (D) False-color depth map of TCH1000-TR1-R2. Depth measured in mm. (E) Photo of TCH1000-TR1-L3. Scale bar 30 cm. (F) Interpretative outline drawing of TCH1000-TR1-L3. (G) False-color depth map of TCH1000-TR1-L3. Depth measured in mm. (H) Photo of TCH1000-TR1-R3. Scale bar 30 cm. (I) Interpretative outline drawing of TCH1000-TR1-R3. (J) False-color depth map of TCH1000-TR1-R3. Depth measured in mm. (TIF) [file pone.0180289.s018.tif]

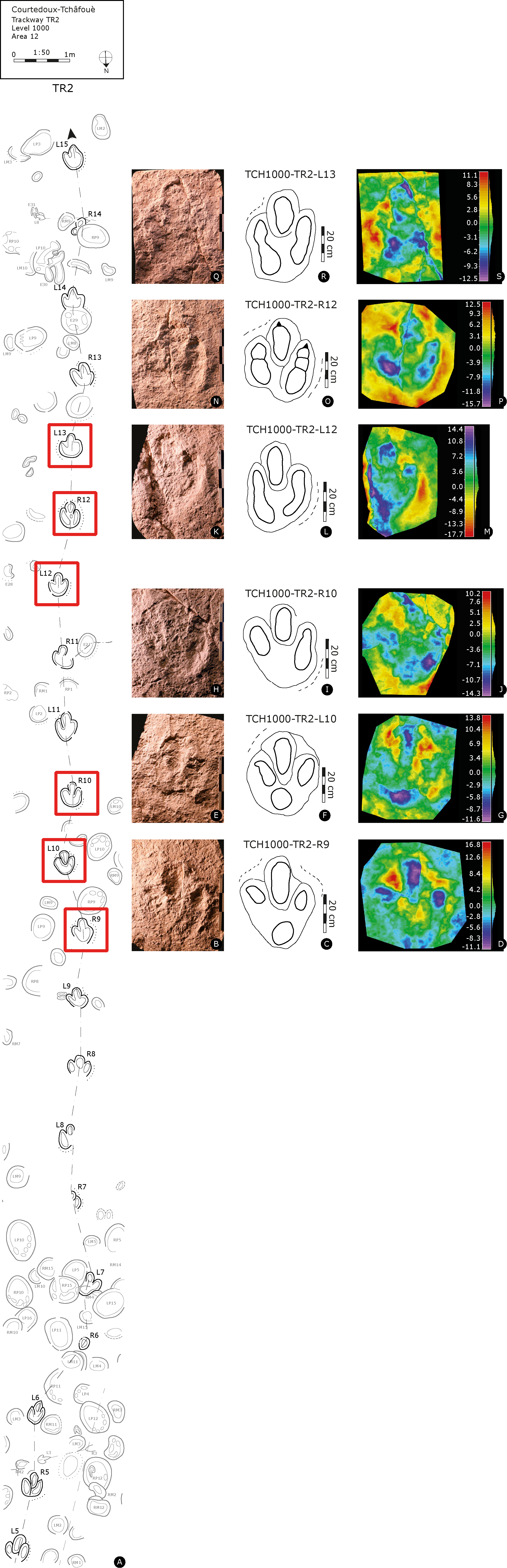

Supplement: S18 Fig — (A) Outline drawing at 1:50 scale of the trackway. (B) Photo of TCH1000-TR1-R9. Scale bar 30 cm. (C) Interpretative outline drawing of TCH1000-TR1-R9. (D) False-color depth map of TCH1000-TR1-R9. Depth measured in mm. (E) Photo of TCH1000-TR1-L10. Scale bar 30 cm. (F) Interpretative outline drawing of TCH1000-TR1-L10. (G) False-color depth map of TCH1000-TR1-L10. Depth measured in mm. (H) Photo of TCH1000-TR1-R10. Scale bar 30 cm. (I) Interpretative outline drawing of TCH1000-TR1-R10. (J) False-color depth map of TCH1000-TR1-R10. Depth measured in mm. (K) Photo of TCH1000-TR1-L12. Scale bar 30 cm. (L) Interpretative outline drawing of TCH1000-TR1-L12. (M) False-color depth map of TCH1000-TR1-L12. Depth measured in mm. (N) Photo of TCH1000-TR1-R12. Scale 30 cm. (O) interpretative outline drawing of TCH1000-TR1-R12. (P) False-color depth map of TCH1000-TR1-R12. Depth measured in mm. (Q) Photo of TCH1000-TR1-L13. Scale bar 30 cm. (R) Interpretative outline drawing of TCH1000-TR1-L13. (S) False-color depth map of TCH1000-TR1-L13. Depth measured in mm. (TIF) [file pone.0180289.s019.tif]

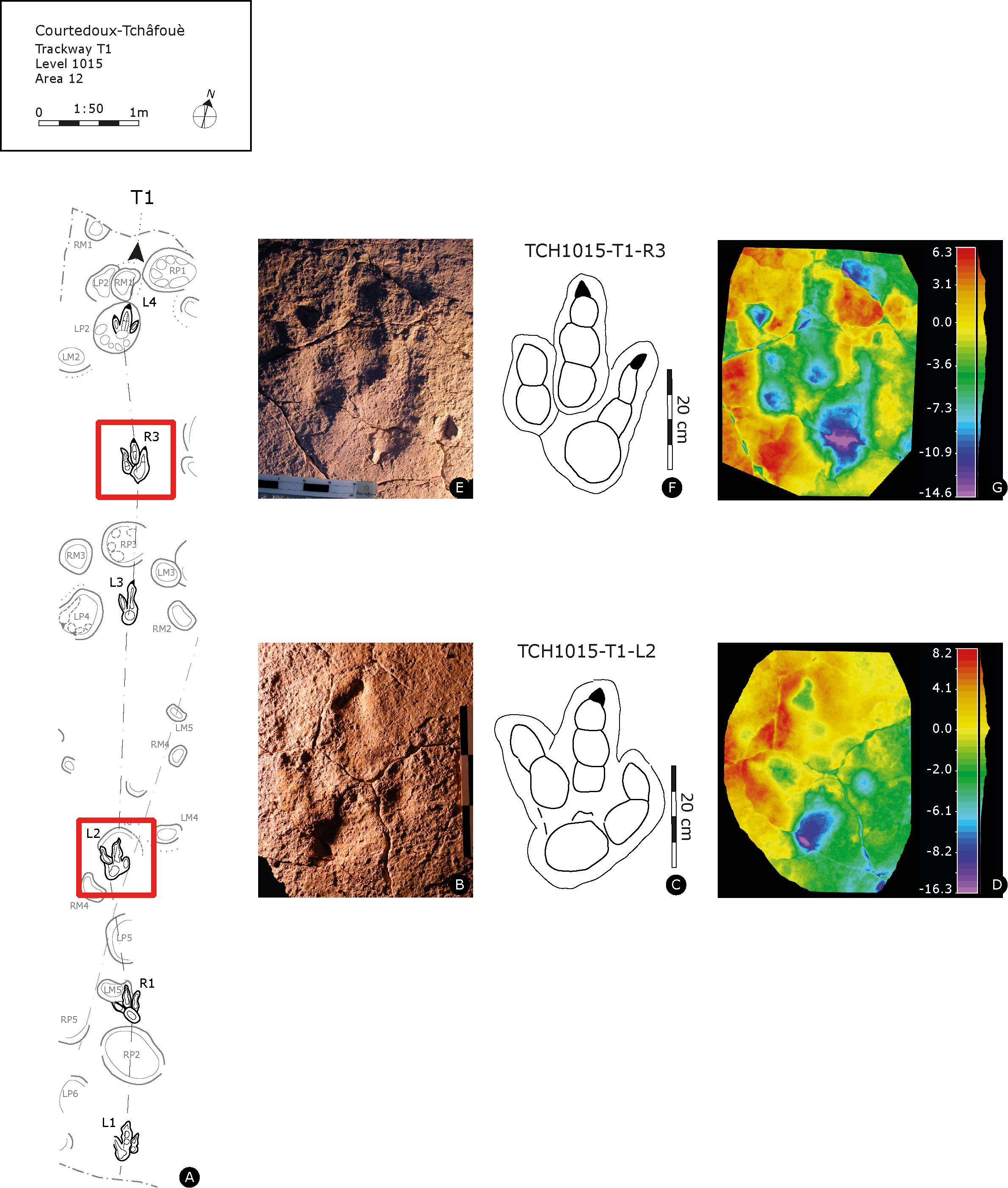

Supplement: S19 Fig — (A) Outline drawing at 1:50 scale of the trackway. (B) Photo of TCH1015-T1-L2. Scale bar 30 cm. (C) Interpretative outline drawing of TCH1015-T1-L2. (D) False-color depth map of TCH1015-T1-L2. Depth measured in mm. (E) Photo of TCH1015-T1-R3. Scale 20 cm. (F) Interpretative outline drawing of TCH1015-T1-R3. (G) False-color depth map of TCH1015-T1-R3. Depth measured in mm. (TIF) [file pone.0180289.s020.tif]

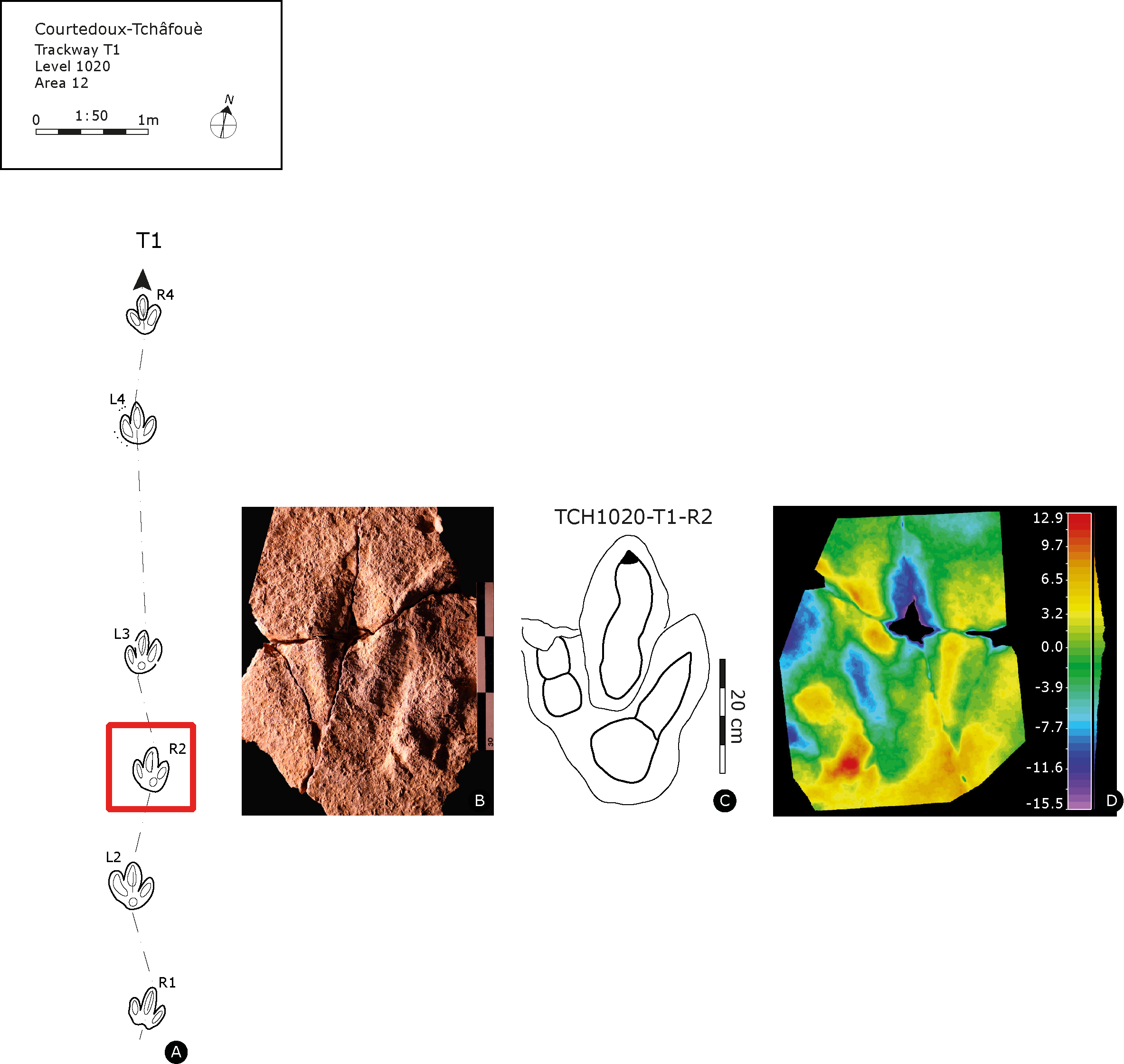

Supplement: S20 Fig — (A) Outline drawing at 1:50 scale of the trackway. (B) Photo of TCH1020-T1-R2. Scale bar 30 cm. (C) Interpretative outline drawing of TCH1020-T1-R2. (D) False-color depth map of TCH1020-T1-R2. Depth measured in mm. (TIF) [file pone.0180289.s021.tif]

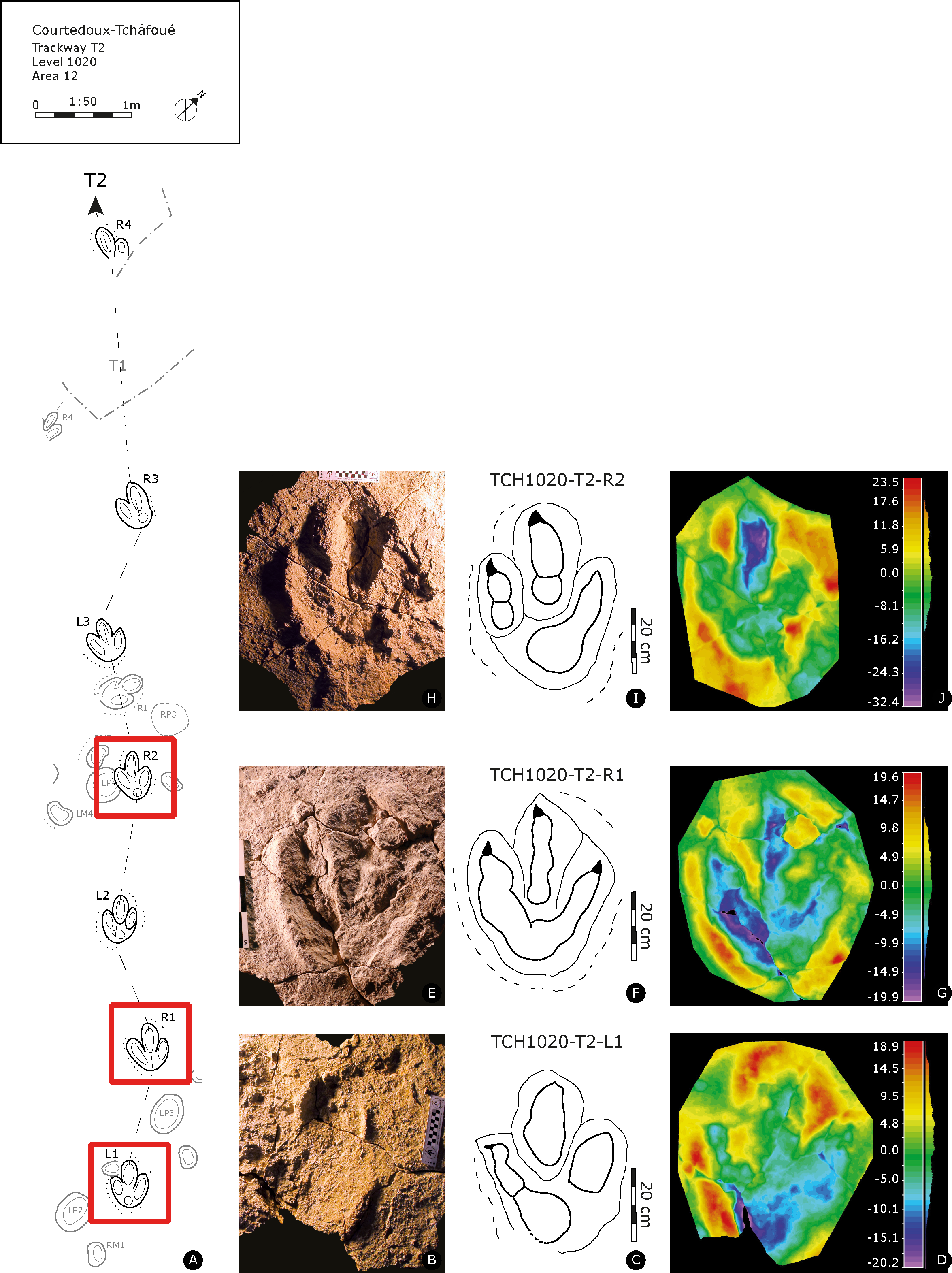

Supplement: S21 Fig — (A) Outline drawing at 1:50 scale of the trackway. (B) Photo of TCH1020-T2-L1. Scale bar 18 cm (10 cm for the black/white scale bar). (C) Interpretative outline drawing of TCH1020-T2-L1. (D) False-color depth map of TCH1020-T2-L1. Depth measured in mm. (E) Photo of TCH1020-T2-R1. Scale bar 20 cm. (F) Interpretative outline drawing of TCH1020-T2-R1. (G) False-color depth map of TCH1020-T2-R1. Depth measured in mm. (H) Photo of TCH1020-T2-R2. Scale bar 18 cm (10 cm for the black/white scale bar). (I) Interpretative outline drawing of TCH1020-T2-R2. (J) False-color depth map of TCH1020-T2-R2. Depth measured in mm. (TIF) [file pone.0180289.s022.tif]

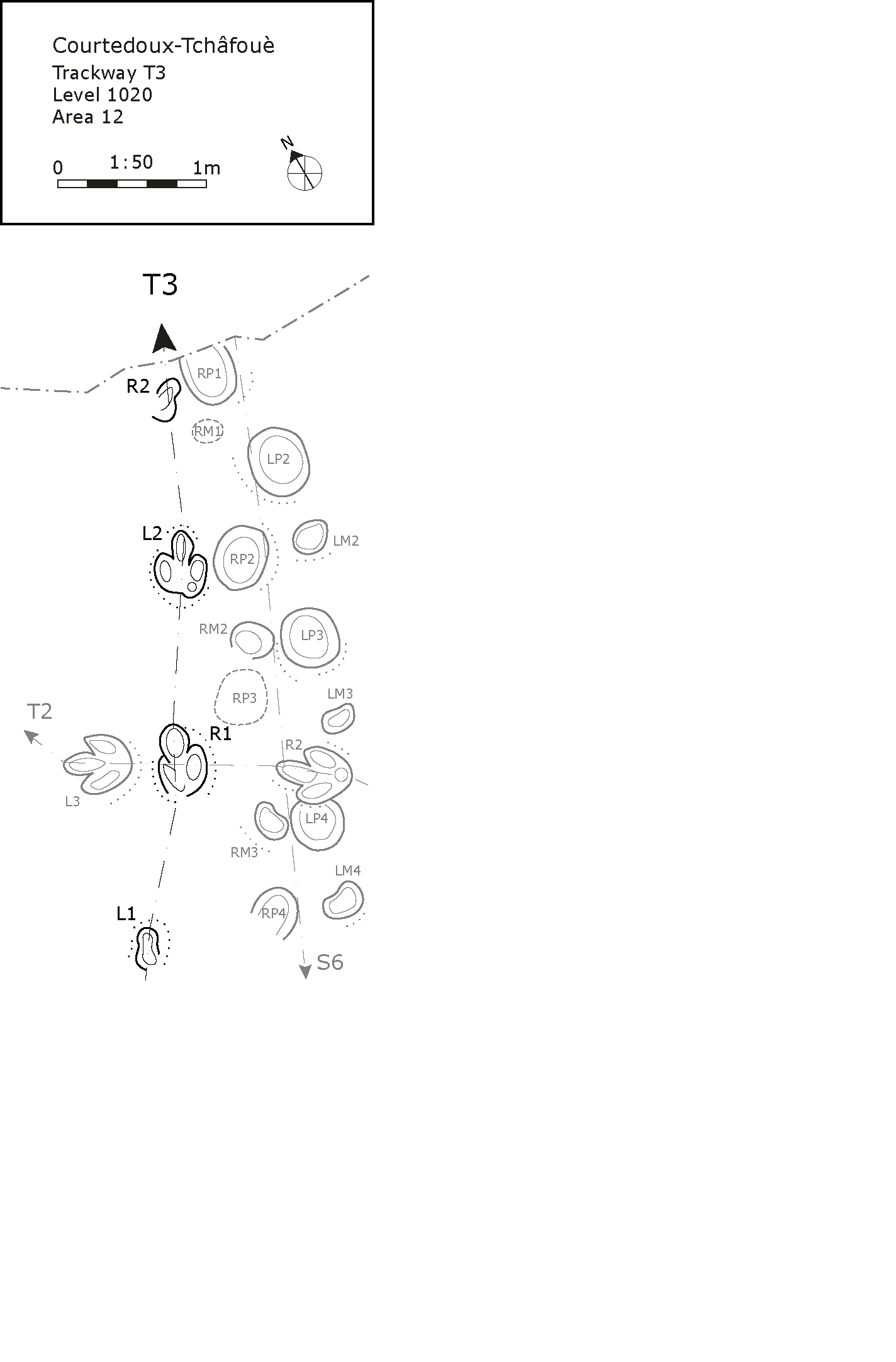

Supplement: S22 Fig — Outline drawing at 1:50 scale of the trackway. (TIF) [file pone.0180289.s023.tif]

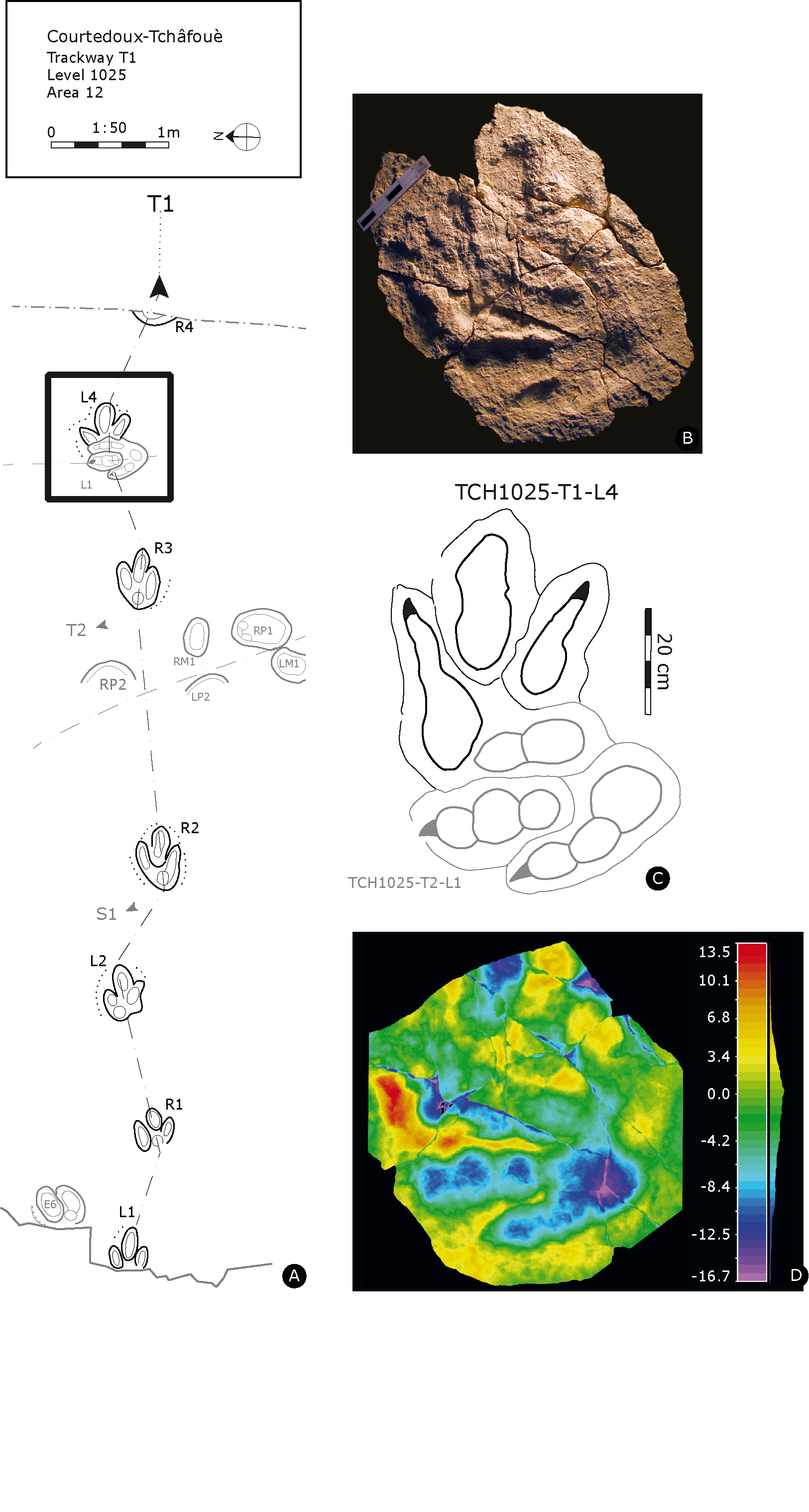

Supplement: S23 Fig — (A) Outline drawing at 1:50 scale of the trackway. (B) Photo of TCH1025-T1-L4. Scale bar 20 cm. (C) Interpretative outline drawing of TCH1025-T2-L1. (D) False-color depth map of TCH1025-T1-L4. Depth measured in mm. (TIF) [file pone.0180289.s024.tif]

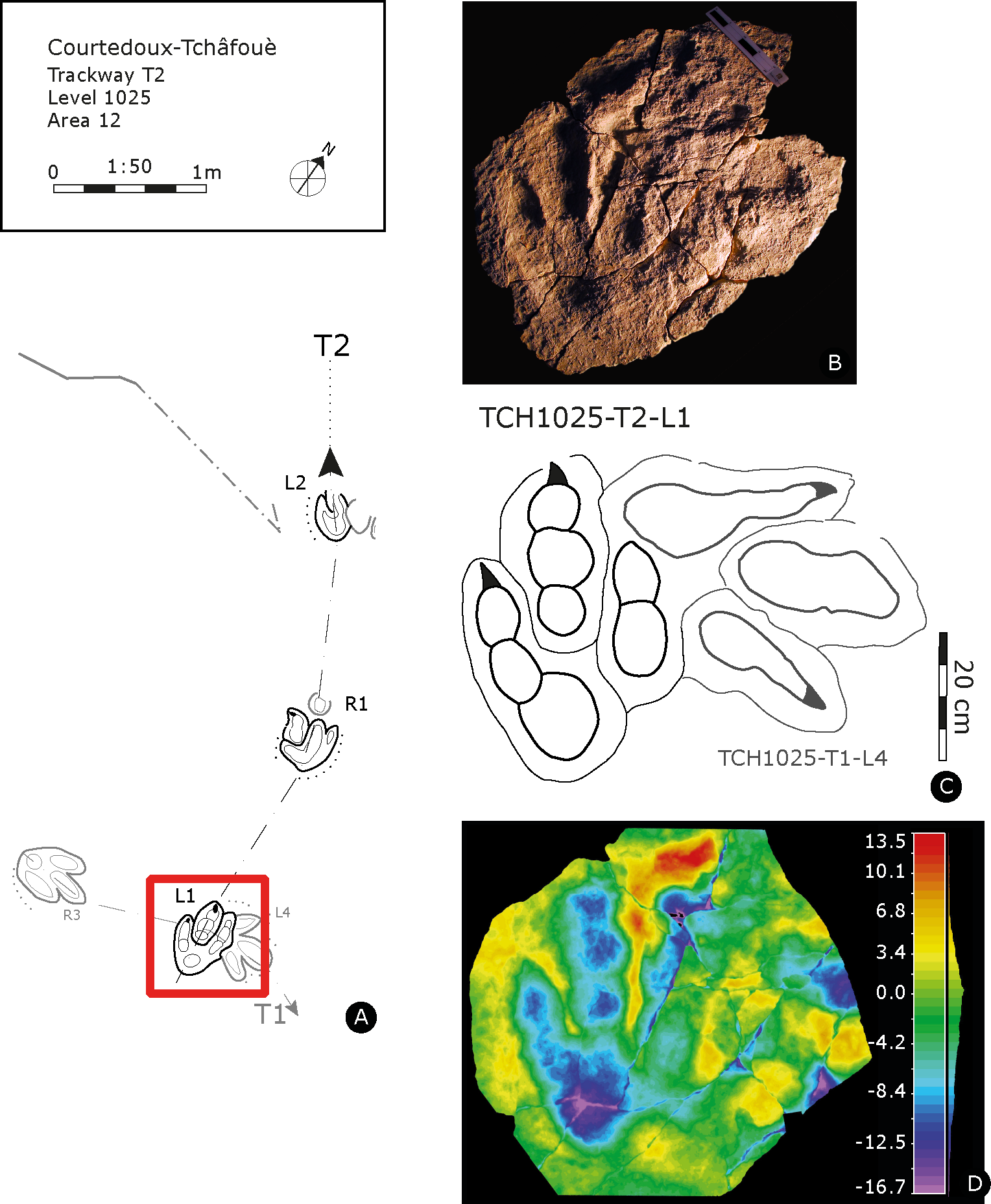

Supplement: S24 Fig — (A) Outline drawing at 1:50 scale of the trackway. (B) Photo of TCH1025-T2-L1 (paratype). Scale bar 20 cm. (C) Interpretative outline drawing of TCH1025-T2-L1. (D) False-color depth map of TCH1025-T2-L1 Depth measured in mm. (TIF) [file pone.0180289.s025.tif]

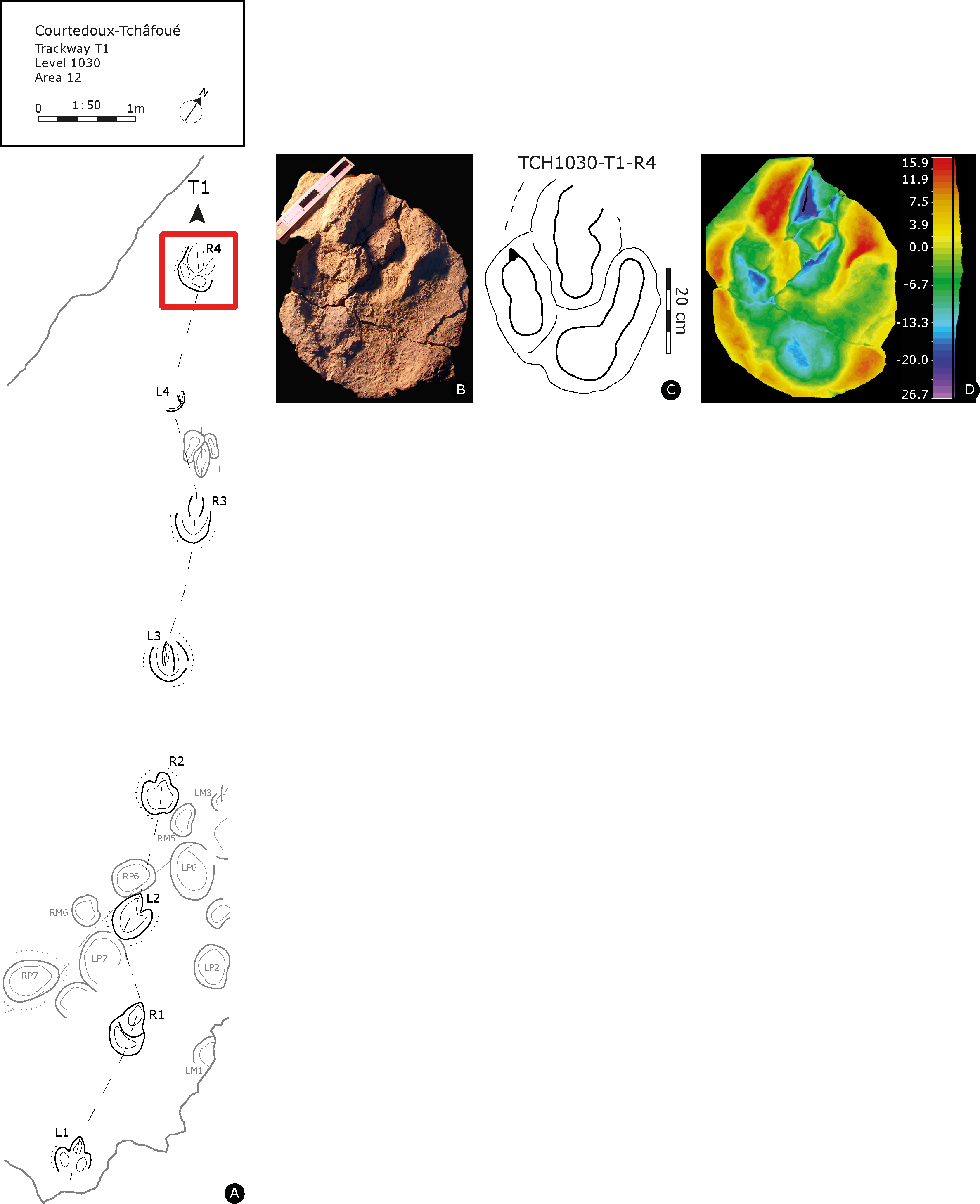

Supplement: S25 Fig — (A) Outline drawing at 1:50 scale of the trackway. (B) Photo of TCH1030-T1-R4. Scale bar 20 cm. (C) Interpretative outline drawing of TCH1030-T1-R4. (D) False-color depth map of TCH1030-T1-R4. Depth measured in mm. (TIF) [file pone.0180289.s026.tif]

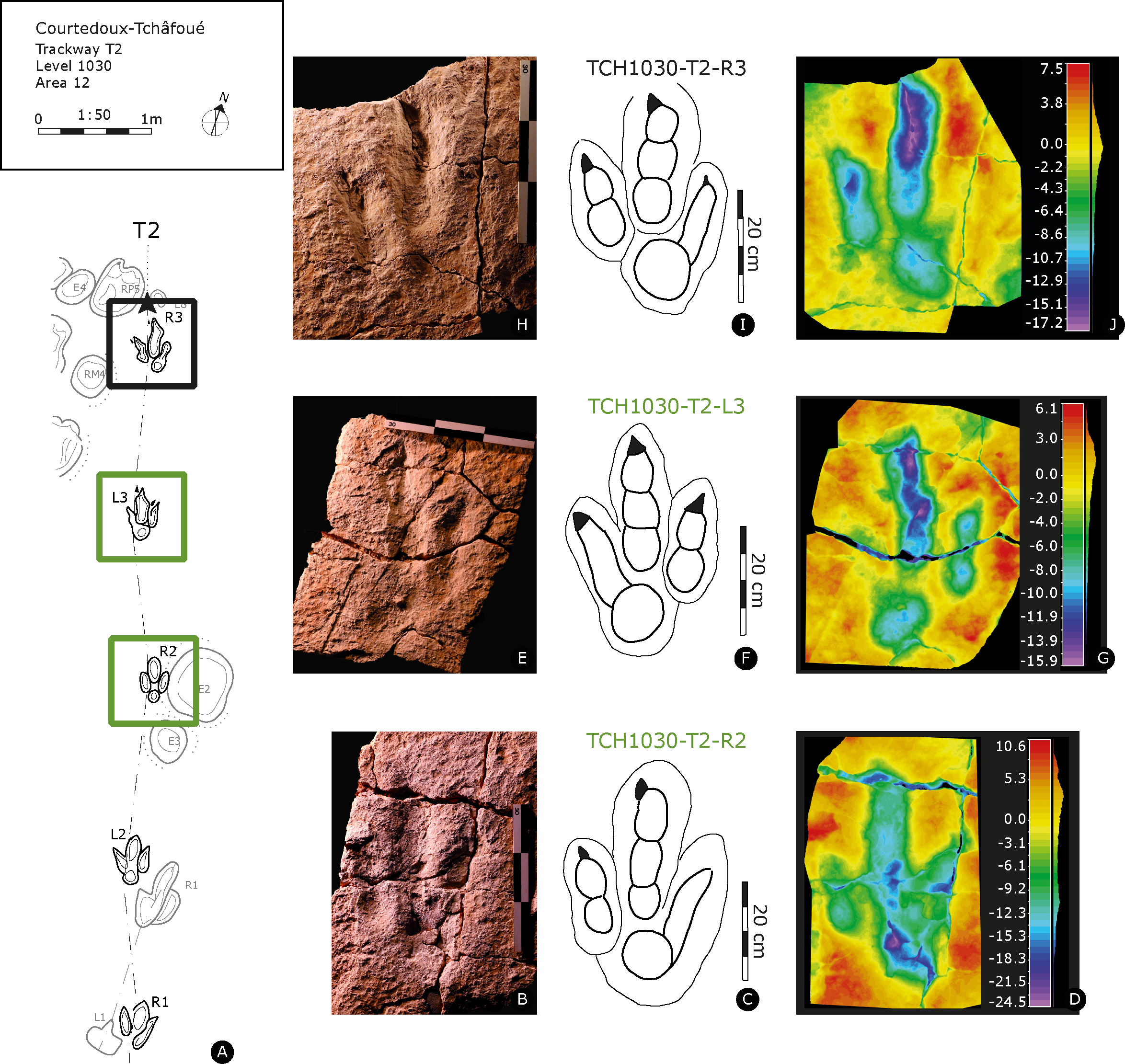

Supplement: S26 Fig — (A) Outline drawing at 1:50 scale of the trackway. (B) Photo of TCH1030-T2-R2 (paratype). Scale bar 30 cm. (C) interpretative outline drawing of TCH1030-T2-R2. (D) False-color depth map of TCH1030-T2-R2. Depth measured in mm. (E) Photo of TCH1030-T2-L3 (paratype). Scale bar 30 cm. (F) Interpretative outline drawing of TCH1030-T2-L3. (G) False-color depth map of TCH1030-T2-L3. Depth measured in mm. (H) Photo of TCH1030-T2-R3. Scale bar 30 cm. (I) Interpretative outline drawing of TCH1030-T2-R3. (J) False-color depth map of TCH1030-T2-R3. Depth measured in mm. (TIF) [file pone.0180289.s027.tif]

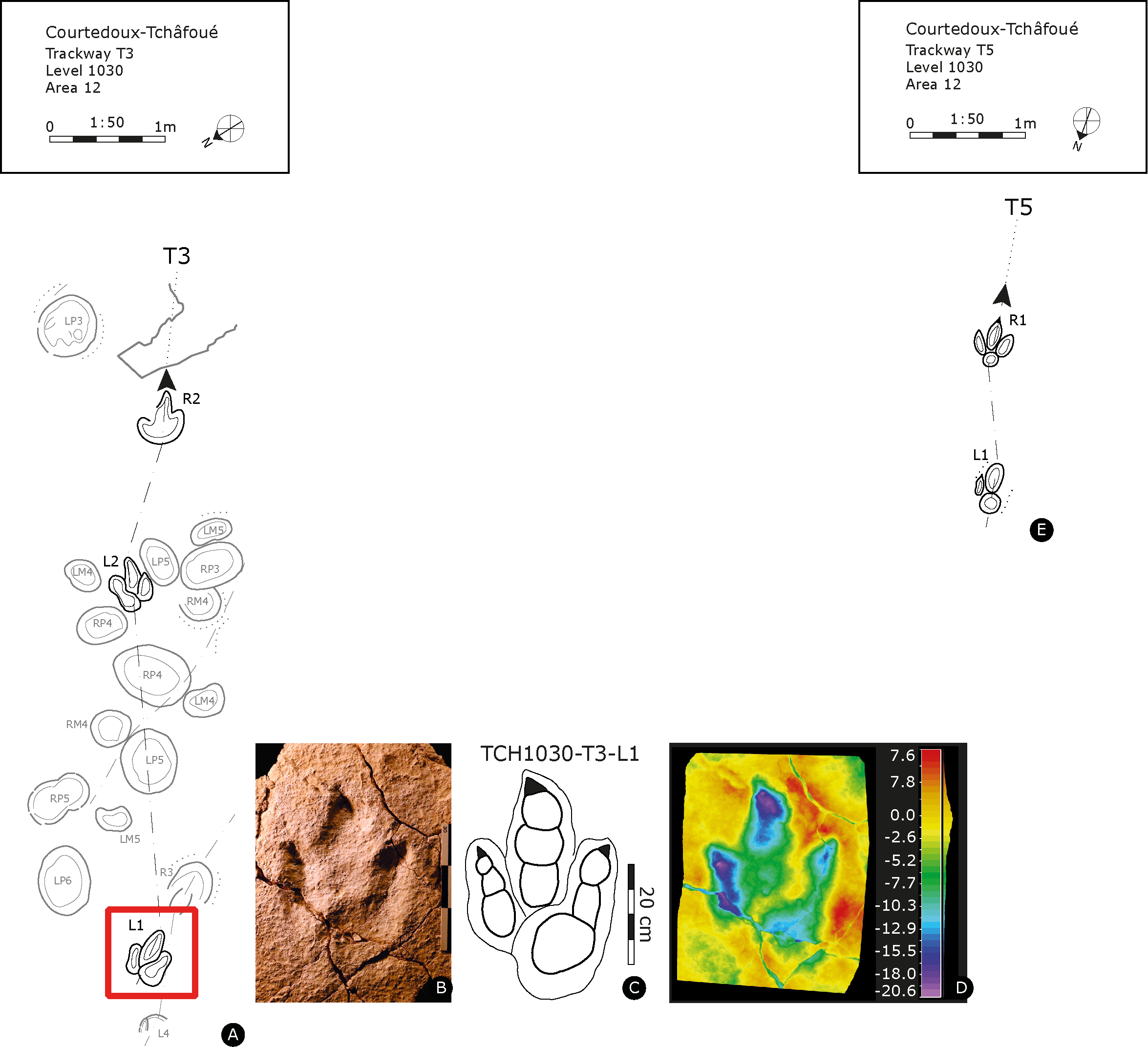

Supplement: S27 Fig — (A) Outline drawing at 1:50 scale of TCH1030-T3. (B) Photo of TCH1030-T3-L1. Scale bar 30 cm. (C) Interpretative outline drawing of TCH1030-T3-L1. (D) False-color depth map of TCH1030-T3-L1. Depth measured in mm. (E) Outline drawing of TCH1030-T3 (scale 1:50). (TIF) [file pone.0180289.s028.tif]

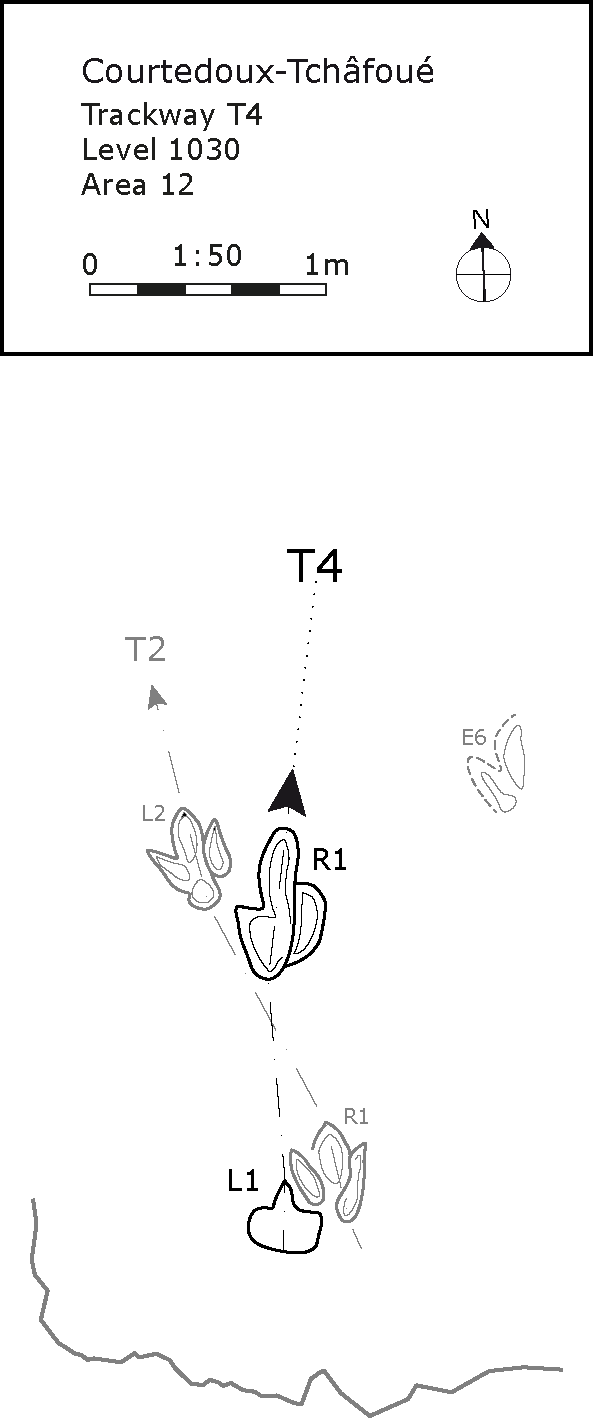

Supplement: S28 Fig — Outline drawing at 1:50 scale of the trackway. (TIF) [file pone.0180289.s029.tif]

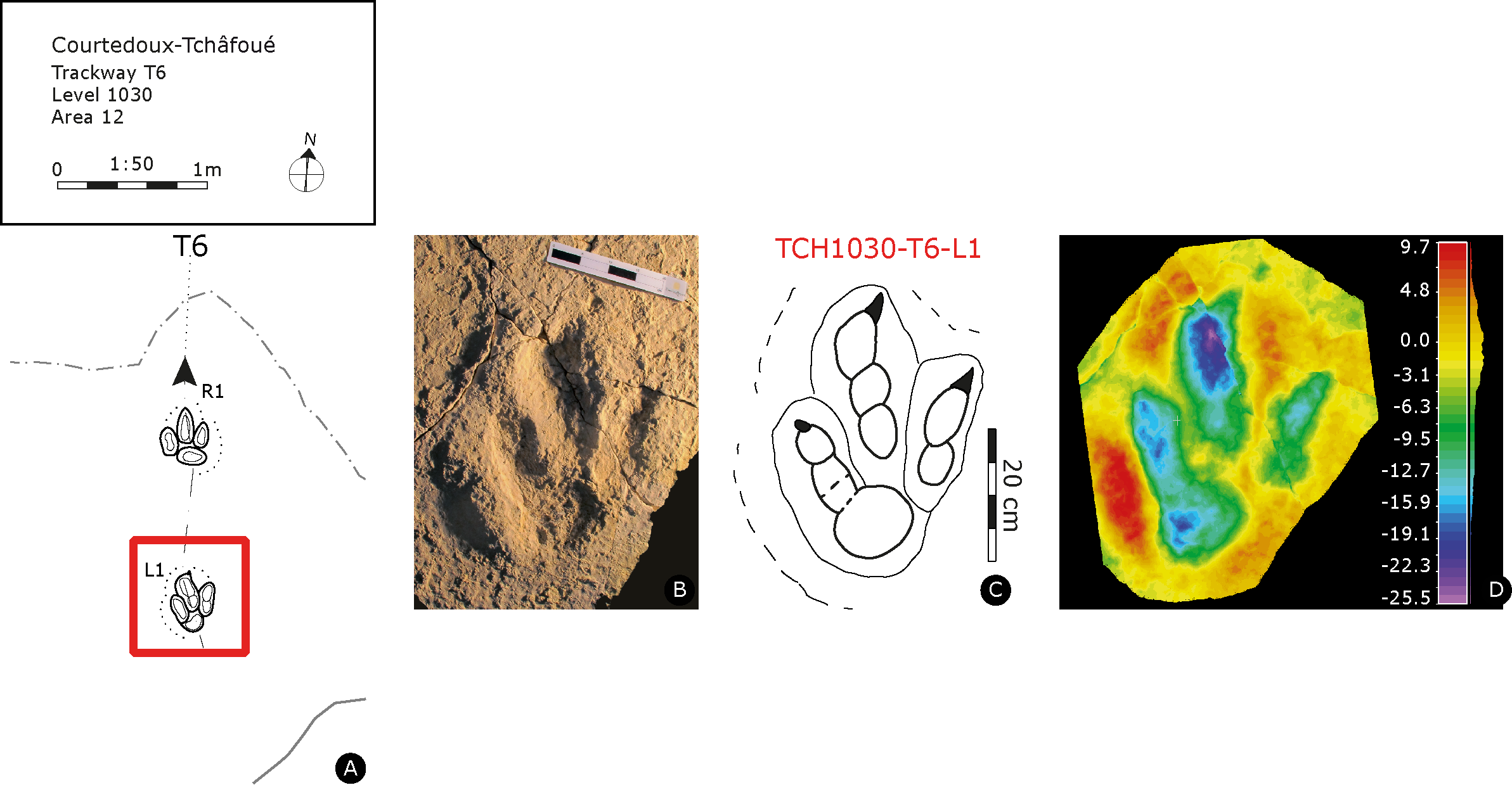

Supplement: S29 Fig — (A) Outline drawing at 1:50 scale of the trackway. (B) Photo of TCH1030-T6-L2 (holotype). Scale bar 30 cm. (C) Interpretative outline drawing of TCH1030-T6-L2. (D) False-color depth map of TCH1030-T6-L2. Depth measured in mm. (TIF) [file pone.0180289.s030.tif]

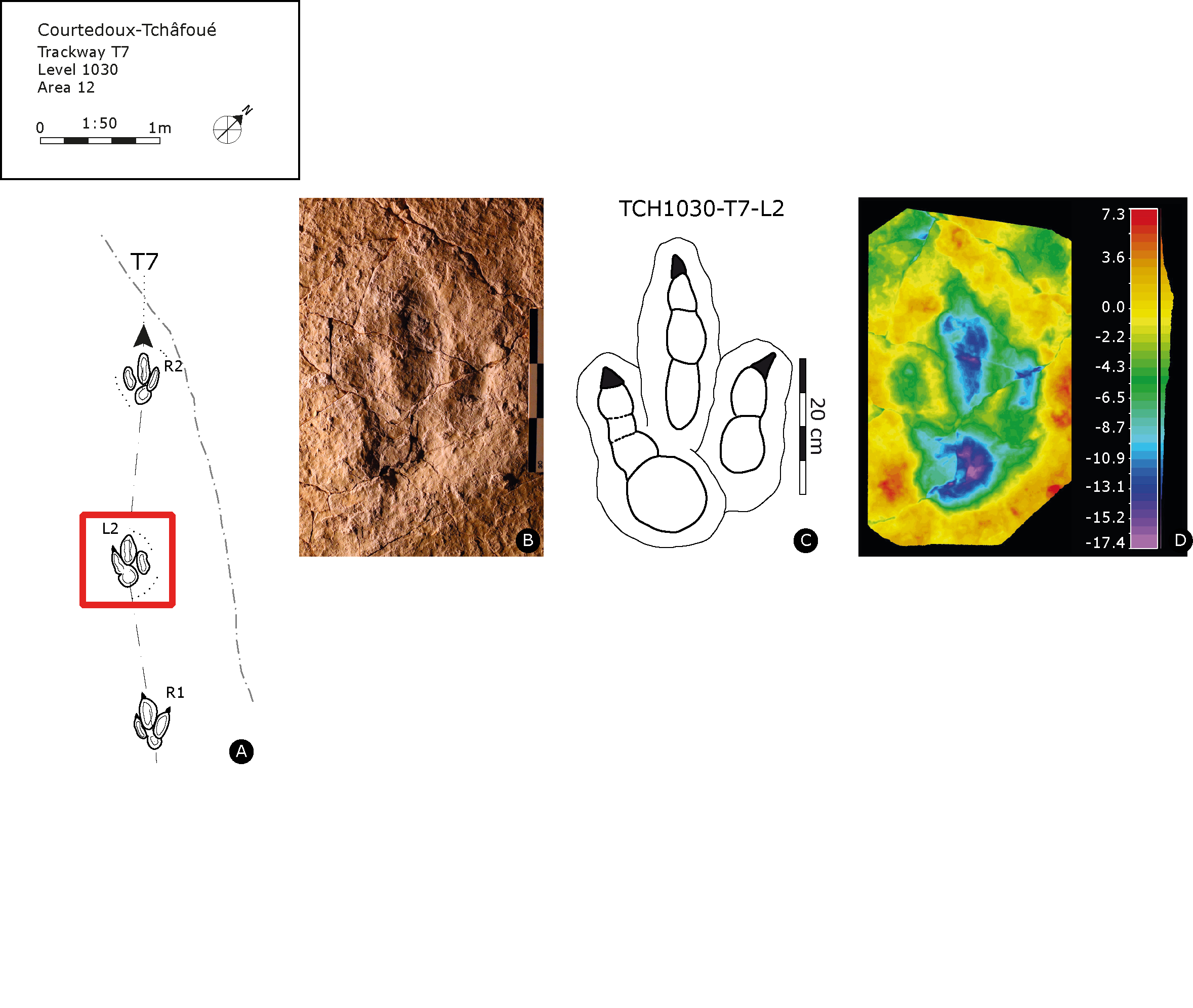

Supplement: S30 Fig — (A) Outline drawing at 1:50 scale of the trackway. (B) Photo of TCH1030-T7-L2 (paratype). Scale bar 30 cm. (C) interpretative outline drawing of TCH1030-T7-L2. (D) False-color depth map of TCH1030-T7-L2. Depth measured in mm. (TIF) [file pone.0180289.s031.tif]
